# Efficient Production of 4’-Hydroxydihydrochalcones Using Non-Conventional Yeast Strains

**DOI:** 10.3390/ijms251910735

**Published:** 2024-10-05

**Authors:** Paweł Chlipała, Julia Bienia, Marcelina Mazur, Monika Dymarska, Tomasz Janeczko

**Affiliations:** Department of Food Chemistry and Biocatalysis, Faculty of Biotechnology and Food Science, Wrocław University of Environmental and Life Sciences, 50-375 Wrocław, Poland; 125375@student.upwr.edu.pl (J.B.); marcelina.mazur@upwr.edu.pl (M.M.); monika.dymarska@upwr.edu.pl (M.D.)

**Keywords:** biotransformation, dihydrochalcones, non-conventional yeasts, natural products, hydrogenation, chalcone

## Abstract

The quest for novel therapeutic agents has rekindled interest in natural products, particularly those derived from biotransformation processes. Dihydrochalcones, a class of plant secondary metabolites, exhibit a range of pharmacological properties. Chalcone and dihydrochalcone compounds with the characteristic 4’-hydroxy substitution are present in ‘dragon’s blood’ resin, known for its traditional medicinal uses and complex composition, making the isolation of these compounds challenging. This study investigates the efficient production of 4′-hydroxydihydrochalcones using non-conventional yeast strains. We evaluated the biotransformation efficiency of various 4′-hydroxychalcone substrates utilizing yeast strains such as *Yarrowia lipolytica* KCh 71, *Saccharomyces cerevisiae* KCh 464, *Rhodotorula rubra* KCh 4 and KCh 82, and *Rhodotorula glutinis* KCh 242. Our findings revealed that *Yarrowia lipolytica* KCh 71, *Rhodotorula rubra* KCh 4 and KCh 82, and *Rhodotorula glutinis* KCh 242 exhibited the highest conversion efficiencies, exceeding 98% within one hour for most substrates. The position of methoxy substituents in the chalcone ring significantly influenced hydrogenation efficiency. Moreover, we observed isomerization of *trans*-4′-hydroxy-2-methoxychalcone to its *cis* isomer, catalyzed by light exposure. This study underscores the potential of using yeast strains for the sustainable and efficient production of dihydrochalcones, providing a foundation for developing new therapeutic agents and nutraceuticals.

## 1. Introduction

The quest for novel therapeutic agents has led to a resurgence of interest in the realm of natural products, particularly those derived from biotransformation processes [1,2]. Among these, dihydrochalcones, a class of secondary plant metabolites, have garnered significant attention due to their multifaceted pharmacological properties [3,4]. Dihydrochalcones are known for their potent antioxidant activity and a spectrum of pharmacological effects, such as antidiabetic, antitumor, lipometabolism-regulating, anti-inflammatory, antibacterial, antiviral, and immunomodulatory properties [5,6,7,8,9]. Their antidiabetic action, in particular, is of paramount importance, given the global rise in diabetes prevalence. These compounds have been shown to exhibit significant therapeutic potential in the management of diabetes, offering a complementary approach to existing treatments [3,10]. The benefits of dihydrochalcones extend beyond their antidiabetic properties. They have been associated with anticancer activities, suggesting a role in chemoprevention. Their antioxidant properties are also noteworthy, as they contribute to the neutralization of free radicals, thereby mitigating oxidative stress: a key factor in the pathogenesis of numerous chronic diseases [5,10,11].

The biotransformation of chalcones into dihydrochalcones in yeast cultures represents a promising avenue for producing these bioactive compounds. Yeasts, as microbial catalysts, offer a sustainable and efficient method for the biotransformation of chalcones, which are otherwise limited by their low solubility and bioavailability in plant sources [9]. The enzymatic machinery within these yeasts facilitates the reduction of α,β-unsaturated ketones present in chalcones, leading to the formation of dihydrochalcones [12,13]. Traditionally, conventional yeast strains such as *Saccharomyces cerevisiae* have been extensively utilized in various industrial biotransformations due to their well-characterized enzymatic systems and robustness in diverse bioprocesses [14,15,16,17]. However, recent advancements have highlighted the potential of non-conventional yeast strains, which exhibit unique metabolic pathways and enzymatic capabilities that can be advantageous for specific biotransformations, including those of chalcones [18,19,20,21].

Non-conventional yeast strains, such as *Yarrowia lipolytica*, *Rhodotorula rubra*, and *Rhodotorula glutinis*, have demonstrated superior efficiency in catalyzing the reduction of α,β-unsaturated ketones present in chalcones, leading to the formation of dihydrochalcones [22,23,24]. These strains are particularly valuable when conventional strains show limited activity or selectivity towards certain substrates, such as chalcones with various substituents [25]. Moreover, non-conventional yeasts can operate under more extreme conditions, tolerating factors like pH variations or higher concentrations of substrates and products, which further enhances their utility in industrial biotransformation processes [26,27,28].

The results obtained by Łużny et al. showed that the enzyme apparatus of the yeast strain *Yarrowia lipolytica* KCh 71 was capable of biotransforming all the tested bromochalcone substrates, but the process was catalyzed at different rates depending on the position of the bromine substituent [25]. The study found that as the number of methoxy groups on the B ring of the chalcone increased, the substrate conversion yield decreased in yeast strain cultures. When there were two methoxy substituents on the A ring of the substrate, biotransformation did not occur [12]. The position of the methoxy substitution also influenced the rate and efficiency of the hydrogenation process. These differences are best seen in the case of the *Rhodotorula glutinis* KCh 242 strain, in whose culture the conversion of compounds containing one methoxy group in different positions (in the same ring) is diametrically different [12]. Another preference, selectivity concerning the substrate structure, was observed for the *Saccharomyces cerevisiae* KCh 464 strain, for which a significantly higher conversion was noted for 2′-hydroxy-4-methoxychalcone. Compounds with the methoxy group located at C-2 and C-3 are converted with significantly lower efficiency in the culture of this strain [12]. This observation was surprising, as strains of this species are described in many studies as effective and universal biocatalysts for hydrogenating double bonds in chalcones [17,23]. Therefore, the search results unequivocally indicate that the position and nature of substituents like methoxy, hydroxy, bromo, etc., in the chalcone ring system can significantly impact the activity, regioselectivity, and stereoselectivity of enoate reductases from different yeast strains towards various chalcone substrates during their biotransformation into dihydrochalcones [24].

This paper aims to assess the possibility of obtaining dihydrochalcones from chalcones with high yields through biotransformations with the use of various yeast cultures, including *Yarrowia lipolytica* KCh 71, *Saccharomyces cerevisiae* KCh 464, *Rhodotorula rubra* KCh 4 and 82, and *Rhodotorula glutinis* KCh 242. In conclusion, the biotransformation of chalcones into dihydrochalcones using yeast cultures presents a viable and eco-friendly strategy for the production of these valuable compounds. Exploring their antidiabetic and other health-promoting effects could pave the way for the development of new functional foods and nutraceuticals, potentially revolutionizing the landscape of natural product-based therapeutics.

## 2. Results and Discussion

**Scheme 1:**
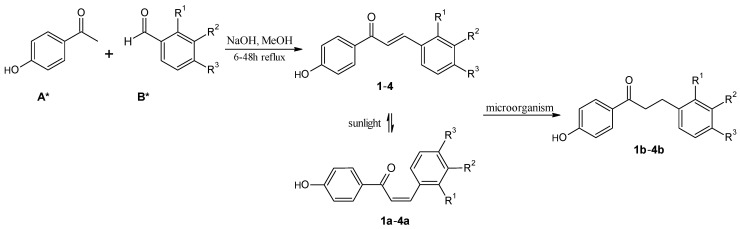
Synthesis of chalcones and their subsequent transformations to dihydrochalcones using unconventional yeast strains.

This study investigated the biotransformation efficiency of various 4’-hydroxychalcone substrates using non-conventional yeast strains, namely, *Yarrowia lipolytica* KCh 71, *Saccharomyces cerevisiae* KCh 464, *Rhodotorula rubra* KCh 4 and KCh 82, *Rhodotorula glutinis* KCh 242, and *Rhodotorula marina* KCh 77. These yeast strains were selected for their ability to efficiently hydrogenate chalcones, as described in our previous work [12,20,22,25]. The primary goal was to find a strain that most effectively transforms the tested 4’-hydroxychalcones into the corresponding dihydrochalcones (Scheme 1 and Table 1). Chalcone and dihydrochalcone compounds with the characteristic 4’-hydroxy substitution are present, among others, in a dark-red resin called ‘dragon’s blood’ [29,30,31]. Dragon’s blood is a deep-red resin obtained from several plant genera, including *Dracaena, Daemonorops*, *Croton*, and *Pterocarpus*. The most commonly studied species include *Dracaena cinnabari*, *Dracaena draco*, and *Dracaena cochinchinensis* [32,33,34]. Various cultures have used dragon’s blood in traditional medicine, including the ancient Chinese, Greek, and Roman civilizations. It continues to be of interest in modern research for its potential therapeutic applications [35]. However, although 4-hydroxychalcones are widely represented in this biological material, they occur in mixtures that make their isolation and purification very difficult. Therefore, we used a combination of chemical and biotechnological methods to obtain these compounds. Chalcones were synthesized via Claisen–Schmidt condensation and subsequently subjected to biotransformation by the selected yeast strains. Scheme 1 illustrates the synthesis route, starting from the chalcones to the resulting dihydrochalcones.

Effective hydrogenation of the applied substrates was observed in all the selected cultures. The conversion rates of the biotransformation processes varied slightly among the different yeast strains. As shown in Table 2, Table 3, Table 4 and Table 5, *Yarrowia lipolytica* KCh 71, *Rhodotorula rubra* KCh 4, and *Rhodotorula glutinis* KCh 242 exhibited highest efficiency in transforming chalcones to their respective dihydrochalcones, with conversion exceeding 98% within 1 h for most substrates. Conversely, *Saccharomyces cerevisiae* KCh 464 showed variable performance, demonstrating high conversion for specific substrates (**1**, **4**) and significantly lower conversion for others (**2**, **3**).

*trans*-4’-Hydroxy-chalcone (**1**) underwent efficient hydrogenation (conversion ≥ 98%) within one hour of the biotransformation process in cultures of most strains used in this study. Only in the culture of *Saccharomyces cerevisiae* KCh 464 was 4’-hydroxy-dihydrochalcone (**1b**) obtained with a conversion rate of 67%. The formation of *cis*-4’-hydroxy-chalcone (**1a**) was also observed (it made up 17% of the reaction mixture). However, after just 6 h, only dihydrochalcone (**1b**) was detected also in the culture of this strain.

In general, methoxy derivatives of 4’-hydroxychalcone (**2**, **3**, and **4**) underwent hydrogenation much more efficiently compared to the analogous 2’-hydroxychalcones (previously described [12]) in the cultures of the studied strains. In this study, significantly smaller differences were observed depending on the biocatalyst used. However, there was a straight dependence of the conversion rate on the position of the methoxy group. 4’-Hydroxy-2-methoxychalcone (**2**) was transformed slower in the cultures of the studied microorganisms. In our previous work, we described significant differences in the conversion of 2-, 3-, and 4-methoxy derivatives of 2’-hydroxychalcone [12]. We demonstrated that the *Yarrowia lipolytica* KCh 71 strain was capable of effectively converting the applied substrates regardless of the position of the methoxy group. The *Rhodotorula glutinis* KCh 242 strain effectively reduced 3- and 4-methoxy-2’-hydroxychalcone but is considerably less active towards the 2-methoxy derivative [12]. In the culture of this strain, significant differences were also observed in the conversion of the 2-, 3-, and 4-bromo derivatives of 2’-hydroxychalcone [25]. It was shown that the 4-bromo derivative underwent the most efficient hydrogenation. Chalcones containing a bromine atom in their structure were efficiently transformed in the culture of *Yarrowia lipolytica* KCh 71. A conversion rate of >95% was achieved for the following: 2-bromo-2’-hydroxychalcone after just 1 h, 3-bromo-2’-hydroxychalcone after 3 h, and 4-bromo-2’-hydroxychalcone after 3 days of transformation [25].

Despite the fact that 4’-hydroxy-2-methoxychalcone (**2**) underwent much slower hydrogenation, the corresponding dihydrochalcone (**2b**) was obtained with a conversion of >90% in the culture of *Rhodotorula glutinis* KCh 242 after 6 h of transformation. More than 60% conversion after 1 h of substrate incubation was also observed in cultures of *Rhodotorula rubra* KCh 4 and *Yarrowia lipolytica* KCh 71. However, in all transformations of 4’-hydroxy-2-methoxychalcone (**2**), a distinct isomerization of the substrate occurs. This resulted in a significant percentage of the *cis* isomer in the reaction mixtures. Such isomerization was not observed in our earlier studies with 2’-hydroxychalcones as substrates [12,20,25]. In contrast, in the case of 4’-hydroxy-chalcones, such isomerization was described early on as photoisomerization [22,36].

Effective hydrogenation of *trans*-4’-hydroxy-3-methoxychalcone (**3**) was observed in most of the tested strains. Only in the culture of the *Saccharomyces cerevisiae* KCh 464 strain was a significant share of untransformed *trans*- and *cis*-4’-hydroxy-3-methoxychalcone (**3** and **3a**) observed after one hour: 35 and 34%, respectively. After 48 h of transformation, 90% of the product (**3b**) was detected. In the cultures of other strains, the process was much more effective, and only trace amounts of unreacted substrate (**3**) and the product of its isomerization (**3a**) were detected. 

The hydrogenation of *trans*-4’-hydroxy-4-methoxychalcone (**4**) was slightly slower in the tested yeast strains’ cultures. For this substrate, 4’-hydroxy-4-methoxydihydrochalcone (**4b**) was the predominant compound already after one hour of transformation. After 3 h, the isomerization product *cis*-4’-hydroxy-4-methoxychalcone (**4a**) was also detected. In the culture of *Saccharomyces cerevisiae* strain KCh 464, even after 48 h, only 2% of this isomer was detected in the reaction mixture.

Because *trans–cis* isomerization was observed in most of the biotransformations, we decided to test various factors that could catalyze this process. *trans*-4’-Hydroxy-2-methoxychalcone (**2**) was used as a substrate because, when interpreting the results obtained from the biotransformation of this compound, the presence of *cis*-4’-hydroxy-2-methoxychalcone (**2a**) was unequivocal. This isomer was also isolated and identified by NMR methods as a result of preparative biotransformations. We carried out a series of experiments to test different factors affecting the isomerization process. All variants were run for one hour. *trans*-4’-Hydroxy-2-methoxychalcone (**2**) was dissolved in several organic solvents (Table 6). We demonstrated that compound **2** does not isomerize due to exposure to solvents, as *cis*-4’-hydroxy-2-methoxychalcone (**2a**) was not detected in light-shielded vials (wrapped in aluminum foil). The influence of UV lamp light and sterilization lamp light also appeared to be a factor slightly affecting isomerization. However, in samples subjected to direct sunlight exposure, we noted most the significant (71–83%) isomerization of *trans*-4’-hydroxy-2-methoxychalcone (**2**) to *cis*-4’-hydroxy-2-methoxychalcone (**2a**). Also, in samples left for 1 h on the laboratory bench (with indirect exposure to sunlight), isomerization of 18–33% was observed. Since the biotransformations were carried out on rotary shakers located in a windowed microbiology laboratory, substrate isomerization may likely be light-catalyzed.

We also tested the effect of exposure time to sunlight on the tested substrates dissolved in DMSO (Table 7). We observed that increasing the exposure time slightly alters the degree of isomerization, which most likely indicates that a compound-specific equilibrium state has been reached. The isomerization of *trans*-4’-hydroxy-4-methoxychalcone (**4**) to *cis*-4’-hydroxy-4-methoxychalcone (**4a**) was the lowest and stabilized at a level not exceeding 40%.

The NMR data of the *trans*-4’-hydroxy-chalcones obtained from the chemical synthesis are presented in Table 8 and in Section 3. We also prepared samples of the studied *trans*-chalcones in DMSO*d-6* and exposed them to sunlight, which allowed us to obtain spectral data of the *cis* isomers of the studied 4’-hydroxy-chalcones. Based on the ^1^H-NMR. ^13^C-NMR, as well as two-dimensional techniques such as HMBC (heteronuclear multiple bond correlation), HMQC (heteronuclear multiple quantum coherence), and COSY (correlation spectroscopy) analyses, the structures of the appropriate *trans*- and *cis*-4’-hydroxy-chalcones and dihydrochalcones were obtained. The spectroscopic data indicate that the photoisomerization of *trans*-chalcone to its *cis* analog did not affect the arrangement of substituents in both aromatic rings. This is evidenced by the coupling constants and chemical shifts of the signals from protons and carbons. The coupling constants (*J*-values) for the α and β protons in trans isomers are generally larger (*J* around 15–16 Hz) due to the larger dihedral angle between these protons in the *trans* configuration. For example, for *trans*-4’-hydroxy-chalcone (**1**), H-α appears at δ 7.92 (d. *J* = 15.6 Hz) and H-β appears at δ 7.68 (d. *J* = 15.6 Hz). In contrast, the coupling constants for the α and β protons in *cis* isomers are smaller (around 12–13 Hz). For *cis*-4’-hydroxy-chalcone (**1a**), H-α appears at δ 6.71 (d. *J* = 13.0 Hz), and H-β appears at δ 6.93 (d. *J* = 13.0 Hz). Moreover, these protons are found more upfield (lower δ value) because of the different electronic environment and the spatial proximity of substituents (double bond) in the *cis* configuration. The signal from the carbonyl carbon usually appears more downfield in *cis* isomers due to the different electronic environment caused by the spatial arrangement of the substituents (Trans (**1**): C=O at δ 187.14; Cis (**1a**): C=O at δ 192.98).

As a result of biotransformation on a larger scale in the culture of the *Yarrowia lipolytica* KCh 71 strain, we also obtained the expected 4’-hydroxy-dihydrochalcones with high yield. The successful hydrogenation of chalcones to dihydrochalcones was confirmed by the disappearance of the characteristic olefin proton signals in the ^1^H NMR spectra and the appearance of signals corresponding to saturated systems. The signals from α and β protons appear as multiplets and are shifted upfield due to the saturation of the double bond (for example, for 4’-hydroxy-dihydrochalcone (**1b**), H-α appears at δ 3.19–3.27 (m) and H-β appears at δ 3.00–3.06 (m)). Alkenyl hydrogens generate a perpendicular magnetic field to the double-bond axis, causing π-bond electrons to circulate. This circulation strengthens the external field at the edges of the double bond and opposes it at the center of the double bond, resulting in significant deshielding of the alkenyl hydrogens [37]. The signals from α and β carbons appear more downfield due to the electron-withdrawing effects of the double bond (for *trans*-4’-hydroxy-chalcone (**1**), C-α appears at δ 122.11 and C-β at δ 142.76). For dihydrochalcones, these carbons are shifted upfield due to the saturation of the double bond, leading to a more shielded environment. For example, in 4’-hydroxy-dihydrochalcone (**1b**), C-α appears at δ 40.20 and C-β appears at δ 30.51. The signal of the carbonyl carbon appears more downfield as the conjugation with the double bond observed in chalcones is lost upon hydrogenation; for 4’-hydroxy-dihydrochalcone (**1b**), C=O appears at δ 198.58.

The biotransformation of chalcones into dihydrochalcones using various yeast strains has been an area of significant research interest, given the pharmacological importance of these compounds. Stompor et al. (2019) provided a comprehensive review of the pharmacological properties and methods of acquiring dihydrochalcones. They highlighted the potent antioxidant activity and antidiabetic effects of dihydrochalcones, emphasizing the importance of efficient production methods and demonstrating that specific yeast strains can produce high yields of dihydrochalcones efficiently [3]. Yeast strains have been effective in transforming hydroxy and methoxy derivatives of chalcones, as well as compounds containing furan and thiophene substituents [20,25,38]. This study focused on the efficiency of non-conventional yeast strains in transforming 4’-hydroxychalcones, achieving high conversion rates with specific strains such as *Yarrowia lipolytica* KCh 71. *Rhodotorula rubra* KCh 4 and KCh 82, and *Rhodotorula glutinis* KCh 242.

Silva et al. (2010) explored the biohydrogenation of chalcones using *Saccharomyces cerevisiae*, highlighting its chemoselective capabilities in a biphasic system [17]. While *S. cerevisiae* showed high conversion rates for some substrates, our study found it had variable performance, especially with substrates containing methoxy groups [17,39]. This variability underscores the need for strain-specific optimization, as demonstrated by the superior performance of *Yarrowia lipolytica* and *Rhodotorula* sp. strains in our research. Filippucci et al. (2020) examined the use of non-conventional yeasts as sources of ene-reductases for chalcone bioreduction [24]. They identified several strains capable of efficient biotransformation, but also noted the significant impact of substrate structure on conversion efficiency [24]. This aligns with our findings that the position of methoxy groups on the chalcone ring significantly affects the hydrogenation process, with certain positions leading to slower transformation rates.

The position of methoxy groups on the chalcone ring significantly influenced the hydrogenation efficiency. For instance, *trans*-4’-hydroxy-2-methoxychalcone (**2**) underwent slower transformation than other derivatives. This trend was consistent across various strains, suggesting that substituent position critically affects biocatalytic activity. However, Łużny et al. (2020) reported that *Yarrowia lipolytica* KCh 71 consistently performed well regardless of the methoxy group position, whereas *Rhodotorula glutinis* KCh 242 showed variable activity depending on methoxy group positioning [12].

A notable observation in our study was the isomerization of *trans*-4’-hydroxy-chalcones (**1**, **2**, **3**, and **4**) to their *cis* isomers during the biotransformation process. This isomerization was significant in all transformations, highlighting a possible photochemical effect. Our experiments indicated that light exposure, particularly sunlight, significantly catalyzed the isomerization, suggesting that ambient light conditions in the laboratory could influence the biotransformation outcomes. This phenomenon aligns with findings by other authors, who reported similar photochemical isomerization in related compounds [36,40].

This study employed a combination of TLC, GC, and NMR analyses to monitor and confirm the biotransformation products. The preparative-scale biotransformations yielded high-purity dihydrochalcones, with 4’-hydroxy-dihydrochalcone (**1b**) and other methoxy derivatives successfully isolated and characterized. The NMR analysis provided detailed insights into the structural characteristics of the synthesized compounds, confirming the successful hydrogenation and the specific positions of functional groups.

Future research should further optimize the biotransformation conditions, including exploring other non-conventional yeast strains and investigating the mechanisms underlying strain-specific activity. Additionally, addressing the isomerization issue through controlled light conditions or protective measures can enhance the consistency and yield of the desired biotransformation products. Overall, this study provides a comprehensive framework for the efficient production of dihydrochalcones using biotechnological methods, paving the way for sustainable and scalable production of these valuable compounds.

## 3. Material and Methods

### 3.1. Substrates

The substrates used for biotransformation were obtained via Claisen–Schmidt condensation reaction of 4-hydroxyacetophenone (A*) with benzaldehyde-containing methoxylated group(s) in appropriate positions (B*) [purchased from Sigma-Aldrich (St. Louis, MO, USA)]. The general procedure for the synthesis of chalcones was as follows: 50 mmoles of acetophenone and 50 mmoles of benzaldehyde were dissolved in 150 mL of methanol in a 500 mL round-bottom flask. To the reaction mixture, 30 mL of water was added, followed by the gradual addition of 4.0 g of sodium hydroxide (NaOH). The reaction mixture was then heated under reflux for 48 h. The progress of the reaction was monitored by thin-layer chromatography (TLC). Upon completion of the reaction, the mixture was poured into a 1 L beaker and brought to neutral pH with 1 M HCl. The mixture was thoroughly stirred to ensure complete precipitation of the chalcone and was then allowed to stand for 24 h. The crude product was obtained by vacuum filtration using a Büchner funnel. To obtain a high-purity product, the precipitate was recrystallized from ethanol. The crude chalcone was dissolved in ethanol under reflux; then, the solution was cooled to room temperature and left undisturbed for 24 h. The crystallized chalcone was collected via vacuum filtration using a Büchner funnel and dried, and its purity and structure were confirmed using an NMR analysis. The resulting compounds (**1**–**4**) were used as substrates for the biotransformation.

### 3.2. Microorganisms

This research was carried out on six strains of yeast from the following species: *Rhodotorula rubra* (KCh 4 and KCh 82), *Rhodotorula marina* KCh 77, *Rhodotorula glutinis* KCh 242, *Yarrowia lipolytica* KCh 71, and *Saccharomyces cerevisiae* KCh 464, for which storage and biocatalytic capacity have been previously described [20,25,41]. All strains were obtained from the collection of the Department of Chemistry, Wrocław University of Environmental and Life Sciences (Wrocław, Poland). All the yeast strains were stored on agar slants at 4 °C. Using a sterilized inoculation loop, the yeast cells were transferred from the agar slants into 300 mL conical flasks containing 100 mL of sterile liquid medium. The cultures were then incubated at 25 °C for 72 h. This process was used to prepare pre-incubation cultures of the studied strains.

### 3.3. Screening

A 0.5 mL inoculum of the tested yeast strains from the pre-incubation culture was transferred to 300 mL Erlenmeyer flasks for analytical-scale biotransformation. Each flask contained 100 mL of Sabouraud culture medium (3% glucose, 1% peptone) and was incubated for three days at 24 °C on a rotary shaker set to 140 rpm. After this time, 10 mg of the substrate was dissolved in 1 mL of DMSO (dimethyl sulfoxide) and added to the biocatalyst culture. Samples were collected after 1, 3, 6, 12, 24, and 48 h. Portions of 10 mL of the transformation mixture were taken out and extracted with 10 mL of ethyl acetate. The extracts were dried over MgSO_4_, concentrated in vacuo and analyzed using gas chromatography (GC) and thin-layer chromatography (TLC).

### 3.4. Gas Chromatography

A GC analysis was performed using an Agilent 7890A gas chromatograph, equipped with a flame ionization detector (FID) (Agilent. Santa Clara, CA, USA). The capillary column DB-5HT (30 m × 0.25 mm × 0.10 µm) was used to separate the product mixtures. A temperature program was applied as follows: 80–300 °C, temperature on the detector: 300 °C, injection: 1 µL, flow: 1 mL/min, flow H2: 35 mL/min, airflow: 300 mL/min, time of analysis: 18.67 min. During the GC analysis, each compound in the reaction mixture produced a distinct peak on the chromatogram, corresponding to its retention time. The identity of each peak was determined by comparing the retention times with those of standards characterized compounds using NMR analysis. The calculation of percentage composition was performed as follows: The area under each peak in the chromatogram was integrated using the GC software. This area is directly proportional to the concentration of the compound in the sample. The percentage composition of each compound in the reaction mixture was calculated by dividing the area of that compound’s peak by the total area of all peaks in the chromatogram. This allows for the assessment of the efficiency of the biotransformation process and the extent of conversion of substrates to products. The retention time of the substrates and products have been described in Table 9.

### 3.5. Preparative Scale

Preparative biotransformations were performed in 2 L Erlenmeyer flasks, each containing 500 mL of culture medium (3% glucose, 1% peptone). The microorganism (*Yarrowia lipolytica* KCh 71) was incubated for three days at 25 °C on a rotary shaker. After this time, 100 mg of the substrate dissolved in 2 mL of DMSO was added. After seven days, the product was isolated by triple extraction with ethyl acetate (3 extractions with 300 mL) dried with anhydrous magnesium sulfate, and concentrated in vacuo. The transformation products were separated by preparative TLC and analyzed (TLC, GC, and NMR).

### 3.6. Photo-Isomerization Procedure

*trans*-4′-Hydroxy-2-methoxychalcone (**2**) was dissolved in several organic solvents, including ethanol, acetone, tetrahydrofuran (THF), methylene chloride, ethyl acetate, dimethyl sulfoxide (DMSO), methanol, and acetonitrile. Each solution was prepared at a concentration of 2 mg in 1 mL. The prepared solutions were subjected to different light conditions to induce isomerization. Direct Sunlight: Samples were placed under direct sunlight for 1 h. Diffused Light: Samples were left on a laboratory bench with indirect exposure to sunlight for 1 h. UV Lamp Exposure: Samples were exposed to UV light at 365 nm or 254 nm for 1 h. Shielding from Light: Control samples were wrapped in aluminum foil to prevent any light exposure and were kept under identical conditions (solvent, concentration, and temperature) as the exposed samples. Additional experiments were conducted to assess the time dependency of isomerization. The samples of all tested *trans*-chalcones (**1**–**4**) in DMSO were exposed to sunlight for 30 min and 1 h 30 min. After exposure, the degree of isomerization from *trans* to *cis* was analyzed using gas chromatography (GC). The composition of each sample was determined by comparing the relative amounts of *trans*- and *cis*-4′-hydroxy-2-methoxychalcone (**2** and **2a**) and the other tested compounds (**1**, **3**, **4** and **1a**, **3a**, **4a**). The detailed results of these experiments are presented in Table 6 and Table 7 of this manuscript. The *trans*-chalcones obtained via chemical synthesis and their *cis* analogs obtained by isomerization were subjected to NMR analysis.

### 3.7. TLC and NMR Analysis

The course of biotransformation was monitored using TLC plates (SiO_2_, DC Alufolien Kieselgel 60 F254 (0.2 mm thick), Merck, Darmstadt, Germany). The products were separated using preparative TLC plates (Silica Gel GF, 20 × 20 cm, 500 μm, Analtech, Newark, (DE), USA) and developed in a mixture of cyclohexane and ethyl acetate (9:1, *v*/*v*) as the eluent. The product was observed (without additional visualization) under the UV lamp at a wavelength of 254 nm.

The NMR analysis was performed using a DRX 600 MHz Bruker spectrometer (Bruker, Billerica, MA, USA). The prepared samples were dissolved in deuterated DMSO and chloroform CDCl_3_. The performed analyses include ^1^H-NMR, ^13^C-NMR, HMBC (two-dimensional analysis), HMQC (heteronuclear correlation), and COSY (correlation spectroscopy) (all NMR spectra are in Supplementary Materials).

### 3.8. H NMR Data of the Obtained Compounds

*trans*-4’-hydroxy-chalcone (**1**)

^1^H NMR (600 MHz; DMSO-*d*_6_) δ (ppm): 10.44 (s. 1H. C-4’-O*H*). 8.05–8.10 (m. 2H. H-2’ and H-6’). 7.92 (d. 1H. *J* = 15.6 Hz. H-α). 7.85-7.89 (m. 2H. H-2 and H-6). 7.68 (d. 1H. *J* = 15.6 Hz. H-β). 7.42-7.47 (m. 3H. H-3. H-4 and H-5). 6.88-6.92 (m. 2H. H-3’ and H-5’).

*cis*-4’-hydroxy-chalcone (**1a**)

^1^H NMR (600 MHz; DMSO-*d*_6_) δ (ppm): 10.48 (s. 1H. C-4’-O*H*). 7.81–7.83 (m. 2H. H-2’ and H-6’). 7.32-7.36 (m. 2H. H-2 and H-6). 7.23-7.28 (m. 3H. H-3. H-4 and H-5). 6.93 (d. 1H. *J* = 13.0 Hz. H-β). 6.80-6.84 (m. 2H. H-3’ and H-5’). 6.71 (d. 1H. *J* = 13.0 Hz. H-α).

4’-hydroxy-dihydrochalcone (**1b**)

^1^H NMR (600 MHz; CDCl_3_) δ (ppm): 9.24 (s. 1H. C-4’-O*H*). 7.85–7.91 (m. 2H. H-2’ and H-6’). 7.16-7.32 (m. 5H. H-2. H-2. H-4. H-5 and H-6). 6.85-6.89 (m. 3H. H-5. H-3’ and H-5’). 3.19-3.27 (m. 1H. H-α). 3.00-3.06 (m. 1H. H-β).

*trans*-4’-hydroxy-2-methoxychalcone (**2**)

^1^H NMR (600 MHz; DMSO-*d*_6_) δ (ppm): 10.42 (s. 1H. C-4’-O*H*). 8.02–8.06 (m. 2H. H-2’ and H-6’). 7.99 (d. 1H. *J* = 15.6 Hz. H-β). 7.94 (dd. 1H. *J* = 7.7. 1.6 Hz. H-6). 7.85 (d. 1H. *J* = 15.8 Hz. H-α). 7.43 (ddd. 1H. *J* = 8.2. 7.5. 1.6 Hz. H-4). 7.10 (d. 1H. *J* = 8.2 Hz. H-3). 7.02 (t. 1H. *J* = 7.5 Hz. H-5). 6.87-6.92 (m. 2H. H-3’ and H-5’). 3.89 (s. 3H. C-2-OC*H*_3_).

*cis*-4’-hydroxy-2-methoxychalcone (**2a**)

^1^H NMR (600 MHz; DMSO-*d*_6_) δ (ppm): 10.41 (s. 1H. C-4’-O*H*). 7.75–7.78 (m. 2H. H-2’ and H-6’). 7.22 (ddd. 1H. *J* = 7.8. 7.5. 1.6 Hz. H-4). 7.15 (dd. 1H. *J* = 7.5. 1.6 Hz. H-6). 7.04 (d. 1H. *J* = 12.8 Hz. H-β). 6.94 (d. 1H. *J* = 7.8 Hz. H-3). 6.77-6.80 (m. 2H. H-3’ and H-5’). 6.76 (t. 1H. *J* = 7.5. 0.6 Hz. H-5). 6.70 (d. 1H. *J* = 12.8 Hz. H-α). 3.71 (s. 3H. C-2-OC*H*_3_).

4’-hydroxy-2-methoxydihydrochalcone (**2b**)

^1^H NMR (600 MHz; DMSO-*d*_6_) δ (ppm): 10.33 (s. 1H. C-4’-O*H*). 7.83–7.86 (m. 2H. H-2’ and H-6’). 7.18 (td. 1H. *J* = 7.4. 1.7 Hz. H-4). 7.17 (dd. 1H. *J* = 7.4. 0.7 Hz. H-6). 6.94 (dd. 1H. *J* = 8.6. 0.8 Hz. H-3). 6.85 (td. 1H. *J* = 7.4. 1.0 Hz. H-5). 6.81-6.84 (m. 2H. H-3’ and H-5’). 3.78 (s. 3H. C-2-OC*H*_3_). 3.12-3.16 (m. 2H. H-α). 2.83-2.87 (m. 2H. H-β).

*trans*-4’-hydroxy-3-methoxychalcone (**3**)

^1^H NMR (600 MHz; DMSO-*d*_6_) δ (ppm): 10.43 (s. 1H. C-4’-O*H*). 8.04-8.12 (m. 2H. H-2’ and H-6’). 7.92 (d. 1H. *J* = 15.6 Hz. H-α). 7.65 (d. 1H. *J* = 15.6 Hz. H-β). 7.46 (t. 1H. *J* = 1.9 Hz. H-2). 7.41 (d. 1H. *J* = 7.7 Hz. H-6). 7.36 (t. 1H. *J* = 7.8 Hz. H-5). 7.01 (ddd. 1H. *J* = 8.0. 2.4. 0.8 Hz. H-4). 6.88-6.92 (m. 2H. H-3’ and H-5’). 3.83 (s. 3H. C-3-OC*H*_3_).

*cis*-4’-hydroxy-3-methoxychalcone (**3a**)

^1^H NMR (600 MHz; DMSO-*d*_6_) δ (ppm): 10.49 (s. 1H. C-4’-O*H*). 7.80–7.83 (m. 2H. H-2’ and H-6’). 7.17 (t. 1H. *J* = 7.9 Hz. H-5). 7.46 (dd. 1H. *J* = 2.2. 1.8 Hz. H-2). 6.89–6.91 (m. 1H. H-6). 6.89 (d. 1H. *J* = 13.0 Hz. H-β). 6.81-6.84 (m. 2H. H-3’ and H-5’). 6.81 (ddd. 1H. *J* = 8.1. 2.6. 0.7 Hz. H-4). 6.68 (d. 1H. *J* = 13.0 Hz. H-α). 3.63 (s. 3H. C-3-OC*H*_3_).

4’-hydroxy-3-methoxydihydrochalcone (**3b**)

^1^H NMR (600 MHz; DMSO-*d*_6_) δ (ppm): 10.36 (s. 1H. C-4’-O*H*). 7.84–7.88 (m. 2H. H-2’ and H-6’). 7.17 (t. 1H. *J* = 7.9 Hz. H-5). 6.81–6.85 (m. 4H. H-2. H-6. H-3’ and H-5’). 6.73 (ddd. 1H. *J* = 8.0. 2.4. 0.8 Hz. H-4). 3.72 (s. 3H. C-3-OC*H*_3_). 3.21-3.25 (m. 2H. H-α). 2.86–2.89 (m. 2H. H-β). ^1^H NMR (600 MHz; CDCl_3_) δ (ppm): 7.87-7.91 (m. 2H. H-2’ and H-6’). 7.21 (t. 1H. *J* = 7.9 Hz. H-5). 7.04 (s. 1H. C-4’-O*H*). 6.87-6.90 (m. 2H. H-3’ and H-5’). 6.83 (t. 1H. *J* = 7.6 Hz. H-6). 7.46 (dd. 1H. *J* = 2.1. 1.6 Hz. H-2). 6.75 (dd. 1H. *J* = 8.2. 2.5 Hz. H-4). 3.79 (s. 3H. C-3-OC*H*_3_). 3.22-3.28 (m. 2H. H-α). 3.00–3.06 (m. 2H. H-β).

*trans*-4’-hydroxy-4-methoxychalcone (**4**)

^1^H NMR (600 MHz; DMSO-*d*_6_) δ (ppm): 10.41 (s. 1H. C-4’-O*H*). 8.03–8.06 (m. 2H. H-2’ and H-6’). 7.79–7.83 (m. 2H. H-2 and H-6). 7.76 (d. 1H. *J* = 15.5 Hz. H-α). 7.65 (d. 1H. *J* = 15.5 Hz. H-β). 6.98–7.02 (m. 2H. H-3 and H-5). 6.87–6.91 (m. 2H. H-3’ and H-5’). 3.81 (s. 3H. C-4-OC*H*_3_).

*cis*-4’-hydroxy-4-methoxychalcone (**4a**)

^1^H NMR (600 MHz; DMSO-*d*_6_) δ (ppm): 10.42 (s. 1H. C-4’-O*H*). 7.82–7.85 (m. 2H. H-2’ and H-6’). 7.42-7.45 (m. 2H. H-2 and H-6). 6.86 (d. 1H. *J* = 12.8 Hz. H-β). 6.83–6.86 (m. 2H. H-3 and H-5). 6.81–6.84 (m. 2H. H-3’ and H-5’). 6.61 (d. 1H. *J* = 12.8 Hz. H-α). 3.72 (s. 3H. C-4-OC*H*_3_).

4’-hydroxy-4-methoxydihydrochalcone (**4b**) 

^1^H NMR (600 MHz; CDCl_3_) δ (ppm): 7.87-7.91 (m. 2H. H-2’ and H-6’). 7.13–7.17 (m. 2H. H-2 and H-6). 7.00 (s. 1H. C-4’-O*H*). 6.87–6.91 (m. 2H. H-3’ and H-5’). 6.82–6.85 (m. 2H. H-3 and H-5). 3.78 (s. 3H. C-4-OC*H*_3_). 3.20–3.24 (m. 2H. H-α). 2.92–3.02 (m. 2H. H-β).

## 4. Conclusions

This study investigates the biotransformation of various 4′-hydroxychalcones using non-conventional yeast strains, highlighting their efficiency in producing dihydrochalcones. The findings reveal significant variability in the biotransformation capabilities of different yeast strains. Specifically, *Yarrowia lipolytica* KCh 71, *Rhodotorula rubra* KCh 4 and KCh 82, and *Rhodotorula glutinis* KCh 242 demonstrated high efficiency, achieving over 98% conversion within an hour for most substrates.

*Saccharomyces cerevisiae* KCh 464, however, displayed variable performance, with high conversion rates for some substrates but significantly lower rates for others. This variation underscores the influence of yeast strain specificity on the biotransformation process, indicating that strain selection is critical for optimizing production efficiency.

The position of methoxy groups on the chalcone ring significantly influenced the hydrogenation efficiency. For example, *trans*-4′-hydroxy-2-methoxychalcone (**2**) underwent slower transformation compared to other derivatives. This trend was consistent across various strains, suggesting that substituent position critically affects the biocatalytic activity.

This study’s results align with previous findings, where *Yarrowia lipolytica* KCh 71 strain was noted for its consistent performance regardless of methoxy group position.

A notable observation was the isomerization of *trans*-4′-hydroxy-chalcones to their *cis* isomers during the biotransformation process. This isomerization was significant in all transformations involving these substrates, highlighting a possible photochemical effect. The experiments indicated that light exposure, particularly sunlight, significantly catalyzed the isomerization, suggesting that ambient light conditions in the laboratory could influence the biotransformation outcomes.

This study employed a combination of TLC, GC, and NMR analyses to monitor and confirm the biotransformation products. The preparative-scale biotransformations yielded high-purity dihydrochalcones. The NMR analysis provided detailed insights into the structural characteristics of the synthesized compounds, confirming successful hydrogenation.

The high efficiency and specificity of the majority of yeast strains in transforming 4’-hydroxy-chalcones to dihydrochalcones underscore their potential for industrial applications. The production of 4’-hydroxy-dihydrochalcones, known for their pharmacological properties, can be scaled up for the development of therapeutic agents and nutraceuticals.

Future research should focus on further optimizing the biotransformation conditions, including exploring other non-conventional yeast strains and investigating the mechanisms underlying strain-specific activity. Additionally, addressing the isomerization issue through controlled light conditions can enhance the purity and yield of the desired biotransformation products.

Overall, this study provides a comprehensive framework for the efficient production of dihydrochalcones using biotechnological methods, paving the way for sustainable and scalable production of these valuable compounds.

## Figures and Tables

**Table 1:** 4’-Hydroxychalcones obtained via chemical synthesis and dihydrochalcones identified as a result of biotransformations.

*trans*-Chalcone	*cis*-Chalcone	Dihydrochalcones	R^1^	R^2^	R^3^
**1**	**1a**	**1b**	-H	-H	-H
**2**	**2a**	**2b**	-OCH_3_	-H	-H
**3**	**3a**	**3b**	-H	-OCH_3_	-H
**4**	**4a**	**4b**	-H	-H	-OCH_3_

**Table 2:** Conversion of *trans*-4’-hydroxy-chalcone (**1**) over time.

Strain	Composition [%]	Biotransformation Time					
		1 h	3 h	6 h	12 h	24 h	48 h
*Yarrowia lipolytica* KCh 71	**1**	1	<1	<1	<1	<1	<1
	**1a**	<1	<1	<1	<1	<1	<1
	**1b**	98	>99	>99	>99	>99	>99
*Saccharomyces cerevisiae* KCh 464	**1**	16	2	<1	<1	<1	<1
	**1a**	17	<1	<1	<1	<1	<1
	**1b**	67	97	>99	>99	>99	>99
*Rhodotorula rubra* KCh 82	**1**	2	1	<1	<1	<1	<1
	**1a**	<1	<1	<1	<1	<1	<1
	**1b**	98	99	>99	>99	>99	>99
*Rhodotorula rubra* KCh 4	**1**	1	<1	<1	<1	<1	<1
	**1a**	<1	<1	<1	<1	<1	<1
	**1b**	98	>99	>99	>99	>99	>99
*Rhodotorula glutinis* KCh 242	**1**	2	1	<1	<1	<1	<1
	**1a**	2	<1	<1	<1	<1	<1
	**1b**	96	99	>99	>99	>99	>99
*Rhodotorula marina* KCh 77	**1**	1	<1	<1	<1	<1	<1
	**1a**	<1	<1	<1	<1	<1	<1
	**1b**	98	>99	>99	>99	>99	>99

Table 2 shows the average conversion values (from three replicates). In none of the measurements did the error in determining the conversion exceed 5%. Exact mean values and standard deviations are provided in Supplementary Data—Table S1.

**Table 3:** Conversion of *trans*-4’-hydroxy-2-methoxychalcone (**2**) over time.

Strain	Composition [%]	Biotransformation Time					
		1 h	3 h	6 h	12 h	24 h	48 h
*Yarrowia lipolytica* KCh 71	**2**	10	9	9	7	6	4
	**2a**	12	7	5	4	3	3
	**2b**	78	84	86	89	91	93
*Saccharomyces cerevisiae* KCh 464	**2**	54	59	55	55	52	51
	**2a**	35	28	31	30	29	21
	**2b**	11	13	14	15	19	28
*Rhodotorula rubra* KCh 82	**2**	33	32	35	31	25	19
	**2a**	40	35	19	15	13	11
	**2b**	27	33	46	54	62	70
*Rhodotorula rubra* KCh 4	**2**	25	21	17	11	11	11
	**2a**	15	10	10	13	7	6
	**2b**	60	69	73	75	82	83
*Rhodotorula glutinis* KCh 242	**2**	13	9	7	7	5	5
	**2a**	13	3	2	2	3	3
	**2b**	74	88	91	91	92	92
*Rhodotorula marina* KCh 77	**2**	34	23	23	16	14	12
	**2a**	17	18	9	13	12	12
	**2b**	49	59	68	71	74	76

Table 3 shows the average conversion values (from three replicates). In none of the measurements did the error in determining the conversion exceed 7%. Exact mean values and standard deviations are provided in Supplementary Data—Table S2.

**Table 4:** Conversion of *trans*-4’-hydroxy-3-methoxychalcone (**3**) over time.

Strain	Composition [%]	Biotransformation Time					
		1 h	3 h	6 h	12 h	24 h	48 h
*Yarrowia lipolytica* KCh 71	**3**	1	1	<1	<1	<1	<1
	**3a**	<1	<1	<1	<1	<1	<1
	**3b**	98	99	>99	>99	>99	>99
*Saccharomyces cerevisiae* KCh 464	**3**	35	31	23	23	17	3
	**3a**	34	22	24	21	16	7
	**3b**	31	47	53	56	67	90
*Rhodotorula rubra* KCh 82	**3**	4	2	1	<1	<1	<1
	**3a**	2	<1	<1	<1	<1	<1
	**3b**	94	97	98	99	>99	>99
*Rhodotorula rubra* KCh 4	**3**	1	<1	<1	<1	<1	<1
	**3a**	<1	<1	<1	<1	<1	<1
	**3b**	99	99	>99	>99	>99	>99
*Rhodotorula glutinis* KCh 242	**3**	1	1	<1	<1	<1	<1
	**3a**	1	<1	<1	<1	<1	<1
	**3b**	97	98	>99	>99	>99	>99
*Rhodotorula marina* KCh 77	**3**	2	1	<1	<1	<1	<1
	**3a**	1	<1	<1	<1	<1	<1
	**3b**	97	98	99	>99	>99	>99

Table 4 shows the average conversion values (from three replicates). In none of the measurements did the error in determining the conversion exceed 5%. Exact mean values and standard deviations are provided in Supplementary Data—Table S3.

**Table 5:** Conversion of *trans*-4’-hydroxy-4-methoxychalcone (**4**) over time.

Strain	Composition [%]	Biotransformation Time					
		1 h	3 h	6 h	12 h	24 h	48 h
*Yarrowia lipolytica* KCh 71	**4**	7	3	1	<1	<1	<1
	**4a**	<1	3	<1	<1	<1	<1
	**4b**	92	93	98	99	>99	>99
*Saccharomyces cerevisiae* KCh 464	**4**	33	12	<1	<1	<1	<1
	**4a**	<1	4	6	4	2	2
	**4b**	67	84	93	95	97	98
*Rhodotorula rubra* KCh 82	**4**	10	1	<1	<1	<1	<1
	**4a**	<1	2	1	<1	<1	<1
	**4b**	89	97	98	>99	>99	>99
*Rhodotorula rubra* KCh 4	**4**	3	1	<1	<1	<1	<1
	**4a**	<1	<1	<1	<1	<1	<1
	**4b**	97	99	>99	>99	>99	>99
*Rhodotorula glutinis* KCh 242	**4**	25	5	2	2	1	<1
	**4a**	<1	3	1	<1	<1	<1
	**4b**	75	91	97	97	98	>99
*Rhodotorula marina* KCh 77	**4**	10	5	2	1	<1	<1
	**4a**	<1	2	1	<1	<1	<1
	**4b**	90	93	97	98	>99	>99

Table 5 shows the average conversion values (from three replicates). In none of the measurements did the error in determining the conversion exceed 5%. Exact mean values and standard deviations are provided in Supplementary Data—Table S4.

**Table 6:** Isomerization of trans-4’-hydroxy-2-methoxychalcone (**2**) in different organic solvents under different light sources [%].

Solvent	Isomerization Variant									
	A		B		C		D		E	
	*cis*	*trans*	*cis*	*trans*	*cis*	*trans*	*cis*	*trans*	*cis*	*trans*
ethanol	33.8	66.2	71.0	29.0	4.7	95.3	5.4	94.6	0	100
acetone	20.2	79.8	81.8	18.2	3.3	96.7	5.7	94.3	0	100
THF (tetrahydrofuran)	32.1	67.9	73.2	26.8	3.8	96.2	5.8	94.2	0	100
methylene chloride	25.1	74.9	79.9	20.1	4.4	95.6	7.3	92.6	0	100
ethyl acetate	23.0	77.0	82.4	17.6	2.5	97.5	4.4	95.6	0	100
DMSO (dimethyl sulfoxide)	28.6	71.4	80.3	19.7	2.4	97.6	4.6	95.4	0	100
methanol	26.0	74.0	80.6	19.4	3.1	96.9	5.7	94.3	0	100
acetonitrile	18.6	81.4	83.5	16.5	5.5	94.5	9.5	90.5	0	100

**A**—Diffused light (vials with substrate left on a laboratory bench); **B**—direct sunlight (vials with substrate exposed to direct sunlight); **C**—radiation 365 nm (vials with substrate placed under a lamp 365 nm); **D**—radiation 254 nm (vials with substrate placed under a laminar chamber lamp); **E**—no light (vials with substrate dissolved in various organic solvents wrapped in aluminum foil).

**Table 7:** Isomerization of the tested *trans*-chalcones dissolved in DMSO under sunlight.

Substrate	Duration of Exposure to Sunlight			
	30 min		1 h 30 min	
	*cis* [%]	*trans* [%]	*cis* [%]	*trans* [%]
**1**	84.4	15.6	82.1	17.9
**2**	82.3	17.7	82.1	17.9
**3**	82.9	17.1	82.7	17.3
**4**	35.6	64.4	38.7	61.3

**Table 8:** Summary of signals visible in the ^13^C NMR spectrum of the substrates and the biotransformation products.

Carbon		Compound											
	1 DMSO	1a DMSO	1b CDCl_3_	2 DMSO	2a DMSO	2b DMSO	3 DMSO	3a DMSO	3b DMSO	3b CDCl_3_	4 DMSO	4a DMSO	4b CDCl_3_
C=O	187.14	192.98	198.58	187.31	192.32	197.56	187.15	193.28	187.15	199.20	187.18	192.39	199.46
α	122.11	128.11	40.20	121.91	127.74	37.59	122.37	128.52	38.88	40.14	119.64	124.93	40.50
β	142.76	136.65	30.51	137.29	132.89	24.94	142.75	136.05	29.83	30.62	142.80	137.42	29.78
1	134.91	135.44	141.46	123.16	124.38	128.21	136.32	136.74	143.07	142.98	127.58	127.91	133.40
2	128.92	129.05	128.61	158.14	156.72	157.11	113.20	114.29	114.09	114.35	130.64	131.24	129.47
3	128.74	128.36	128.52	111.78	111.05	110.55	159.66	159.00	159.28	159.77	115.41	113.72	114.10
4	130.35	128.57	126.21	132.01	130.09	129.61	116.45	114.20	111.28	111.58	161.20	159.72	158.03
5	128.74	128.36	128.52	120.71	119.99	120.24	129.92	129.45	129.26	129.67	115.41	113.72	114.10
6	128.92	129.05	128.61	128.37	129.81	127.36	121.55	121.55	120.64	120.94	130.64	131.24	129.47
1’	129.12	128.28	129.64	129.26	128.57	129.01	129.10	128.24	128.24	129.58	129.41	128.72	130.97
2’.6’	131.24	131.43	130.79	131.12	131.18	130.45	131.27	131.42	130.51	130.95	131.12	131.30	130.96
3’.5’	115.41	115.56	115.52	115.42	115.36	115.22	115.40	115.58	115.23	115.63	114.46	115.50	115.63
4’	162.24	162.55	160.94	162.14	162.20	161.95	162.25	162.58	162.06	161.04	162.07	162.38	161.01
C-2-O*C*H_3_	-	-	-	55.72	55.32	55.25	-	-	-		-	-	-
C-3-O*C*H_3_	-	-	-	-	-	-	55.31	54.98	54.91	55.33	-	-	-
C-4-O*C*H_3_	-	-	-	-	-	-	-	-	-		55.43	55.22	55.45

**Table 9:** Compounds’ mass after extraction (preparative scale) [mg] in relation to 100 mg of the substrate used and retention times based on GC.

Retention Time [min]						Isolated Yield [%] of Dihydrochalcone
*trans*-Chalcone		*cis*-4’-Chalcone		Dihydrochalcone		
**1**	9.18	**1a**	8.57	**1b**	8.65	69%
**2**	10.12	**2a**	9.18	**2b**	9.29	23%
**3**	10.23	**3a**	9.30	**3b**	9.48	33%
**4**	10.51	**4a**	9.47	**4b**	9.59	79%

## Data Availability

Data are contained within this article and Supplementary Materials.

## Supplementary Materials

The following supporting information can be downloaded at https://www.mdpi.com/article/10.3390/ijms251910735/s1.
