# Supplementary material for: Efficient Production of 4’-Hydroxydihydrochalcones Using Non-Conventional Yeast Strains
Source: Int J Mol Sci. 2024 Oct 5;25(19):10735. doi: 10.3390/ijms251910735 (PMC11476679; doi:10.3390/ijms251910735)

Supplementary Data

Efficient Production of 4′-Hydroxydihydrochalcones Using Non-Conventional Yeast Strains

Paweł Chlipała *, Julia Bienia, Marcelina Mazur, Monika Dymarska, Tomasz Janeczko*

Department of Food Chemistry and Biocatalysis, Faculty of Biotechnology and Food Science, Wrocław University of Environmental and Life Sciences, 50-375 Wrocław, Poland. 125375@student.upwr.edu.pl (J.B.); monika.dymarska@upwr.edu.pl (M.D.)
Correspondence: pawel.chlipala@upwr.edu.pl (P.C.); tomasz.janeczko@upwr.edu.pl (T.J.)

Contents:

Table S1. Conversion of the *trans*-4′-hydroxy-chalcone (**1**) in time.
Table S2. Conversion of the *trans*-4′-hydroxy-2-methoxychalcone (**2**) in time.
Table S3. Conversion of the *trans*-4′-hydroxy-3-methoxychalcone (**3**) in time.
Table S4. Conversion of the *trans*-4′-hydroxy-4-methoxychalcone (**4**) in time.
Figure S1. The UV absorption maxima of *trans*- and *cis*-4′-hydroxy-chalkones.
Figure S2. ^{1}H NMR spectrum of *trans*-4′-hydroxychalcone (**1**) (DMSO-*d*$_6$, 600 MHz)
Figure S3. ^{13}C NMR spectrum of *trans*-4′-hydroxychalcone (**1**) (DMSO-*d*$_6$, 151 MHz)
Figure S4. COSY NMR spectrum of *trans*-4′-hydroxychalcone (**1**) (DMSO-*d*$_6$, 600 MHz)
Figure S5. HMQC NMR spectrum of *trans*-4′-hydroxychalcone (**1**) (DMSO-*d*$_6$, 600 MHz)
Figure S6. HMBC NMR spectrum of *trans*-4′-hydroxychalcone (**1**) (DMSO-*d*$_6$, 600 MHz)
Figure S7. ^{1}H NMR spectrum of *cis*-4′-hydroxychalcone (**1a**) (DMSO-*d*$_6$, 600 MHz)
Figure S8. ^{13}C NMR spectrum of *cis*-4′-hydroxychalcone (**1a**) (DMSO-*d*$_6$, 151 MHz)
Figure S9. COSY NMR spectrum of *cis*-4′-hydroxychalcone (**1a**) (DMSO-*d*$_6$, 600 MHz)
Figure S10. HMQC NMR spectrum of *cis*-4′-hydroxychalcone (**1a**) (DMSO-*d*$_6$, 600 MHz)
Figure S11. HMBC NMR spectrum of *cis*-4′-hydroxychalcone (**1a**) (DMSO-*d*$_6$, 600 MHz)
Figure S12. ^{1}H NMR spectrum of 4′-hydroxydihydrochalcone (**1b**) (DMSO-*d*$_6$, 600 MHz)
Figure S13. ^{13}C NMR spectrum of 4′-hydroxydihydrochalcone (**1b**) (DMSO-*d*$_6$, 151 MHz)
Figure S14. COSY NMR spectrum of 4′-hydroxydihydrochalcone (**1b**) (DMSO-*d*$_6$, 600 MHz)
Figure S15. HMQC NMR spectrum of 4′-hydroxydihydrochalcone (**1b**) (DMSO-*d*$_6$, 600 MHz)
Figure S16. HMBC NMR spectrum of 4′-hydroxydihydrochalcone (**1b**) (DMSO-*d*$_6$, 600 MHz)
Figure S17. ^{1}H NMR spectrum of *trans*-4′-hydroxy-2-methoxychalcone (**2**) (DMSO-*d*$_6$, 600 MHz)
Figure S18. Part of the ^{1}H NMR spectrum of *trans*-4′-hydroxy-2-methoxychalcone (**2**) (DMSO-*d*$_6$, 600 MHz)
Figure S19. ^{13}C NMR spectrum of *trans*-4′-hydroxy-2-methoxychalcone (**2**) (DMSO-*d*$_6$, 151 MHz)
Figure S20. COSY NMR spectrum of of *trans*-4′-hydroxy-2-methoxychalcone (**2**) (DMSO-*d*$_6$, 600 MHz)
Figure S21. HMQC NMR spectrum of of *trans*-4′-hydroxy-2-methoxychalcone (**2**) (DMSO-*d*$_6$, 600 MHz)
Figure S22. HMBC NMR spectrum of of *trans*-4′-hydroxy-2-methoxychalcone (**2**) (DMSO-*d*$_6$, 600 MHz)
Figure S22. ^{1}H NMR spectrum of *cis*-4′-hydroxy-2-methoxychalcone (**2a**) (DMSO-*d*$_6$, 600 MHz)
Figure S23. Part of the ^{1}H NMR spectrum of *cis*-4′-hydroxy-2-methoxychalcone (**2a**) (DMSO-*d*$_6$, 600 MHz)
Figure S24. ^{13}C NMR spectrum of *cis*-4′-hydroxy-2-methoxychalcone (**2a**) (DMSO-*d*$_6$, 151 MHz)
Figure S25. COSY NMR spectrum of of *cis*-4′-hydroxy-2-methoxychalcone (**2a**) (DMSO-*d*$_6$, 600 MHz)
Figure S26. HMQC NMR spectrum of of *cis*-4′-hydroxy-2-methoxychalcone (**2a**) (DMSO-*d*$_6$, 600 MHz)
Figure S27. HMBC NMR spectrum of of *cis*-4′-hydroxy-2-methoxychalcone (**2a**) (DMSO-*d*$_6$, 600 MHz)
Figure S28. ^{1}H NMR spectrum of 4′-hydroxy-2-methoxydihydrochalcone (**2b**) (DMSO-*d*$_6$, 600 MHz)
Figure S29. Part of the ^{1}H NMR spectrum of 4′-hydroxy-2-methoxydihydrochalcone (**2b**) (DMSO-*d*$_6$, 600 MHz)
Figure S30. ^{13}C NMR spectrum of 4′-hydroxy-2-methoxydihydrochalcone (**2b**) (DMSO-*d*$_6$, 151 MHz)
Figure S31. COSY NMR spectrum of of 4′-hydroxy-2-methoxydihydrochalcone (**2b**) (DMSO-*d*$_6$, 600 MHz)
Figure S32. HMQC NMR spectrum of of 4′-hydroxy-2-methoxydihydrochalcone (**2b**) (DMSO-*d*$_6$, 600 MHz)

Figure S33. HMBC NMR spectrum of of 4′-hydroxy-2-methoxydihydrochalcone (**2b**) (DMSO-d_6, 600 MHz)
Figure S34. ^{1}H NMR spectrum of *trans*-4′-hydroxy-3-methoxychalcone (**3**) (DMSO-d_6, 600 MHz)
Figure S35. Part of the ^{1}H NMR spectrum of *trans*-4′-hydroxy-3-methoxychalcone (**3**) (DMSO-d_6, 600 MHz)
Figure S36. ^{13}C NMR spectrum of *trans*-4′-hydroxy-3-methoxychalcone (**3**) (DMSO-d_6, 151 MHz)
Figure S37. COSY NMR spectrum of of *trans*-4′-hydroxy-3-methoxychalcone (**3**) (DMSO-d_6, 600 MHz)
Figure S38. HMQC NMR spectrum of of *trans*-4′-hydroxy-3-methoxychalcone (**3**) (DMSO-d_6, 600 MHz)
Figure S39. HMBC NMR spectrum of of *trans*-4′-hydroxy-3-methoxychalcone (**3**) (DMSO-d_6, 600 MHz)
Figure S40. ^{1}H NMR spectrum of *cis*-4′-hydroxy-3-methoxychalcone (**3a**) (DMSO-d_6, 600 MHz)
Figure S41. Part of the ^{1}H NMR spectrum of *cis*-4′-hydroxy-3-methoxychalcone (**3a**) (DMSO-d_6, 600 MHz)
Figure S42. ^{13}C NMR spectrum of *cis*-4′-hydroxy-3-methoxychalcone (**3a**) (DMSO-d_6, 151 MHz)
Figure S43. COSY NMR spectrum of of *cis*-4′-hydroxy-3-methoxychalcone (**3a**) (DMSO-d_6, 600 MHz)
Figure S44. HMQC NMR spectrum of of *cis*-4′-hydroxy-3-methoxychalcone (**3a**) (DMSO-d_6, 600 MHz)
Figure S45. HMBC NMR spectrum of of *cis*-4′-hydroxy-3-methoxychalcone (**3a**) (DMSO-d_6, 600 MHz)
Figure S46. ^{1}H NMR spectrum of 4′-hydroxy-3-methoxydihydrochalcone (**3b**) (DMSO-d_6, 600 MHz)
Figure S47. Part of the ^{1}H NMR spectrum of 4′-hydroxy-3-methoxydihydrochalcone (**3b**) (DMSO-d_6, 600 MHz)
Figure S48. Part of the ^{1}H NMR spectrum of 4′-hydroxy-3-methoxydihydrochalcone (**3b**) ($CDCl_3$, 600 MHz)
Figure S49. ^{13}C NMR spectrum of 4′-hydroxy-3-methoxydihydrochalcone (**3b**) (DMSO-d_6, 151 MHz)
Figure S50. ^{13}C NMR spectrum of 4′-hydroxy-3-methoxydihydrochalcone (**3b**) ($CDCl_3$, 151 MHz)
Figure S51. COSY NMR spectrum of of 4′-hydroxy-3-methoxydihydrochalcone (**3b**) (DMSO-d_6, 600 MHz)
Figure S52. HMQC NMR spectrum of of 4′-hydroxy-3-methoxydihydrochalcone (**3b**) (DMSO-d_6, 600 MHz)
Figure S53. HMBC NMR spectrum of of 4′-hydroxy-3-methoxydihydrochalcone (**3b**) (DMSO-d_6, 600 MHz)
Figure S54. ^{1}H NMR spectrum of *trans*-4′-hydroxy-4-methoxychalcone (**4**) (DMSO-d_6, 600 MHz)
Figure S55. Part of the ^{1}H NMR spectrum of *trans*-4′-hydroxy-4-methoxychalcone (**4**) (DMSO-d_6, 600 MHz)
Figure S56. ^{13}C NMR spectrum of *trans*-4′-hydroxy-4-methoxychalcone (**4**) (DMSO-d_6, 151 MHz)
Figure S57. COSY NMR spectrum of of *trans*-4′-hydroxy-4-methoxychalcone (**4**) (DMSO-d_6, 600 MHz)
Figure S58. HMQC NMR spectrum of of *trans*-4′-hydroxy-4-methoxychalcone (**4**) (DMSO-d_6, 600 MHz)
Figure S59. HMBC NMR spectrum of of *trans*-4′-hydroxy-4-methoxychalcone (**4**) (DMSO-d_6, 600 MHz)
Figure S60. ^{1}H NMR spectrum of *cis*-4′-hydroxy-4-methoxychalcone (**4a**) (DMSO-d_6, 600 MHz)
Figure S61. Part of the ^{1}H NMR spectrum of *cis*-4′-hydroxy-4-methoxychalcone (**4a**) (DMSO-d_6, 600 MHz)
Figure S62. ^{13}C NMR spectrum of *cis*-4′-hydroxy-4-methoxychalcone (**4a**) (DMSO-d_6, 151 MHz)
Figure S63. COSY NMR spectrum of of *cis*-4′-hydroxy-4-methoxychalcone (**4a**) (DMSO-d_6, 600 MHz)
Figure S64. HMQC NMR spectrum of of *cis*-4′-hydroxy-4-methoxychalcone (**4a**) (DMSO-d_6, 600 MHz)
Figure S65. HMBC NMR spectrum of of *cis*-4′-hydroxy-4-methoxychalcone (**4a**) (DMSO-d_6, 600 MHz)
Figure S66. ^{1}H NMR spectrum of 4′-hydroxy-4-methoxydihydrochalcone (**4b**) ($CDCl_3$, 600 MHz)
Figure S67. Part of the ^{1}H NMR spectrum of 4′-hydroxy-4-methoxydihydrochalcone (**4b**) ($CDCl_3$, 600 MHz)
Figure S68. ^{13}C NMR spectrum of 4′-hydroxy-4-methoxydihydrochalcone (**4b**) ($CDCl_3$, 151 MHz)
Figure S69. COSY NMR spectrum of of 4′-hydroxy-4-methoxydihydrochalcone (**4b**) ($CDCl_3$, 600 MHz)
Figure S70. HMQC NMR spectrum of of 4′-hydroxy-4-methoxydihydrochalcone (**4b**) ($CDCl_3$, 600 MHz)
Figure S71. HMBC NMR spectrum of of 4′-hydroxy-4-methoxydihydrochalcone (**4b**) ($CDCl_3$, 600 MHz)

Table S1. Conversion of the *trans*-4′-hydroxy-chalcone (**1**) in time.

Strain	Composition [%]	Biotransformation time					
		**1h**	**3h**	**6h**	**12h**	**24h**	**48h**
Yarrowia lipolytica KCh 71	**1**	1.14±0.19	0.46±0.51	0.36±0.05	0.36±0.25	0.35±0.20	0.13±0.06
	**1a**	0.50±0.71	0.10±0.15	0.24±0.34	0.16±0.11	0.08±0.04	0.00±0.00
	**1b**	98.36±0.52	99.41±0.79	99.40±0.86	99.48±0.36	99.57±0.24	99.87±0.06
Saccharomyces cerevisiae KCh 464	**1**	16.10±4.246	1.85±2.12	0.76±0.34	0.66±0.09	0.56±0.08	0.45±0.25
	**1a**	16.92±3.61	0.88±1.62	0.14±0.21	0.50±0.16	0.13±0.02	0.00±0.00
	**1b**	66.98±0.64	97.26±3.75	99.10±0.13	98.84±0.07	99.31±0.10	99.55±0.25
Rhodotorula rubra KCh 82	**1**	1.78±0.30	0.98±0.03	0.21±0.30	0.55±0.16	0.60±0.34	0.36±0.27
	**1a**	0.20±0.28	0.12±0.16	0.08±0.11	0.00±0.00	0.11±0.06	0.00±0.00
	**1b**	98.02±0.2	98.91±0.13	99.71±0.41	99.45±0.16	99.30±0.40	99.64±0.27
Rhodotorula rubra KCh 4	**1**	1.02±0.04	0.40±0.57	0.38±0.54	0.46±0.64	0.31±0.21	0.46±0.64
	**1a**	0.62±2.72	0.08±0.11	0.09±0.12	0.06±0.08	0.15±0.12	0.32±0.45
	**1b**	98.37±2.62	99.52±0.68	99.53±0.66	99.49±0.73	99.54±0.09	99.22±1.11
Rhodotorula glutinis KCh 242	**1**	2.21±0.30	1.28±0.40	0.22±0.32	0.12±0.18	0.09±0.13	0.00±0.00
	**1a**	1.86±0.21	0.13±0.18	0.14±0.20	0.14±0.20	0.37±0.52	0.00±0.00
	**1b**	95.94±0.09	98.59±0.58	99.64±0.52	99.74±0.37	99.54±0.65	100.00±0.00
Rhodotorula marina KCh 77	**1**	1.10±0.15	0.42±0.60	0.45±0.34	0.40±0.54	0.42±0.06	0.39±0.07
	**1a**	0.69±0.44	0.14±0.20	0.11±0.16	0.06±0.08	0.09±0.02	0.00±0.00
	**1b**	98.21±0.30	99.44±0.67	99.44±0.79	99.55±0.64	99.49±0.08	99.61±0.07

Table S2. Conversion of the *trans*-4′-hydroxy-2-methoxychalcone (**2**) in time.

Strain	Composition [%]	Biotransformation time					
		**1h**	**3h**	**6h**	**12h**	**24h**	**48h**
Yarrowia lipolytica KCh 71	**2**	10.09±2.47	8.76±0.70	9.04±0.12	7.24±0.70	5.95±0.50	4.19±0.54
	**2a**	12.06±1.74	7.21±2.29	5.32±0.91	4.25±4.53	3.40±4.40	2.67±1.94
	**2b**	77.86±4.21	84.05±3.00	85.65±1.03	89.51±5.23	90.65±4.90	93.14±2.48
Saccharomyces cerevisiae KCh 464	**2**	54.33±3.38	59.55±6.58	55.34±5.08	54.72±2.28	51.72±1.71	50.83±5.23
	**2a**	35.07±5.33	27.78±2.45	30.76±0.73	29.54±4.91	29.54±4.91	20.80±2.42
	**2b**	10.61±1.96	12.67±4.13	13.90±4.35	15.44±4.22	18.75±3.20	28.38±2.80
Rhodotorula rubra KCh 82	**2**	33.17±2.47	32.28±1.61	34.79±5.04	31.25±1.54	25.02±0.29	18.85±4.35
	**2a**	40.12±3.60	35.08±4.39	19.43±0.37	14.73±4.57	12.73±4.39	10.84±1.64
	**2b**	26.72±6.07	32.65±6.00	45.79±4.67	54.04±3.03	61.81±4.09	70.31±5.99
Rhodotorula rubra KCh 4	**2**	24.86±1.01	20.99±1.32	16.82±2.69	10.97±4.14	11.34±2.33	11.34±3.78
	**2a**	14.60±4.21	9.77±4.05	10.34±3.38	12.89±1.28	7.34±0.72	6.14±2.14
	**2b**	60.54±3.20	69.24±5.37	72.83±6.07	75.14±5.42	82.11±3.06	82.64±5.92
Rhodotorula glutinis KCh 242	**2**	12.86±1.45	9.00±1.04	6.91±0.95	7.13±2.30	4.89±1.10	5.26±1.68
	**2a**	13.08±0.75	3.20±1.95	2.22±1.22	2.26±1.60	3.00±0.87	2.70±1.94
	**2b**	74.07±0.65	87.81±2.99	90.88±2.17	90.61±3.90	92.11±1.97	92.04±0.29
Rhodotorula marina KCh 77	**2**	33.85±1.55	23.31±3.10	22.93±0.19	16.02±0.02	14.28±0.28	12.31±4.23
	**2a**	17.27±2.65	17.89±1.15	9.20±2.40	13.19±4.49	11.85±4.85	12.11±1.99
	**2b**	48.89±1.10	58.89±1.95	67.87±2.59	70.79±4.51	73.87±5.13	75.58±2.24

Table S3. Conversion of the *trans*-4′-hydroxy-3-methoxychalcone (**3**) in time.

Strain	Composition [%]	Biotransformation time					
		**1h**	**3h**	**6h**	**12h**	**24h**	**48h**
Yarrowia lipolytica KCh 71	**3**	1.09±0.07	0.66±0.36	0.32±0.26	0.42±0.39	0.38±0.04	0.21±0.07
	**3a**	0.79±0.26	0.29±0.15	0.28±0.17	0.32±0.15	0.31±0.06	0.31±0.08
	**3b**	98.13±0.21	99.05±0.51	99.40±0.43	99.26±0.54	99.31±0.11	99.48±0.16
Saccharomyces cerevisiae KCh 464	**3**	34.76±1.83	30.59±2.82	22.64±2.15	23.17±2.24	16.53±1.58	2.90±0.27
	**3a**	34.32±1.65	22.27±1.84	24.13±2.31	21.32±2.00	15.65±1.52	6.69±0.68
	**3b**	30.92±3.47	47.14±4.63	53.23±4.46	55.51±4.24	66.3.10	90.41±0.95
Rhodotorula rubra KCh 82	**3**	4.17±1.20	1.74±0.67	0.91±0.56	0.24±0.17	0.69±0.39	0.28±0.16
	**3a**	2.28±0.95	0.51±0.35	0.53±0.36	0.28±0.12	0.41±0.28	0.46±0.31
	**3b**	93.55±2.16	96.75±1.02	98.57±0.92	99.48±0.29	98.10±0.67	99.26±0.48
Rhodotorula rubra KCh 4	**3**	0.91±0.17	0.77±0.32	0.23±0.12	0.39±0.19	0.16±0.13	0.33±0.05
	**3a**	0.35±0.12	0.23±0.17	0.33±0.06	0.29±0.14	0.17±0.12	0.26±0.10
	**3b**	98.74±0.33	99.01±0.27	99.44±0.18	99.31±0.33	99.67±0.25	99.41±0.15
Rhodotorula glutinis KCh 242	**3**	1.46±0.44	1.17±0.15	0.58±0.16	0.51±0.29	0.44±0.28	0.38±0.17
	**3a**	1.14±0.45	0.55±0.44	0.55±0.10	0.67±0.42	0.51±0.29	0.49±0.35
	**3b**	97.40±0.89	98.28±0.30	98.87±0.26	98.72±0.71	99.05±0.57	99.13±0.18
Rhodotorula marina KCh 77	**3**	1.59±0.45	0.97±0.13	0.69±0.30	0.53±0.06	0.52±0.31	0.47±0.22
	**3a**	0.90±0.19	0.51±0.24	0.22±0.32	0.37±0.07	0.45±0.18	0.58±0.37
	**3b**	97.51±0.63	98.52±0.37	98.99±0.46	99.10±0.13	99.03±0.13	98.98±0.59

Table S4. Conversion of the *trans*-4′-hydroxy-4-methoxychalcone (**4**) in time.

**Strain**	**Composition [%]**	**Biotransformation time**					
		**1h**	**3h**	**6h**	**12h**	**24h**	**48h**
Yarrowia lipolytica KCh 71	**4**	7.07±4.22	3.46±1.69	1.45±0.20	0.68±0.36	0.29±0.17	0.42±0.11
	**4a**	0.40±0.56	3.23±1.73	0.33±0.06	0.57±0.42	0.58±0.38	0.48±0.41
	**4b**	92.53±3.67	93.31±3.42	98.22±0.26	98.75±0.75±	99.13±0.55	99.10±0.52
Saccharomyces cerevisiae KCh 464	**4**	32.78±3.60	11.70±4.33	0.85±0.47	0.76±0.54	0.60±0.33	0.40±0.33
	**4a**	0.63±0.46	3.63±2.31	6.27±2.55	3.77±0.79	2.53±1.32	1.57±0.84
	**4b**	66.59±4.06	83.67±4.64	92.88±3.02	95.47±1.33	96.87±1.65	98.03±1.17
Rhodotorula rubra KCh 82	**4**	10.08±3.82	0.97±1.09	0.55±0.36	0.42±0.03	0.49±0.22	0.31±0.05
	**4a**	0.64±0.12	2.25±0.36	1.44±0.38	0.57±0.55	0.54±0.24	0.59±0.44
	**4b**	89.28±4.92	96.78±1.45	98.01±0.74	99.01±0.58	98.97±0.46	99.10±0.39
Rhodotorula rubra KCh 4	**4**	2.76±0.88	0.81±0.14	0.80±0.05	0.35±0.31	0.36±0.05	0.37±0.33
	**4a**	0.26±0.16	0.47±0.16	0.41±0.40	0.39±0.12	0.29±0.11	0.47±0.29
	**4b**	96.99±0.72	98.72±0.30	98.78±0.46	99.25±0.43	99.35±0.06	99.17±0.62
Rhodotorula glutinis KCh 242	**4**	25.03±4.12	4.97±2.25	2.02±0.93	1.83±0.25	1.11±0.46	0.65±0.29
	**4a**	0.39±0.19	2.42±1.53	0.97±0.26	0.73±0.47	0.72±0.46	0.48±0.22
	**4b**	74.59±3.93	92.62±3.78	97.01±1.18	97.45±0.72	98.18±0.92	98.87±0.51
Rhodotorula marina KCh 77	**4**	10.08±0.30	4.89±1.21	1.99±0.42	0.96±0.17	0.59±0.34	0.55±0.17
	**4a**	0.35±0.24	2.55±0.52	0.92±0.56	0.67±0.41	0.45±0.07	0.35±0.19
	**4b**	89.57±	92.56±0.70	97.09±0.98	98.37±0.59	98.96±0.41	99.10±0.36

Figure S1. The UV absorption maxima of *trans*- and *cis*-4′-hydroxy-chalkones.

trans-4′-hydroxy-chalcone (**1**)

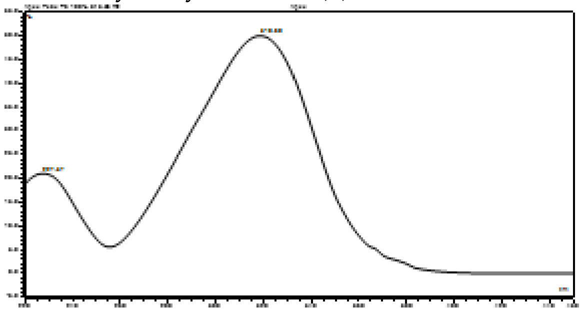

cis-4′-hydroxy-chalcone (**1a**)

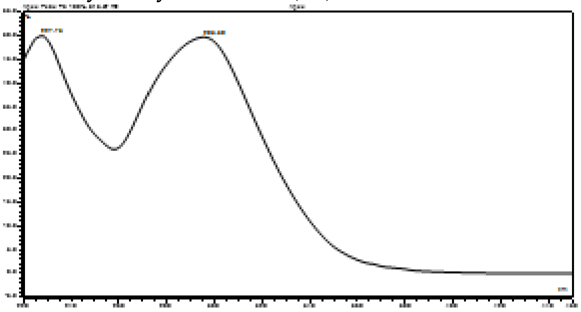

trans-4′-hydroxy-2-methoxychalcone (**2**)

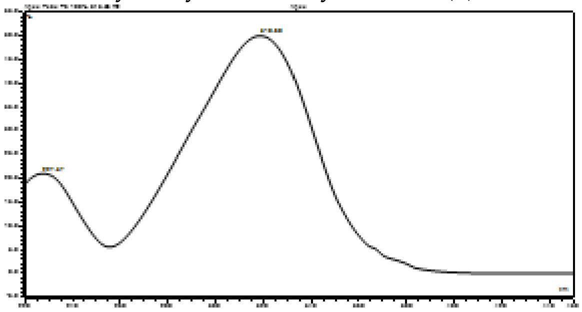

cis-4′-hydroxy-2-methoxychalcone (**2a**)

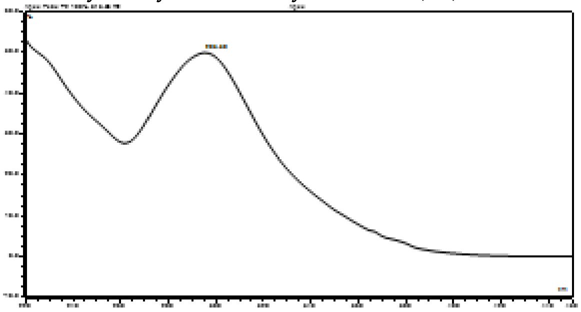

trans-4′-hydroxy-3-methoxychalcone (**3**)

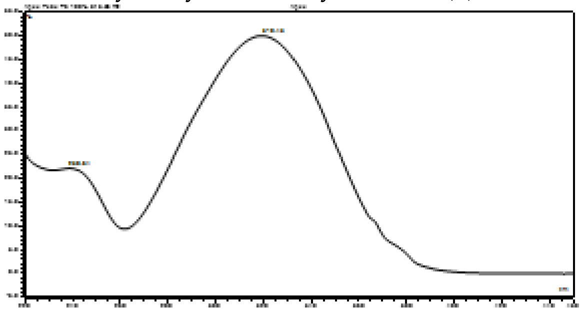

cis-4′-hydroxy-3-methoxychalcone (**3a**)

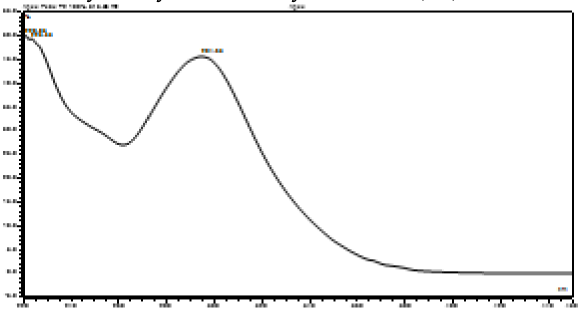

trans-4′-hydroxy-4-methoxychalcone (**4**)

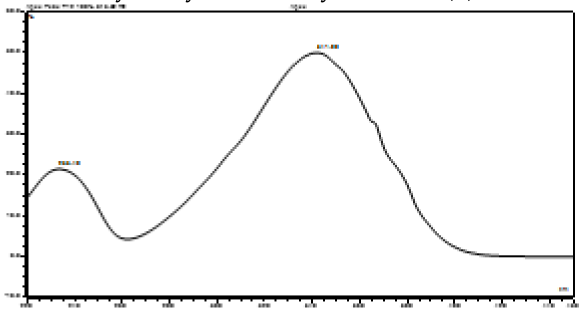

cis-4′-hydroxy-4-methoxychalcone (**4a**)

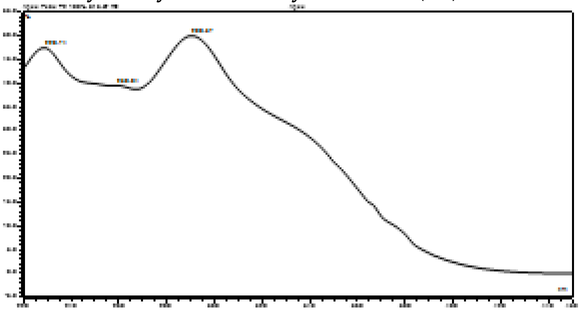

Figure S2. ^{1}H NMR spectrum of *trans*-4′-hydroxychalcone (**1**) (DMSO-d_6. 600 MHz)

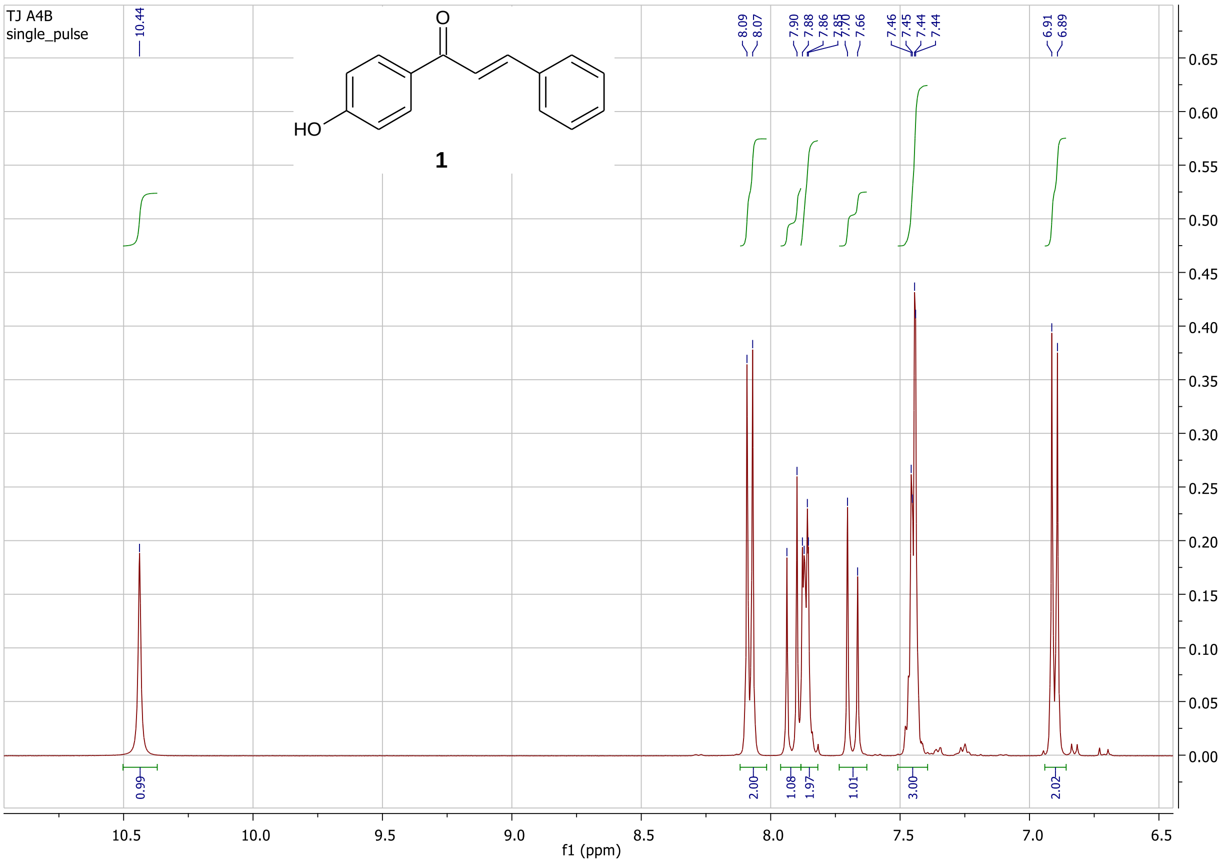

Figure S3. ^{13}C NMR spectrum of *trans*-4′-hydroxychalcone (**1**) (DMSO-d_6. 151 MHz)

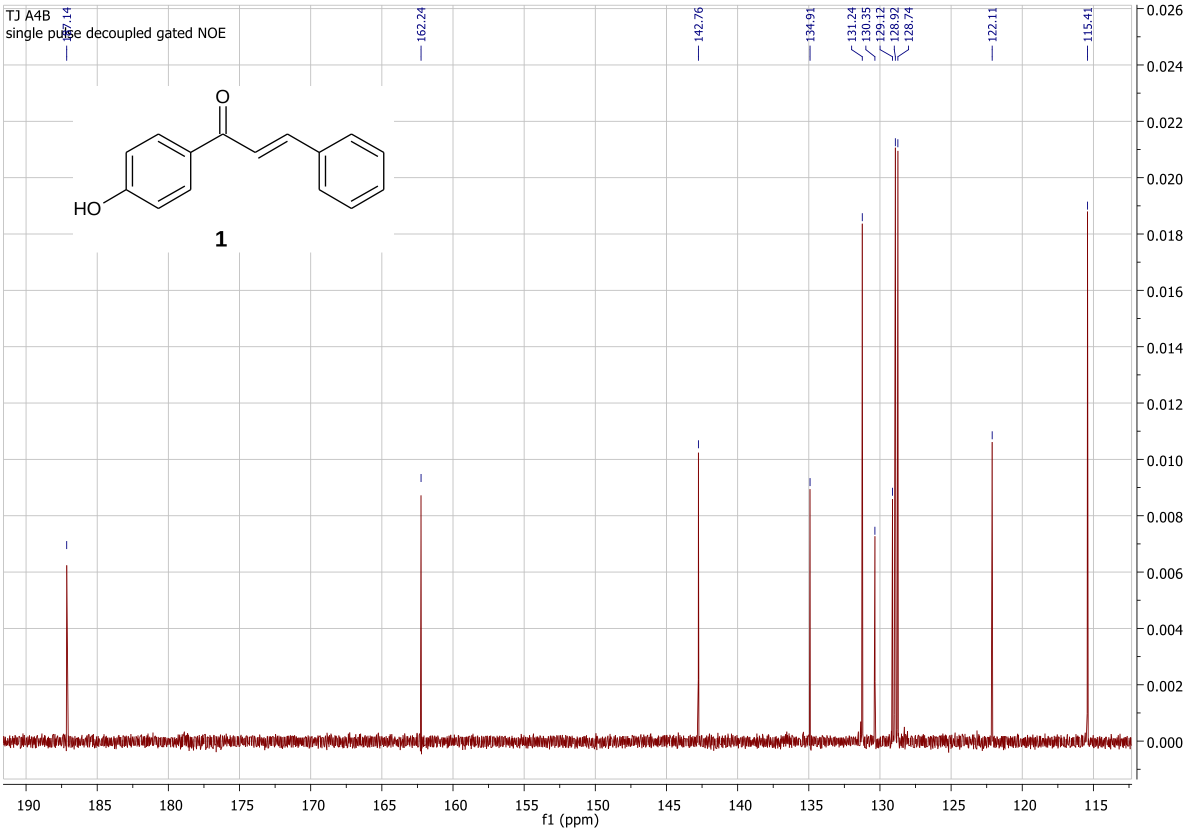

Figure S4. COSY NMR spectrum of *trans*-4′-hydroxychalcone (**1**) (DMSO-d_6. 600 MHz)

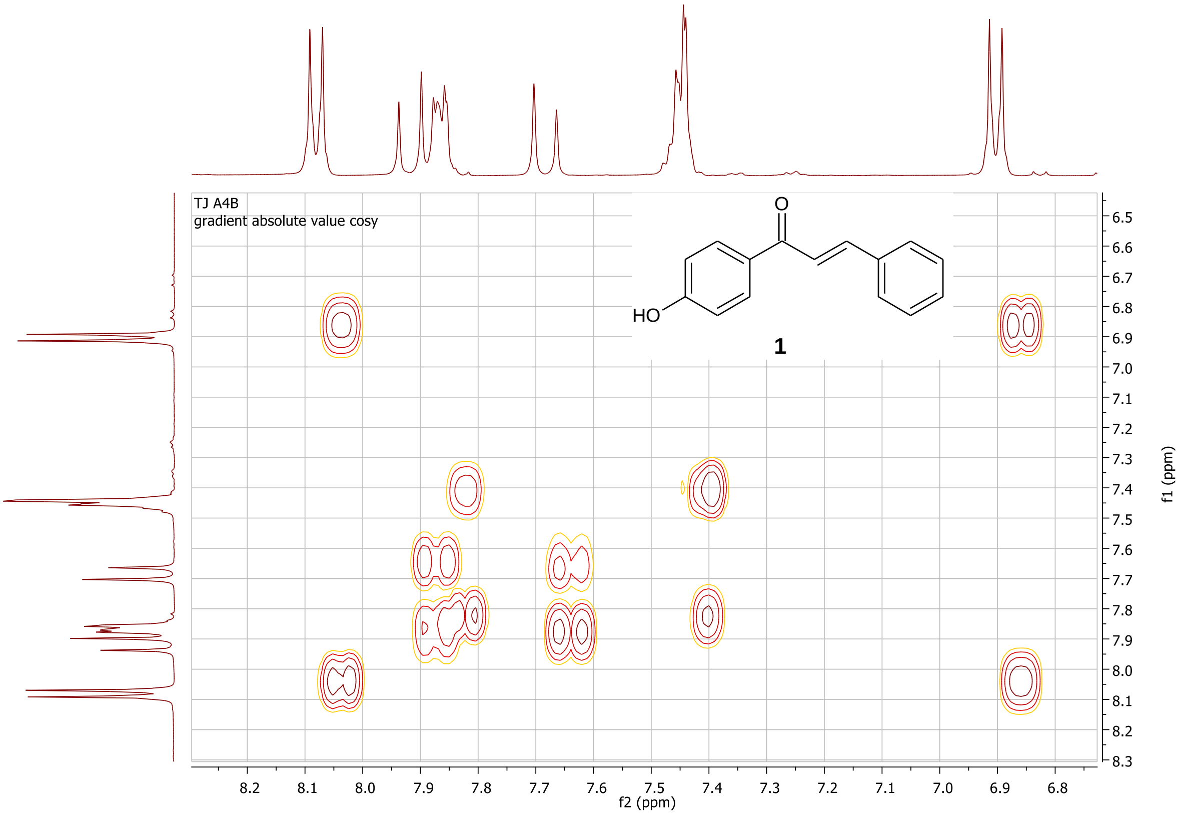

Figure S5. HMQC NMR spectrum of *trans*-4′-hydroxychalcone (**1**) (DMSO-d_6. 600 MHz)

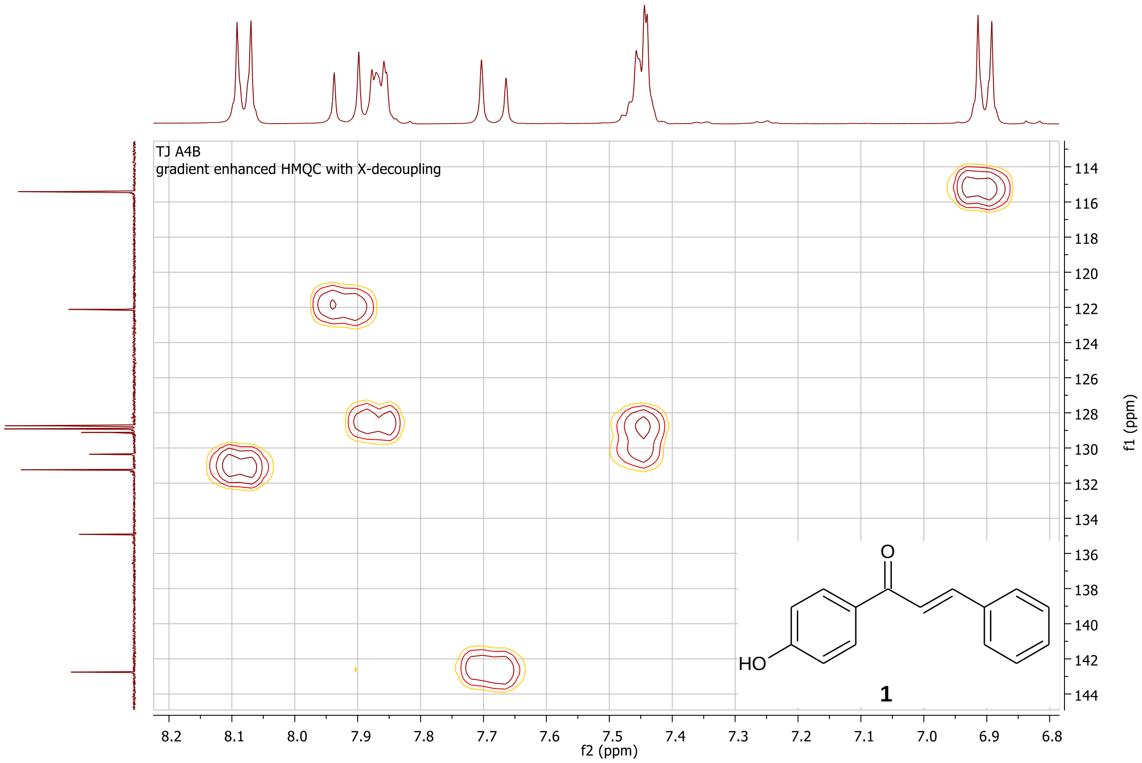

Figure S6. HMBC NMR spectrum of *trans*-4′-hydroxychalcone (**1**) (DMSO-d_6. 600 MHz)

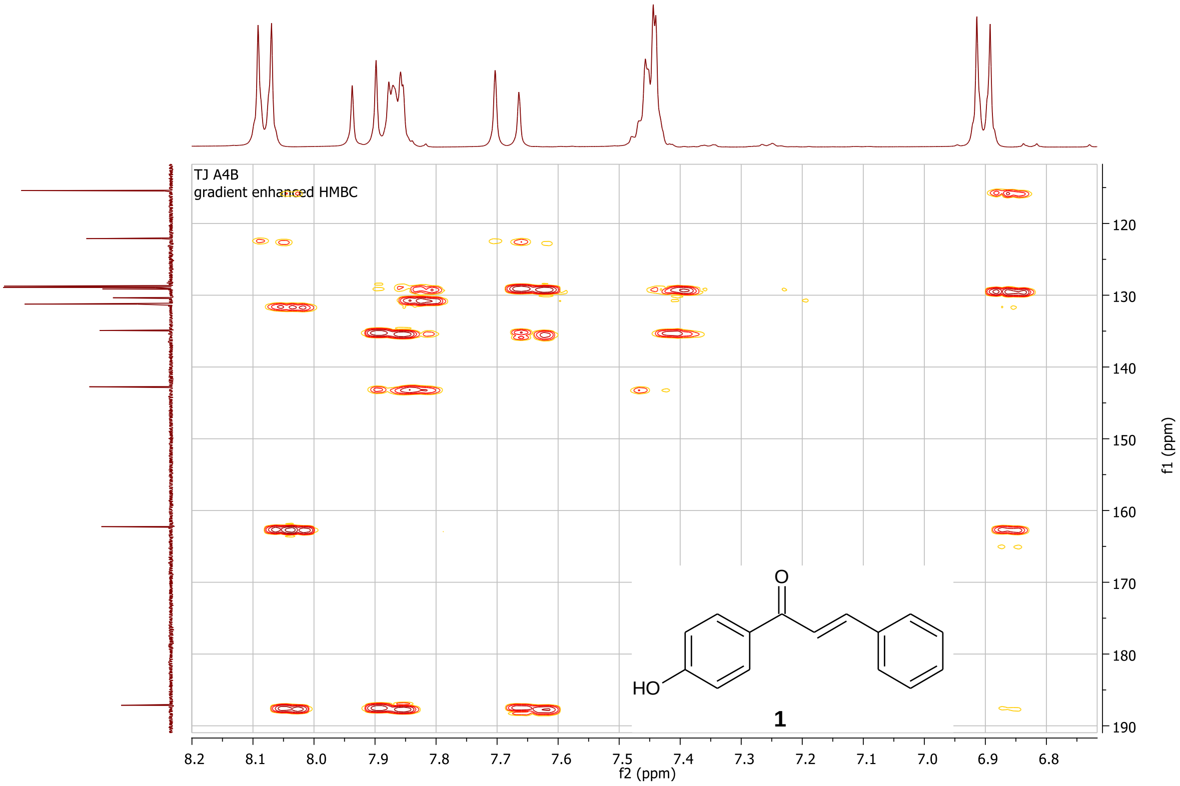

Figure S7. ^{1}H NMR spectrum of *cis*-4′-hydroxychalcone (**1a**) (DMSO-d_6. 600 MHz)

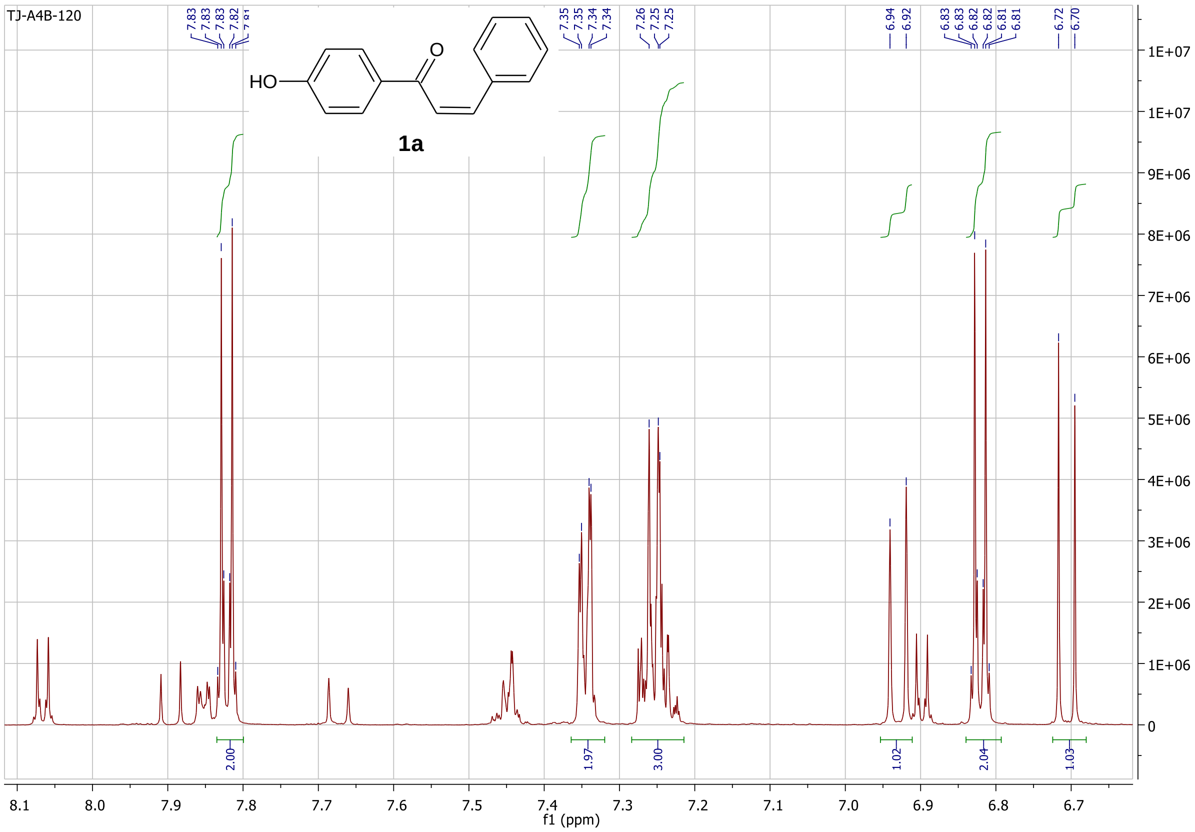

Figure S8. ^{13}C NMR spectrum of *cis*-4′-hydroxychalcone (**1a**) (DMSO-*d*6. 151 MHz)

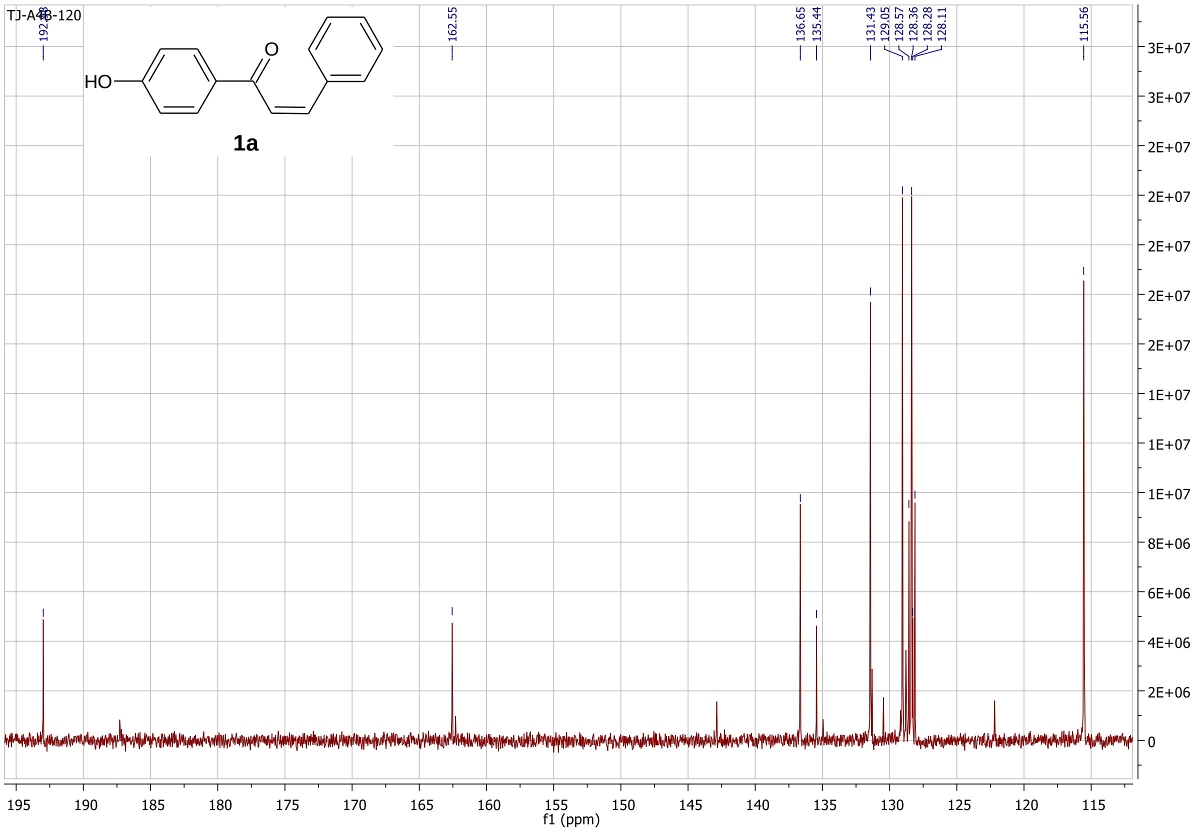

Figure S9. COSY NMR spectrum of *cis*-4′-hydroxychalcone (**1a**) (DMSO-*d*6. 600 MHz)

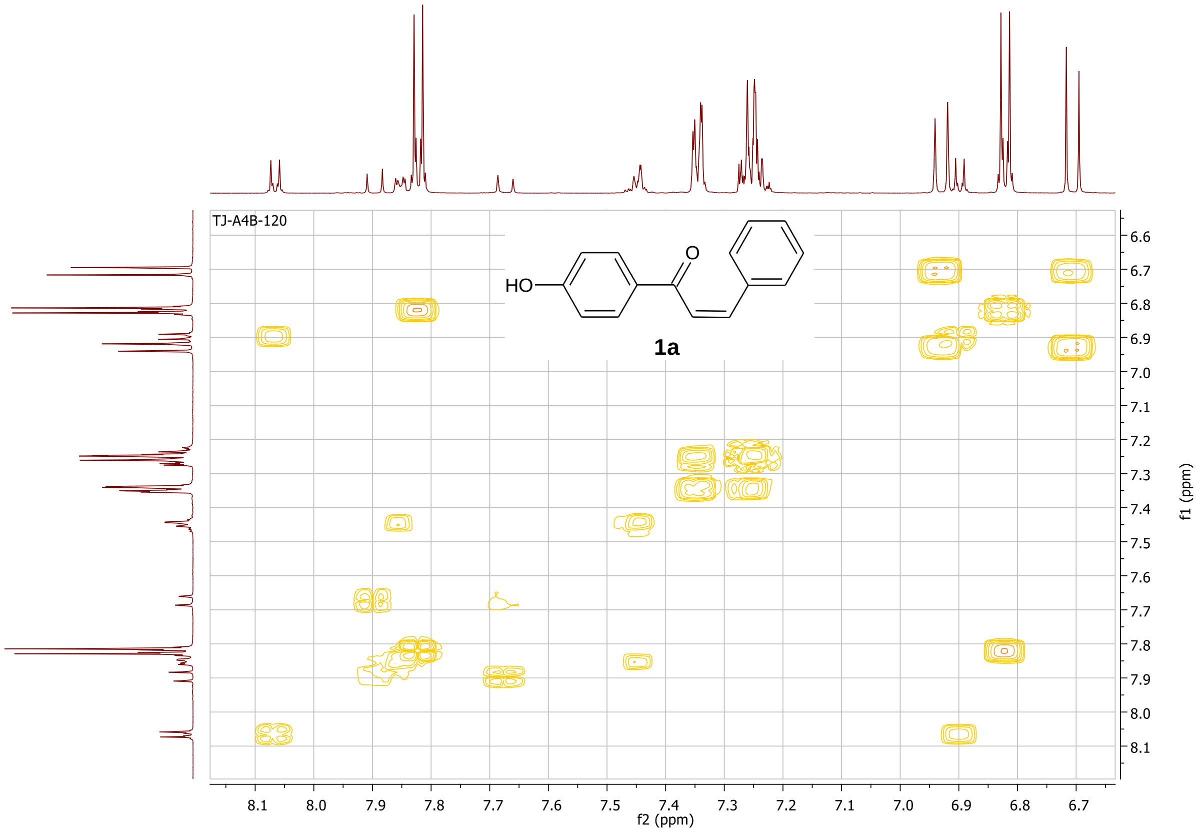

Figure S10. HMQC NMR spectrum of *cis*-4′-hydroxychalcone (**1a**) (DMSO-d_6. 600 MHz)

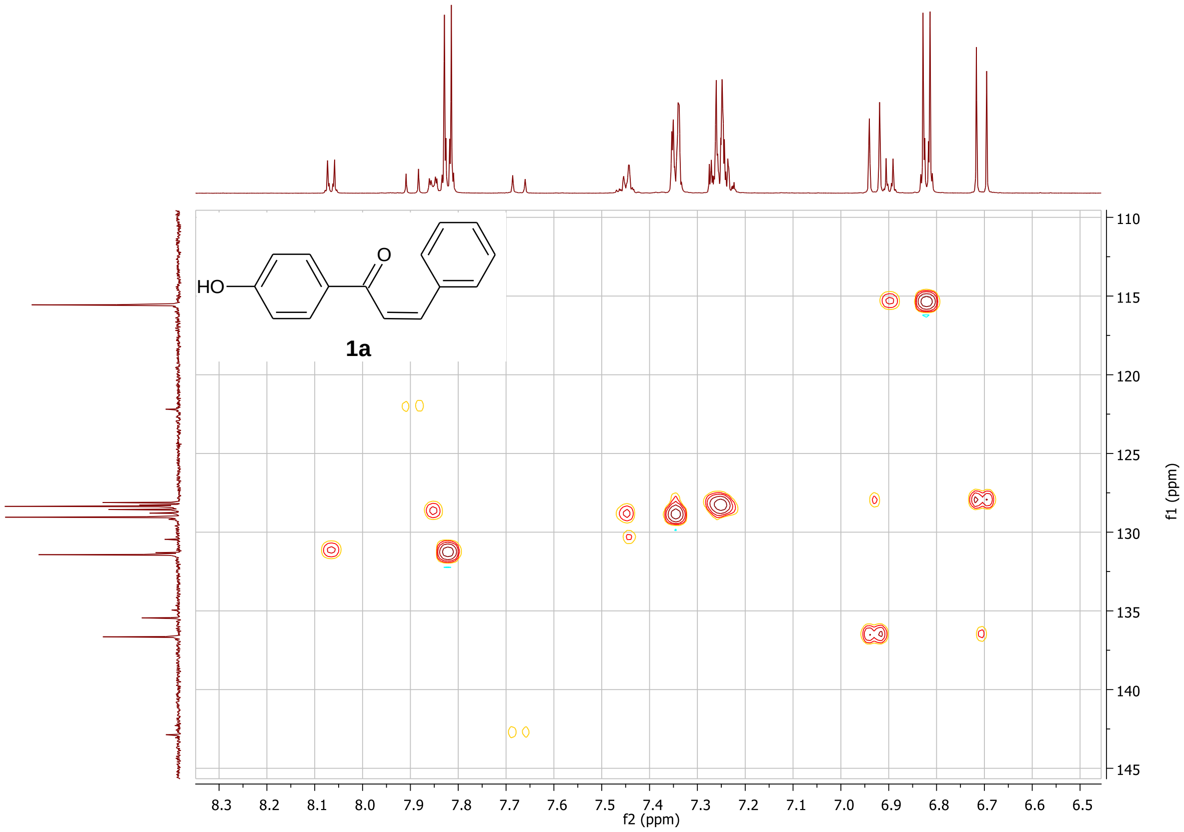

Figure S11. HMBC NMR spectrum of *cis*-4′-hydroxychalcone (**1a**) (DMSO-d_6. 600 MHz)

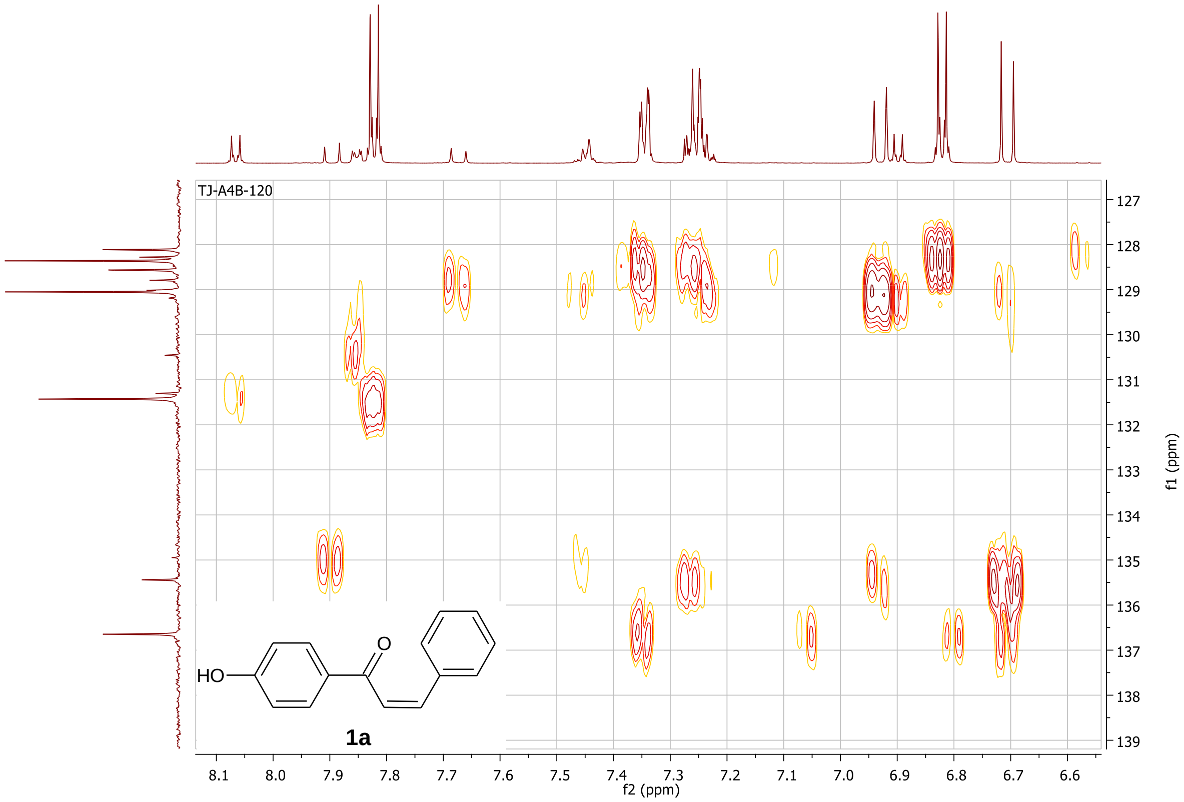

Figure S12. ^{1}H NMR spectrum of 4′-hydroxydihydrochalcone (**1b**) (DMSO-*d*6. 600 MHz)

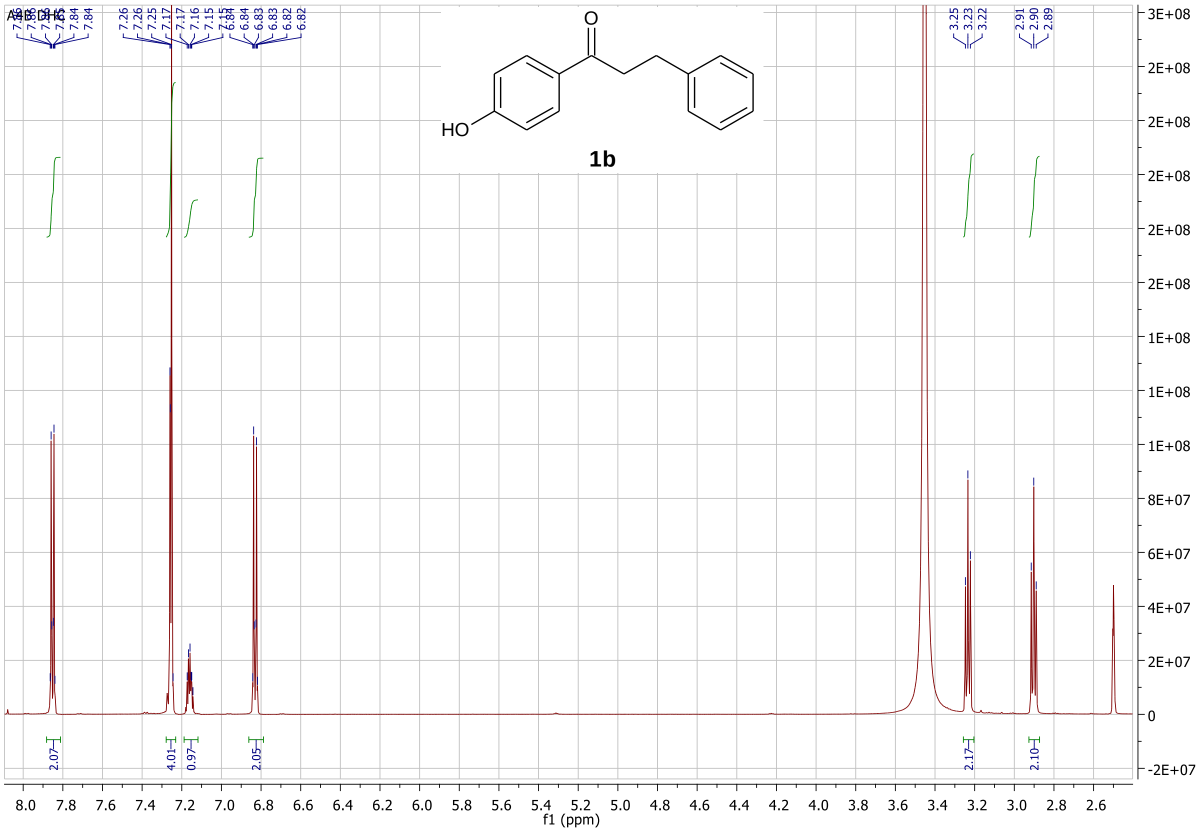

Figure S13. ^{13}C NMR spectrum of 4′-hydroxydihydrochalcone (**1b**) (DMSO-*d*6. 151 MHz)

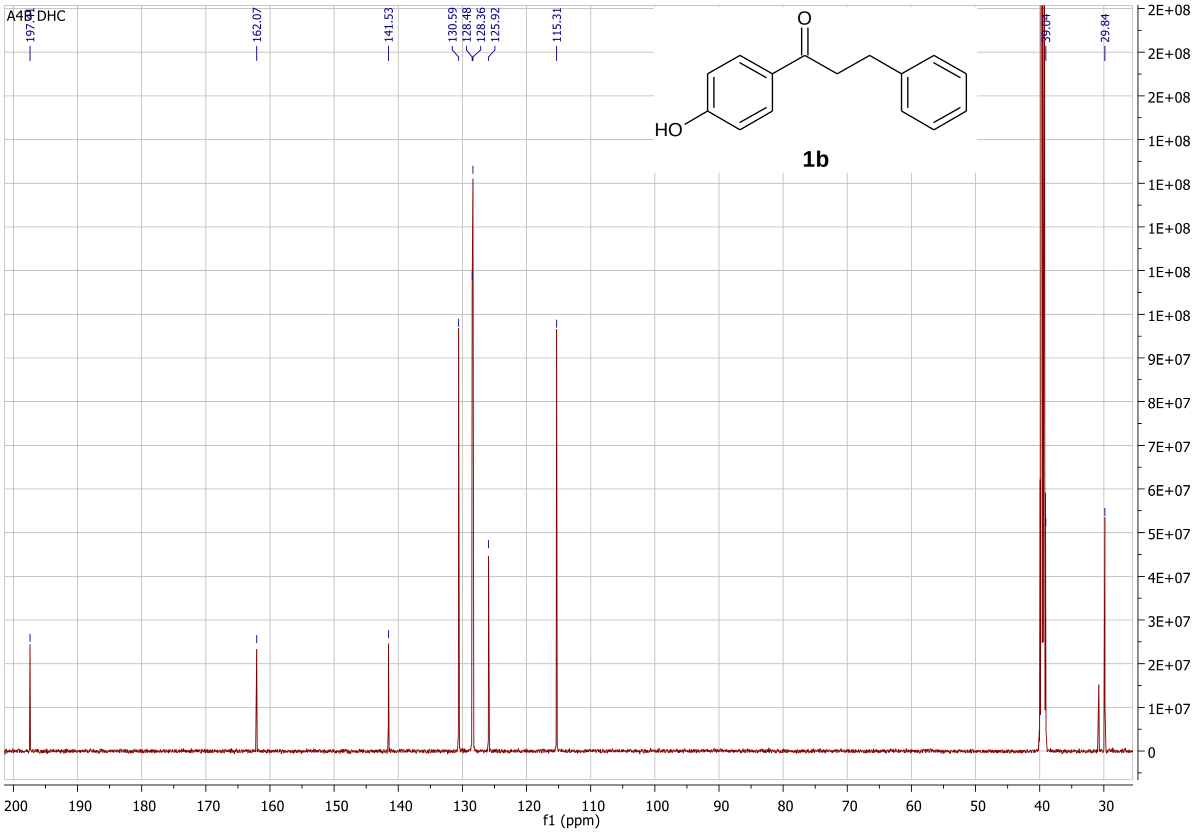

Figure S14. COSY NMR spectrum of 4′-hydroxydihydrochalcone (**1b**) (DMSO-d_6. 600 MHz)

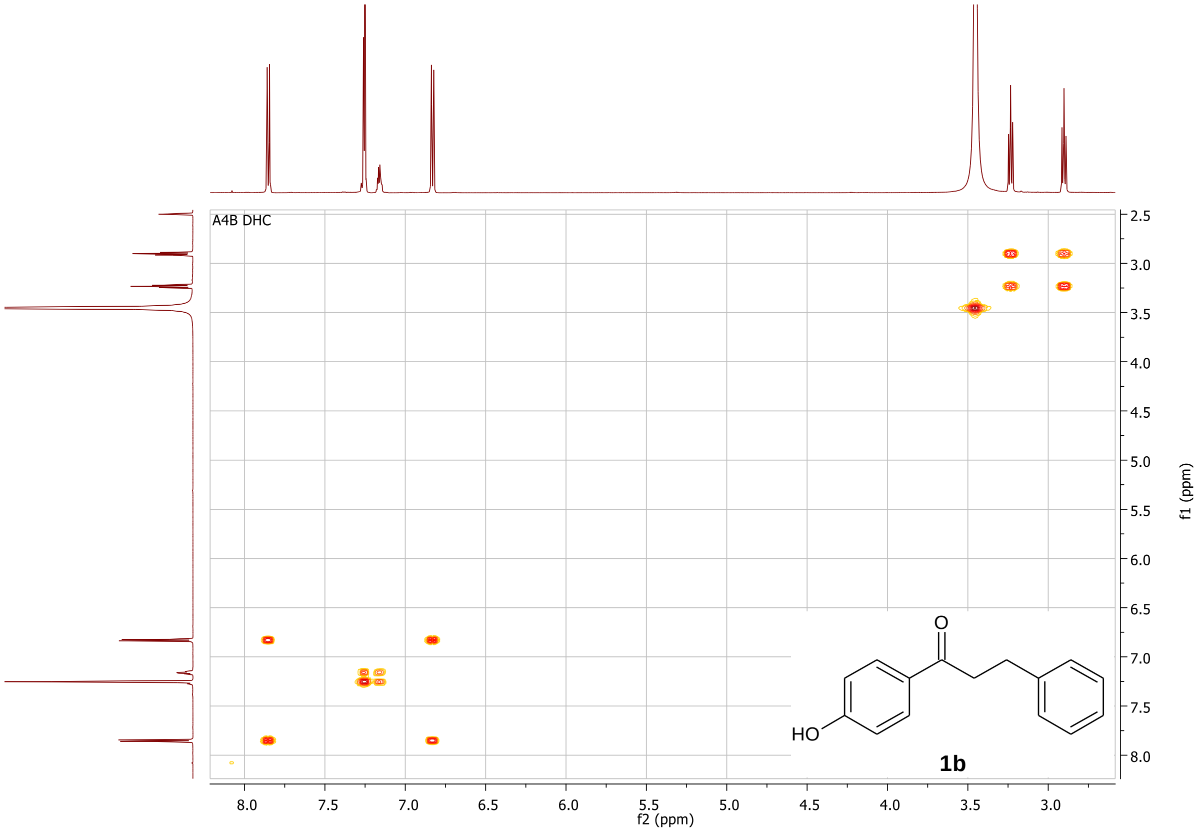

Figure S15. HMQC NMR spectrum of 4′-hydroxydihydrochalcone (**1b**) (DMSO-d_6. 600 MHz)

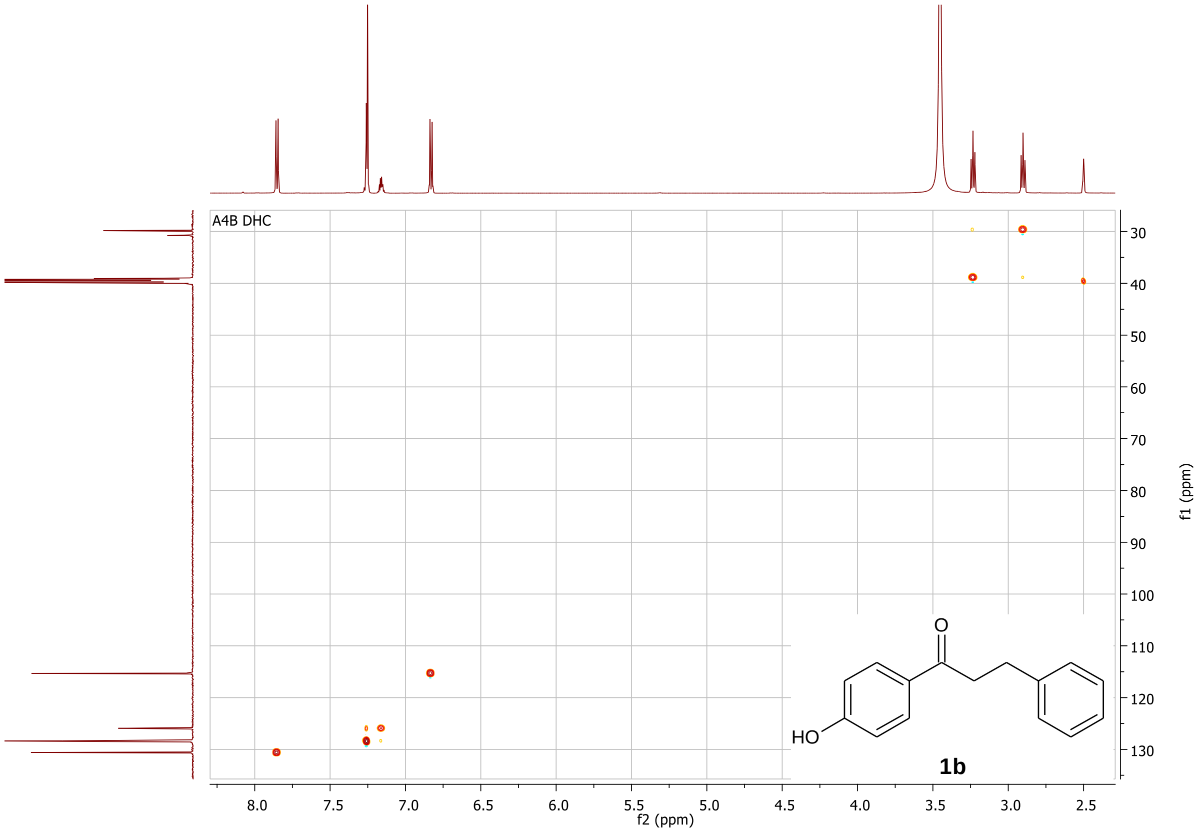

Figure S16. HMBC NMR spectrum of 4′-hydroxydihydrochalcone (**1b**) (DMSO-d_6. 600 MHz)

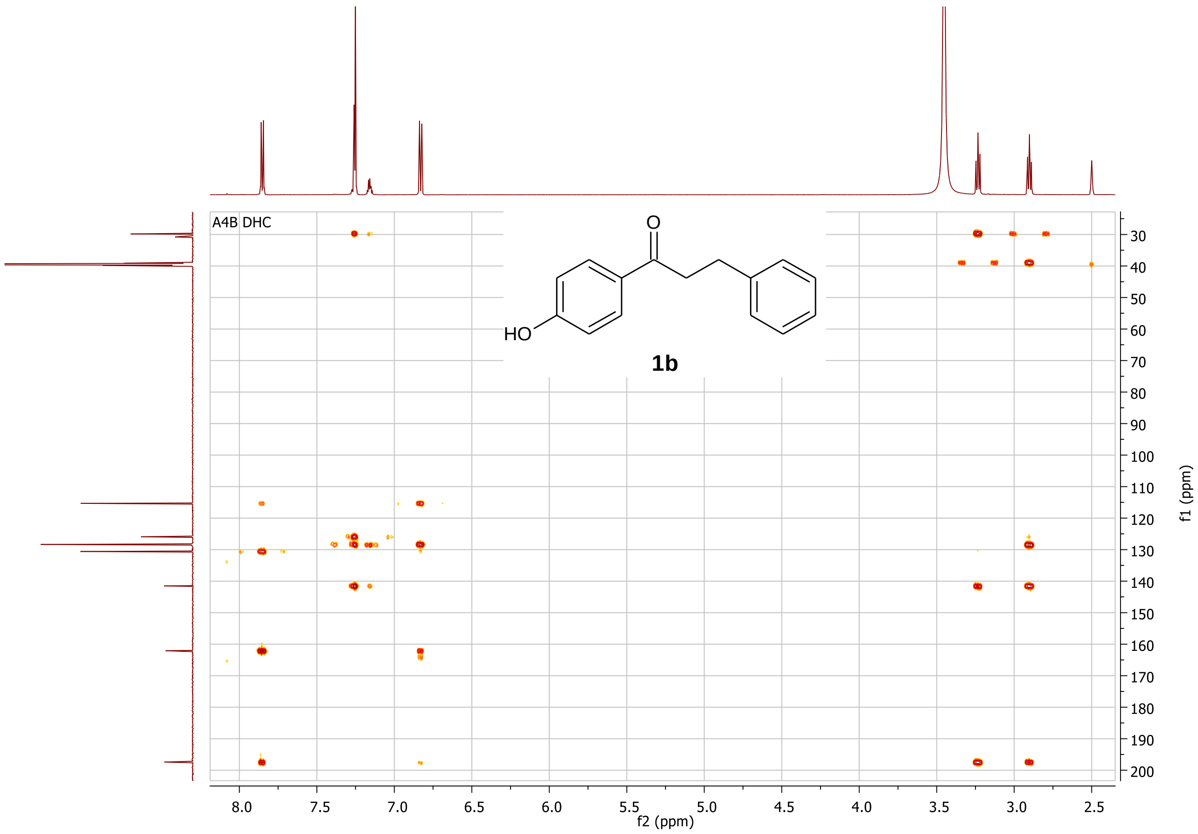

Figure S17. ^{1}H NMR spectrum of *trans*-4′-hydroxy-2-methoxychalcone (**2**) (DMSO-d_6. 600 MHz)

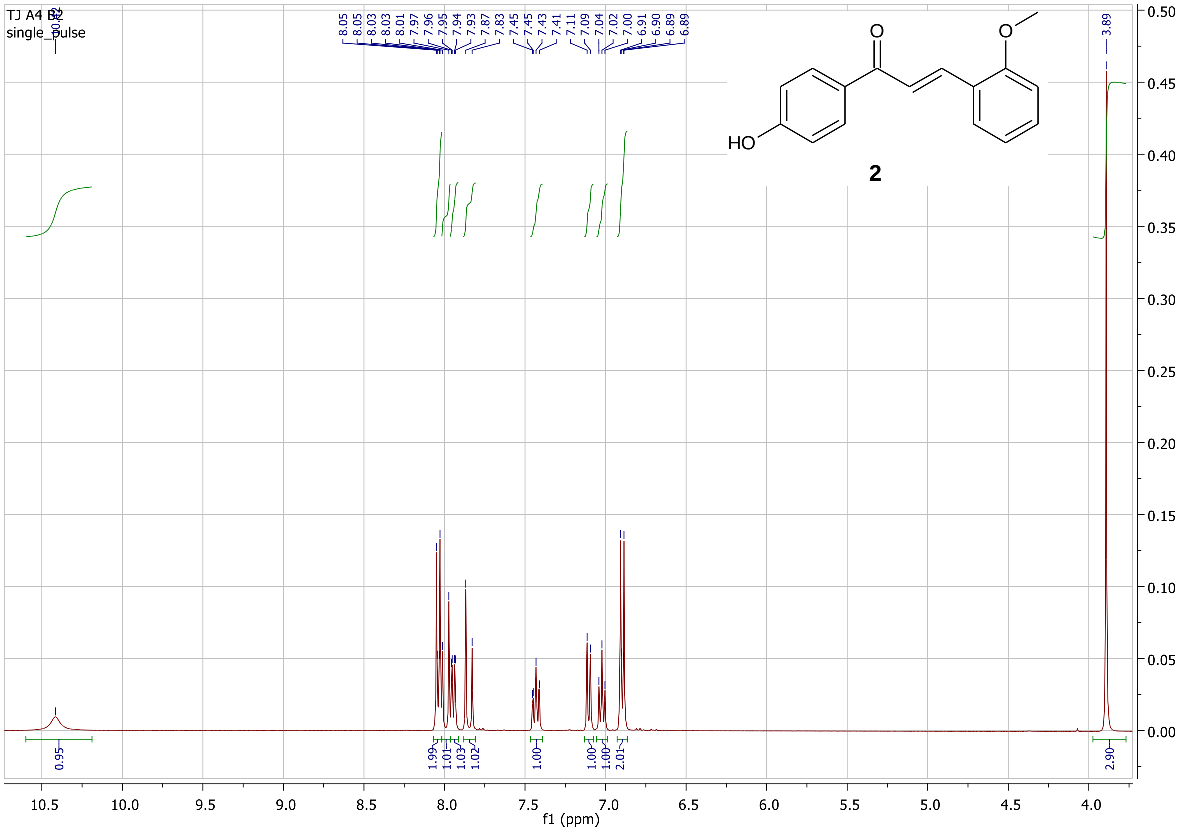

Figure S18. Part of the ^{1}H NMR spectrum of *trans*-4′-hydroxy-2-methoxychalcone (**2**) (DMSO-*d*$_6$. 600 MHz)

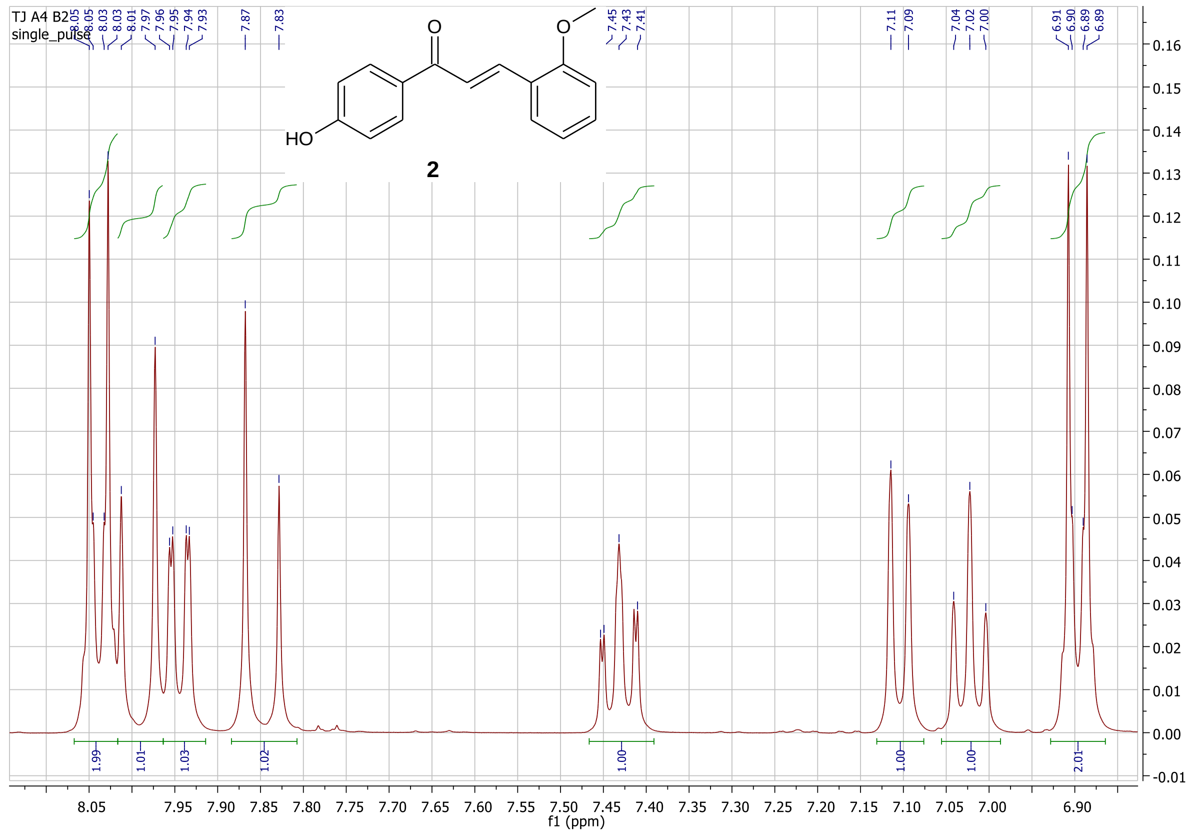

Figure S19. ^{13}C NMR spectrum of *trans*-4′-hydroxy-2-methoxychalcone (**2**) (DMSO-*d*$_6$. 151 MHz)

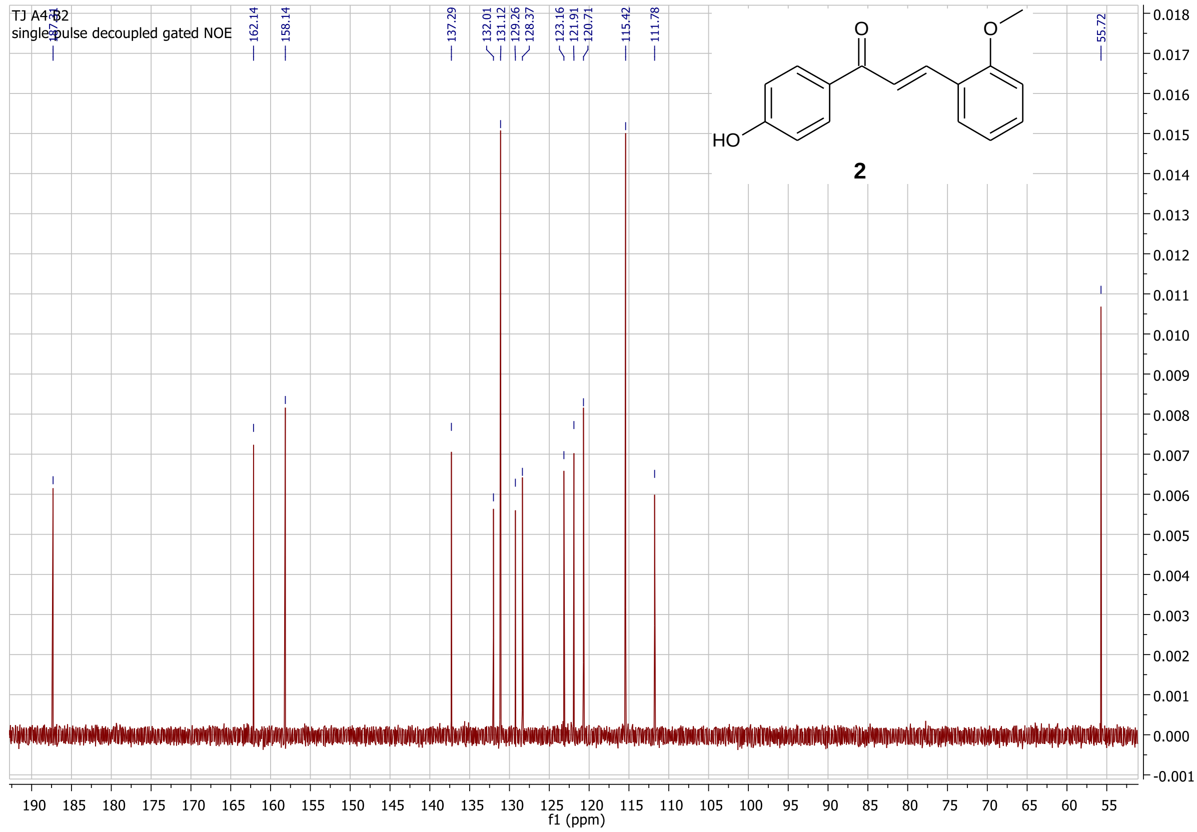

Figure S20. COSY NMR spectrum of of *trans*-4′-hydroxy-2-methoxychalcone (**2**) (DMSO-d_6. 600 MHz)

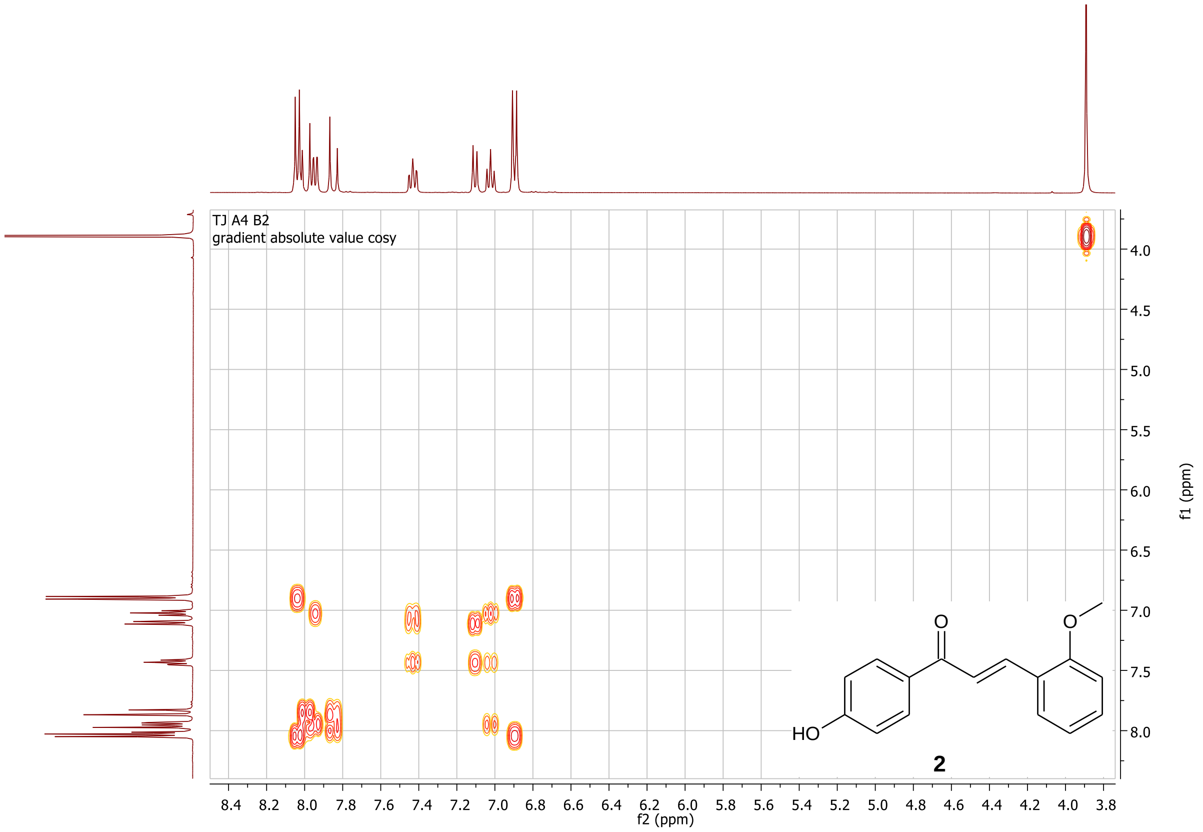

Figure S21. HMQC NMR spectrum of of *trans*-4′-hydroxy-2-methoxychalcone (**2**) (DMSO-d_6. 600 MHz)

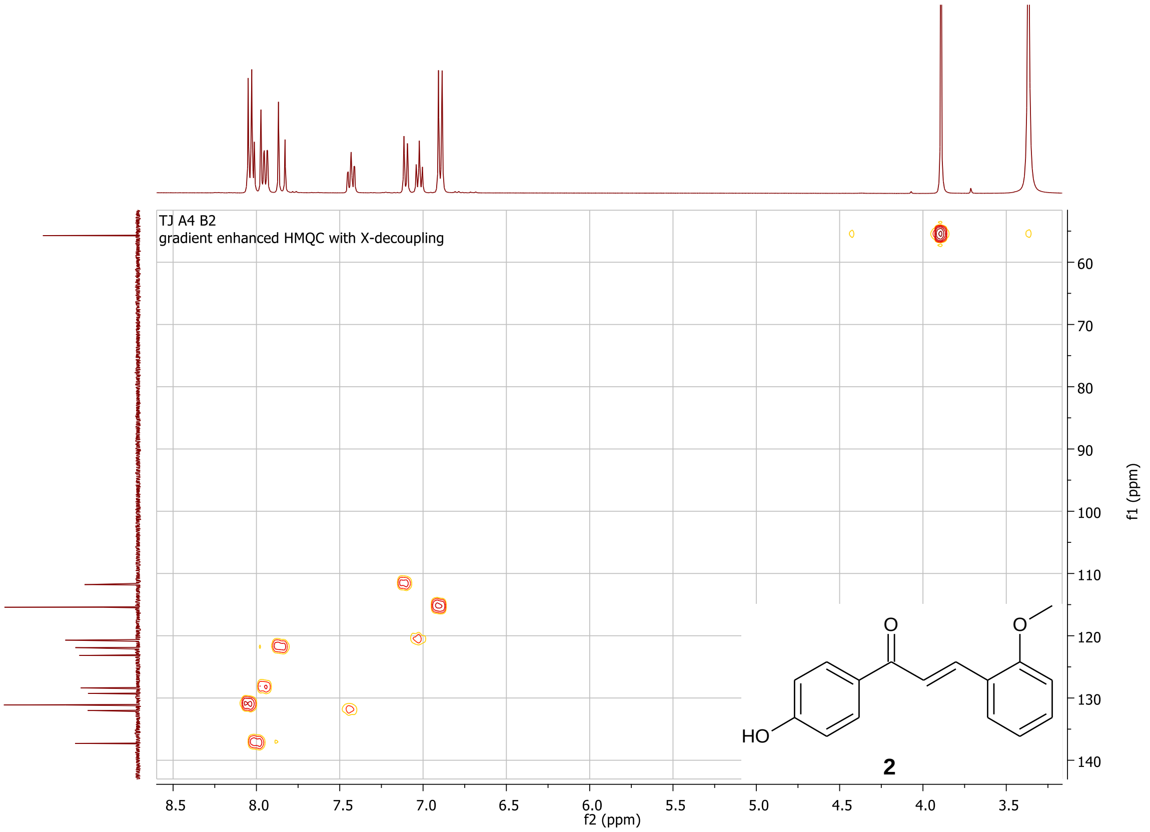

Figure S22. HMBC NMR spectrum of of *trans*-4′-hydroxy-2-methoxychalcone (**2**) (DMSO-d_6. 600 MHz)

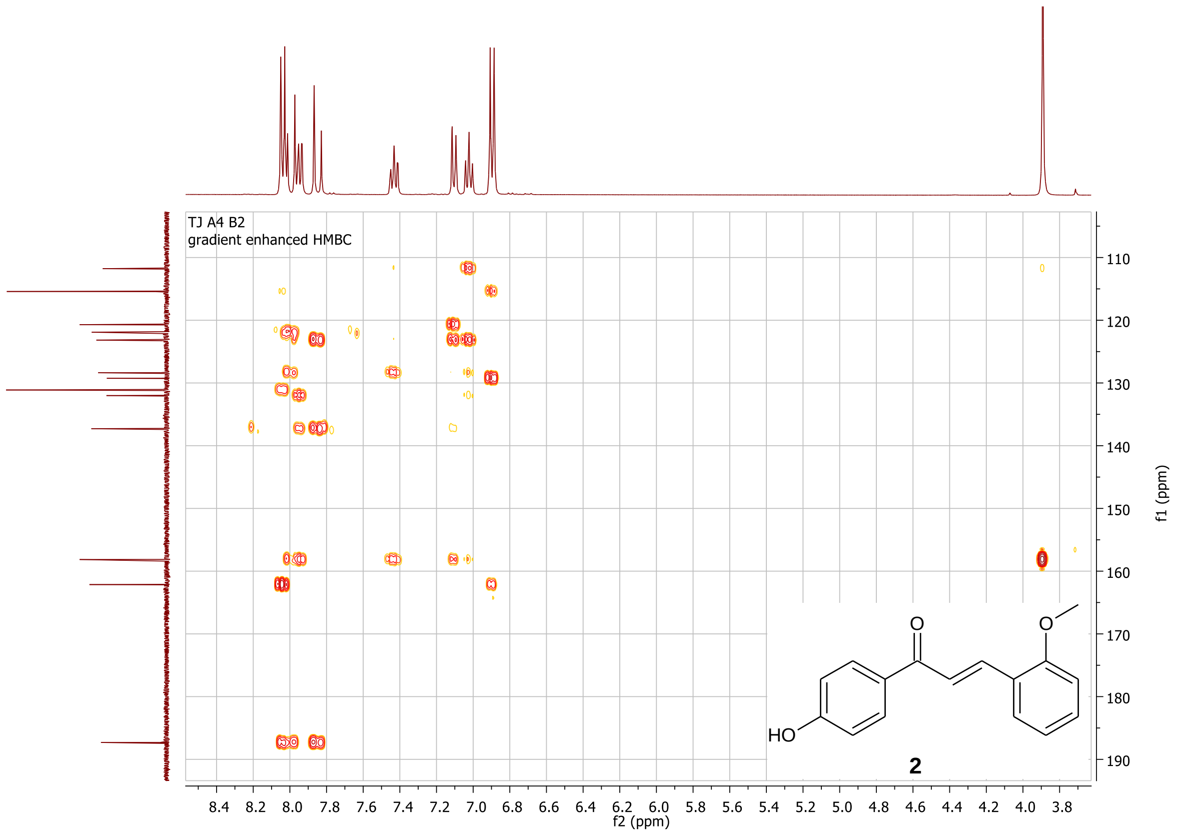

Figure S22. ^{1}H NMR spectrum of *cis*-4′-hydroxy-2-methoxychalcone (**2a**) (DMSO-d_6. 600 MHz)

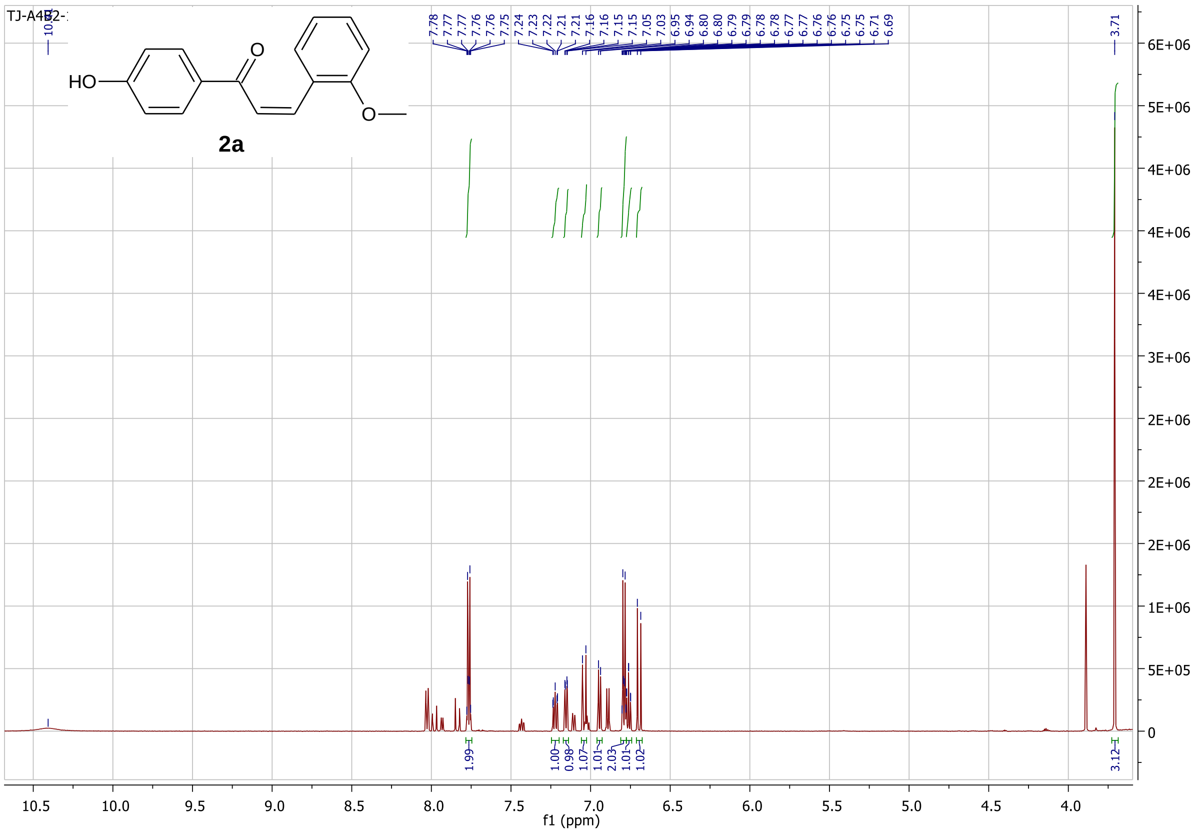

Figure S23. Part of the ^{1}H NMR spectrum of *cis*-4′-hydroxy-2-methoxychalcone (**2a**) (DMSO-d_6. 600 MHz)

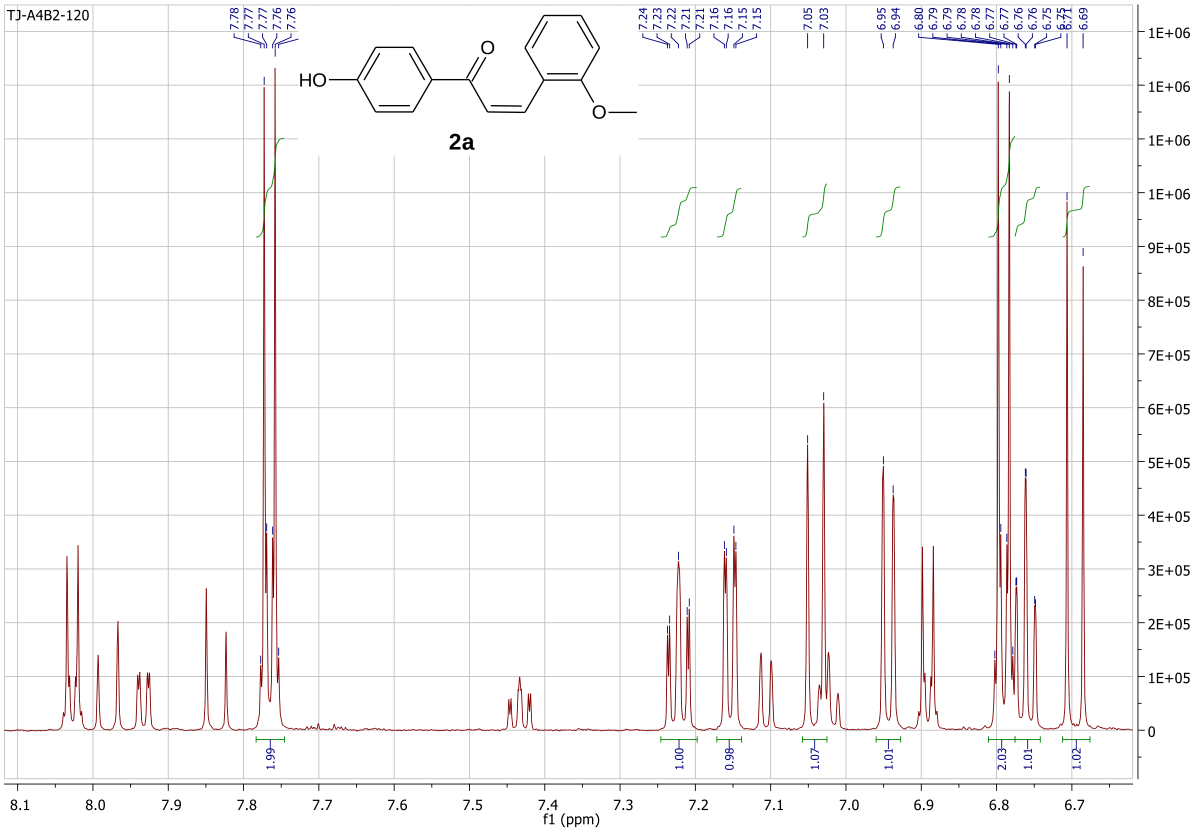

Figure S24. ^{13}C NMR spectrum of *cis*-4′-hydroxy-2-methoxychalcone (**2a**) (DMSO-d_6. 151 MHz)

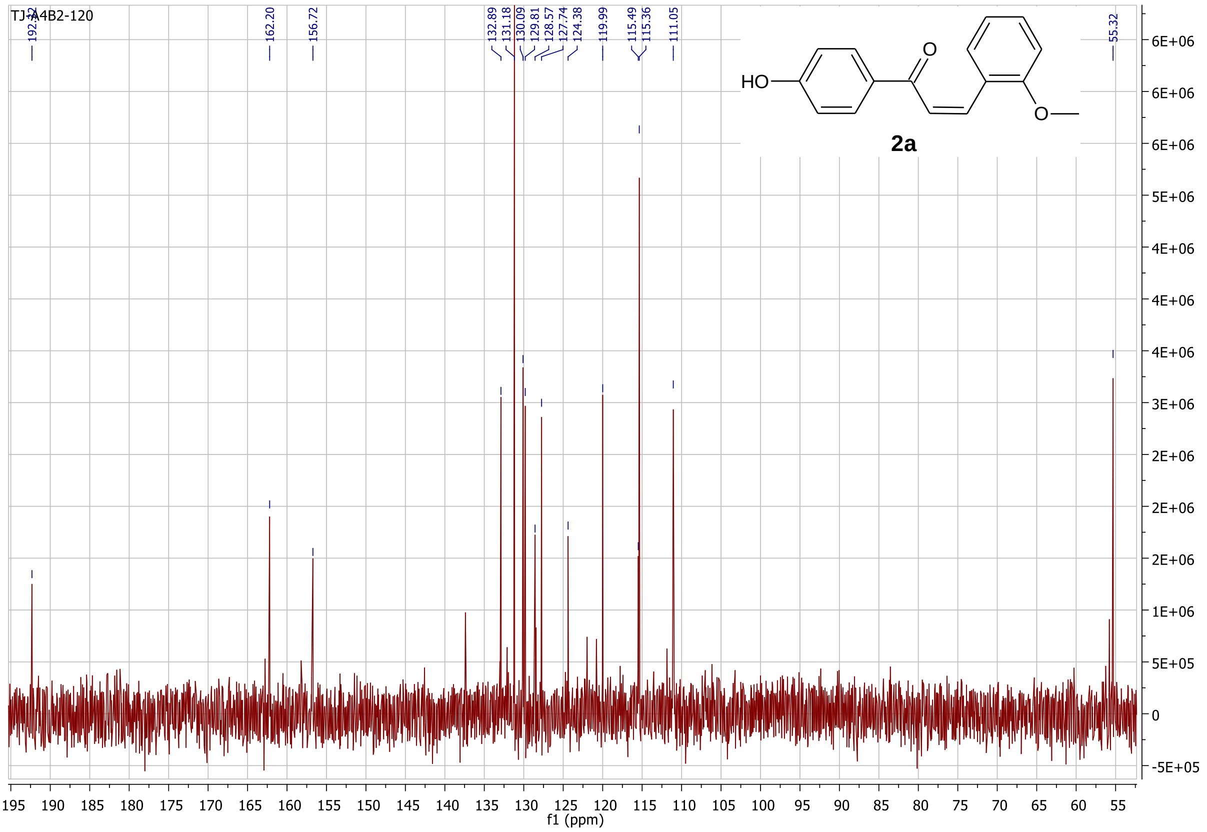

Figure S25. COSY NMR spectrum of of *cis*-4′-hydroxy-2-methoxychalcone (**2a**) (DMSO-d_6. 600 MHz)

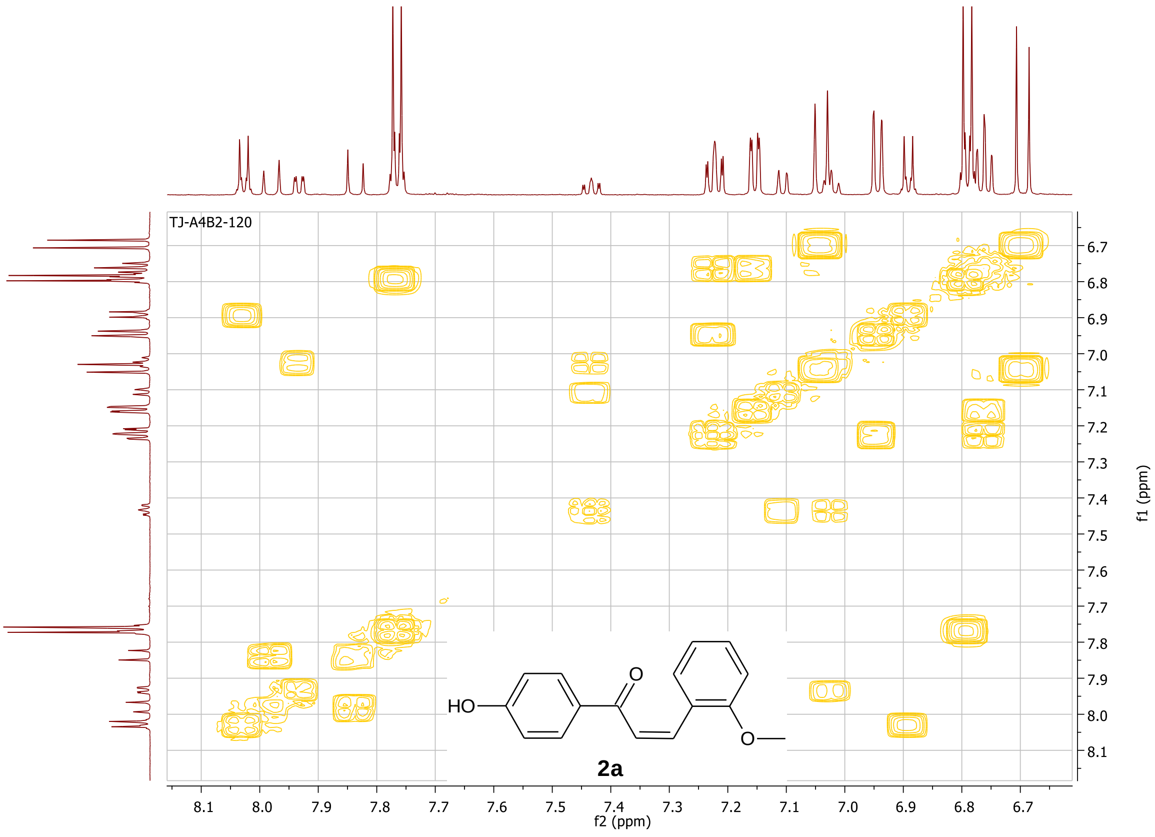

Figure S26. HMQC NMR spectrum of of *cis*-4′-hydroxy-2-methoxychalcone (**2a**) (DMSO-d_6. 600 MHz)

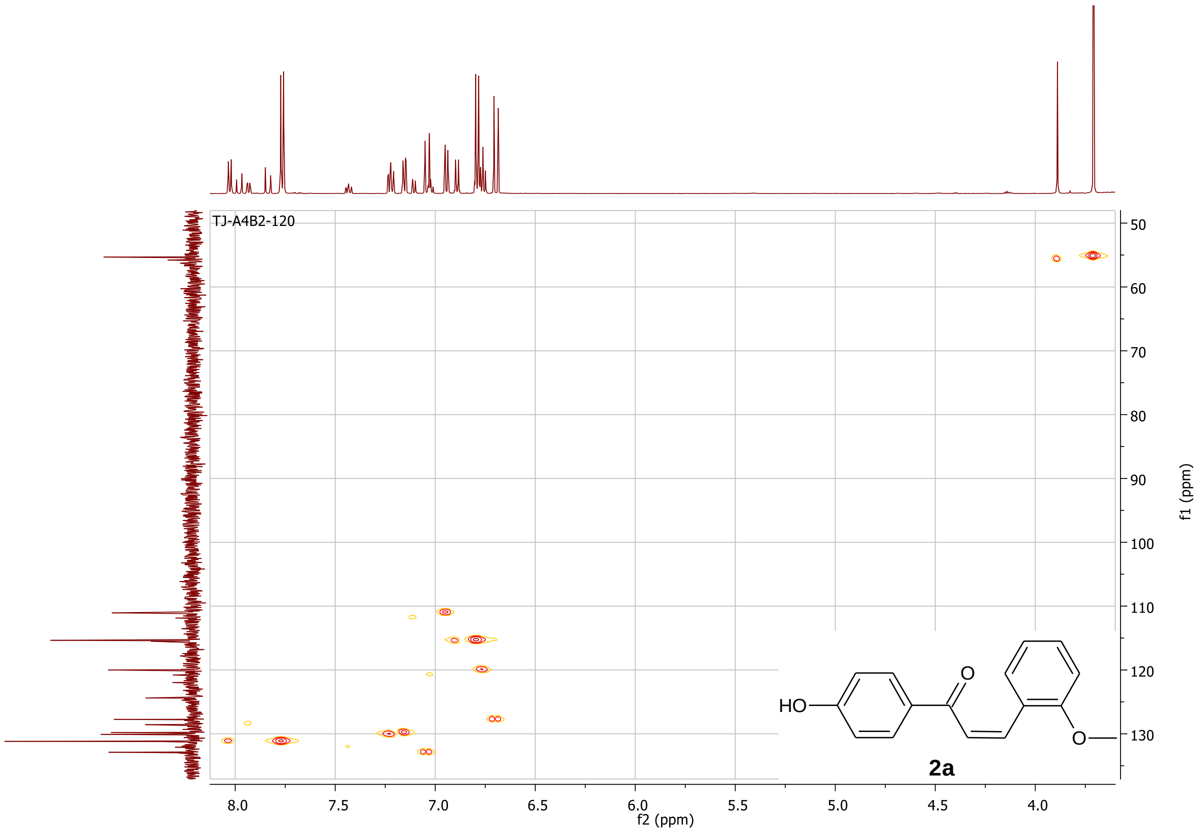

Figure S27. HMBC NMR spectrum of of *cis*-4′-hydroxy-2-methoxychalcone (**2a**) (DMSO-d_6. 600 MHz)

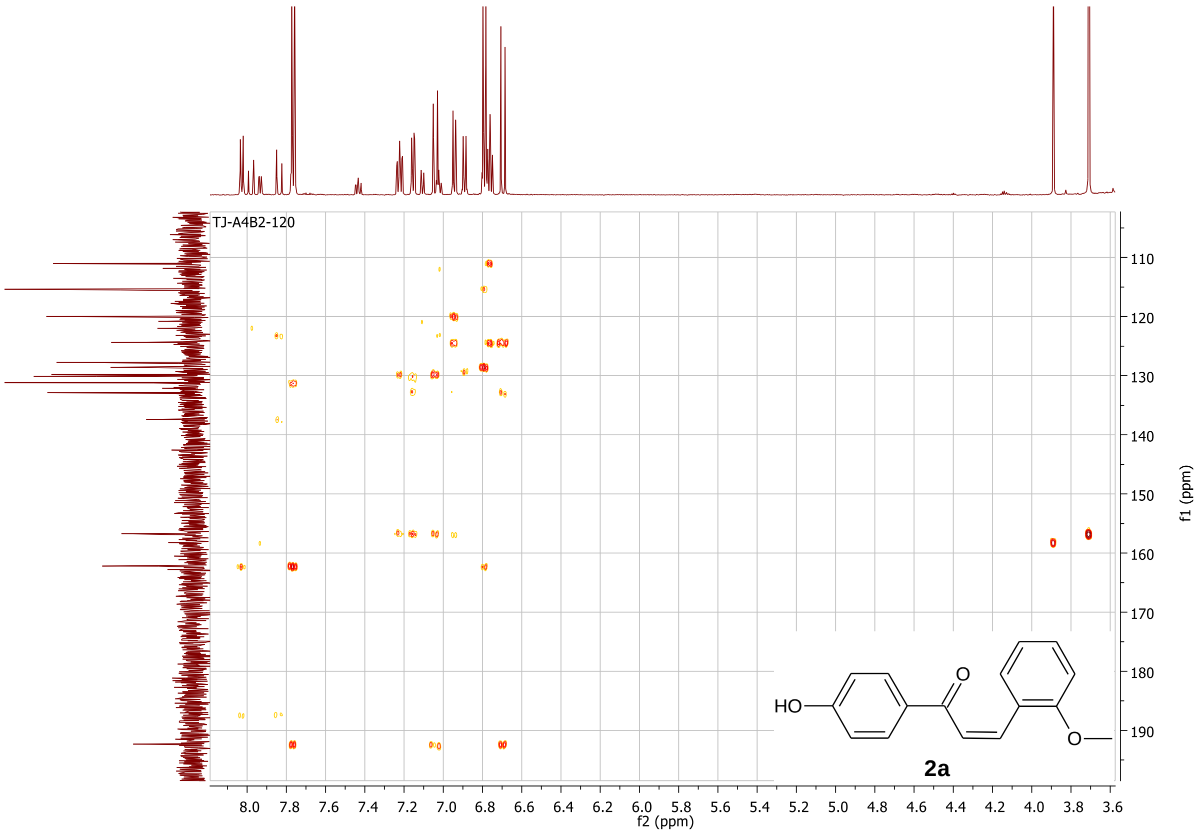

Figure S28. ^{1}H NMR spectrum of 4′-hydroxy-2-methoxydihydrochalcone (**2b**) (DMSO-d_6. 600 MHz)

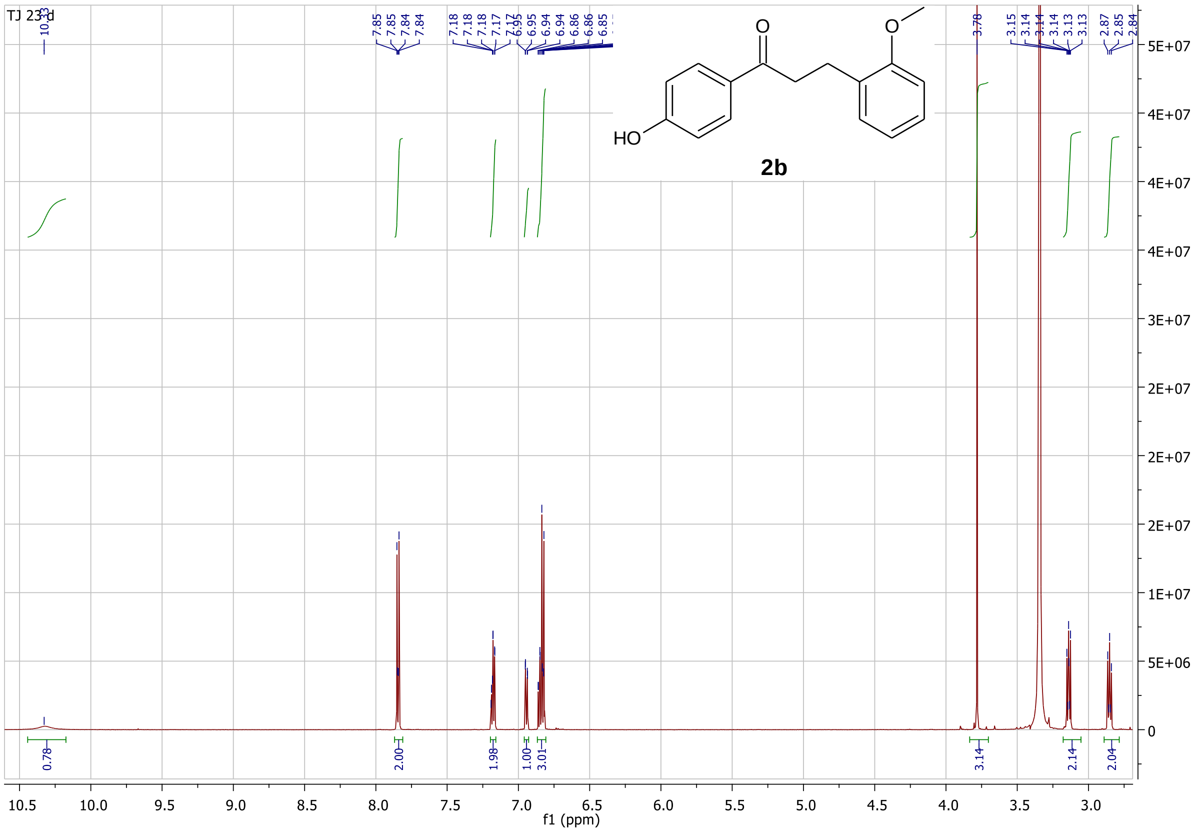

Figure S29. Part of the ^{1}H NMR spectrum of 4′-hydroxy-2-methoxydihydrochalcone (**2b**) (DMSO-*d*$_6$. 600 MHz)

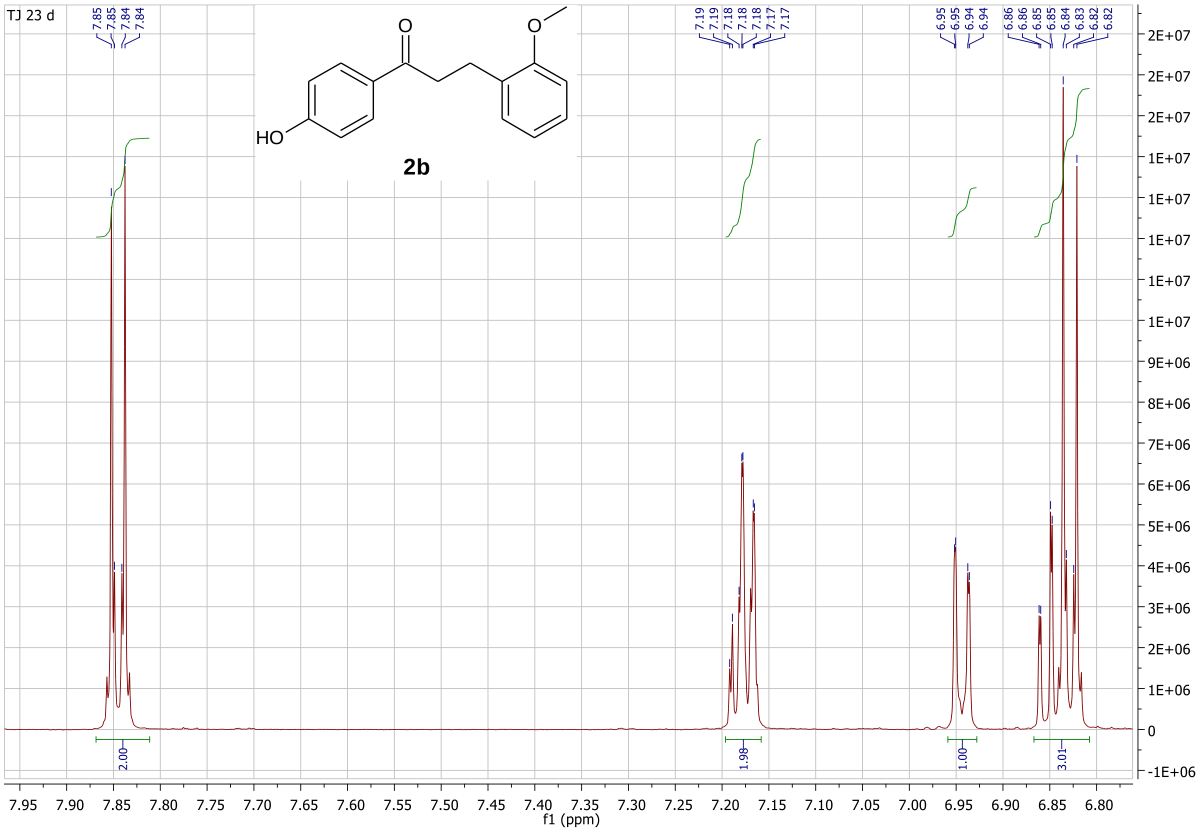

Figure S30. ^{13}C NMR spectrum of 4′-hydroxy-2-methoxydihydrochalcone (**2b**) (DMSO-*d*$_6$. 151 MHz)

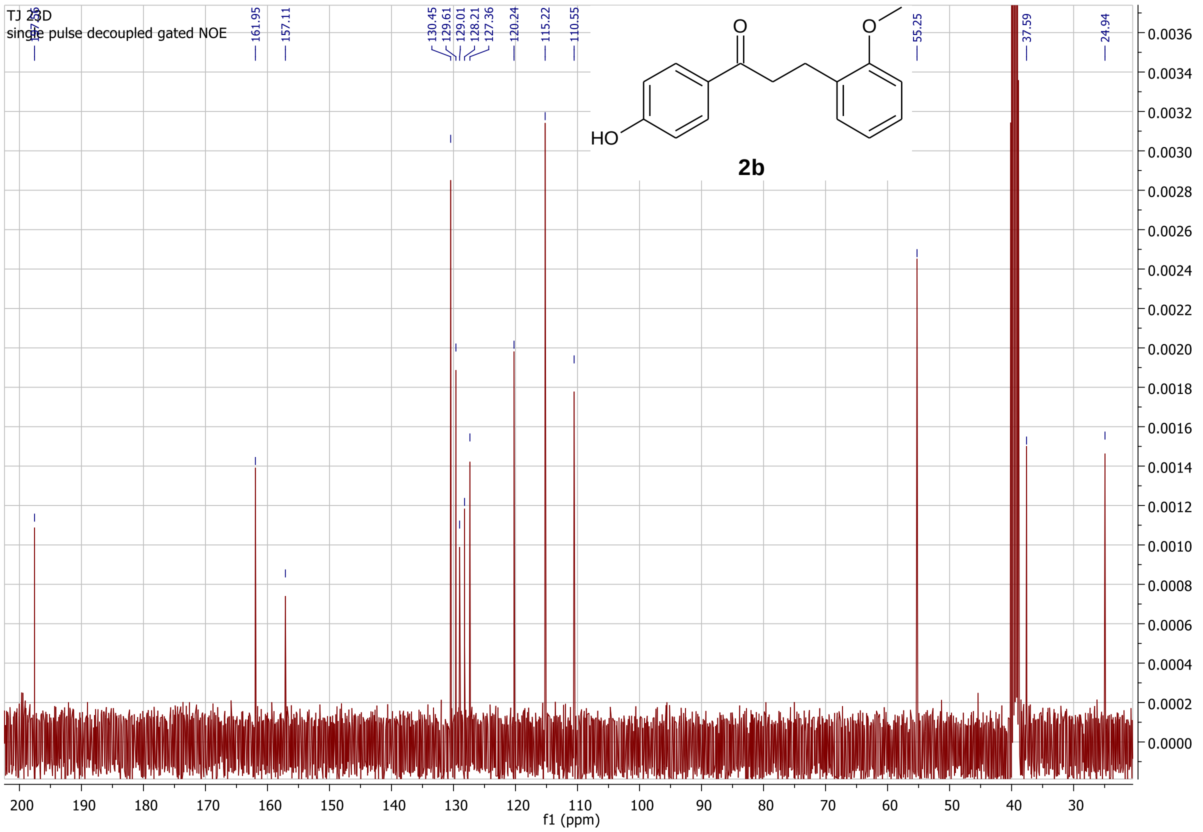

Figure S31. COSY NMR spectrum of of 4′-hydroxy-2-methoxydihydrochalcone (**2b**) (DMSO-*d*$_6$. 600 MHz)

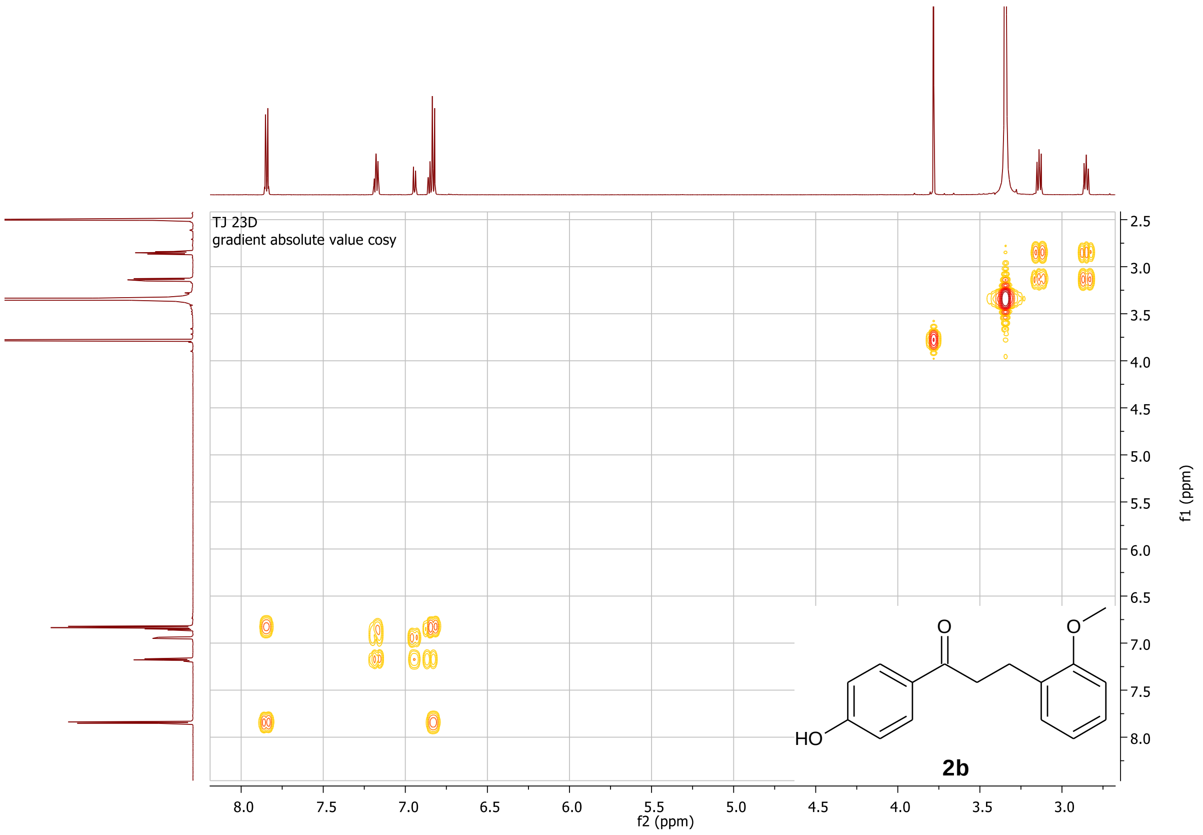

Figure S32. HMQC NMR spectrum of of 4′-hydroxy-2-methoxydihydrochalcone (**2b**) (DMSO-*d*$_6$. 600 MHz)

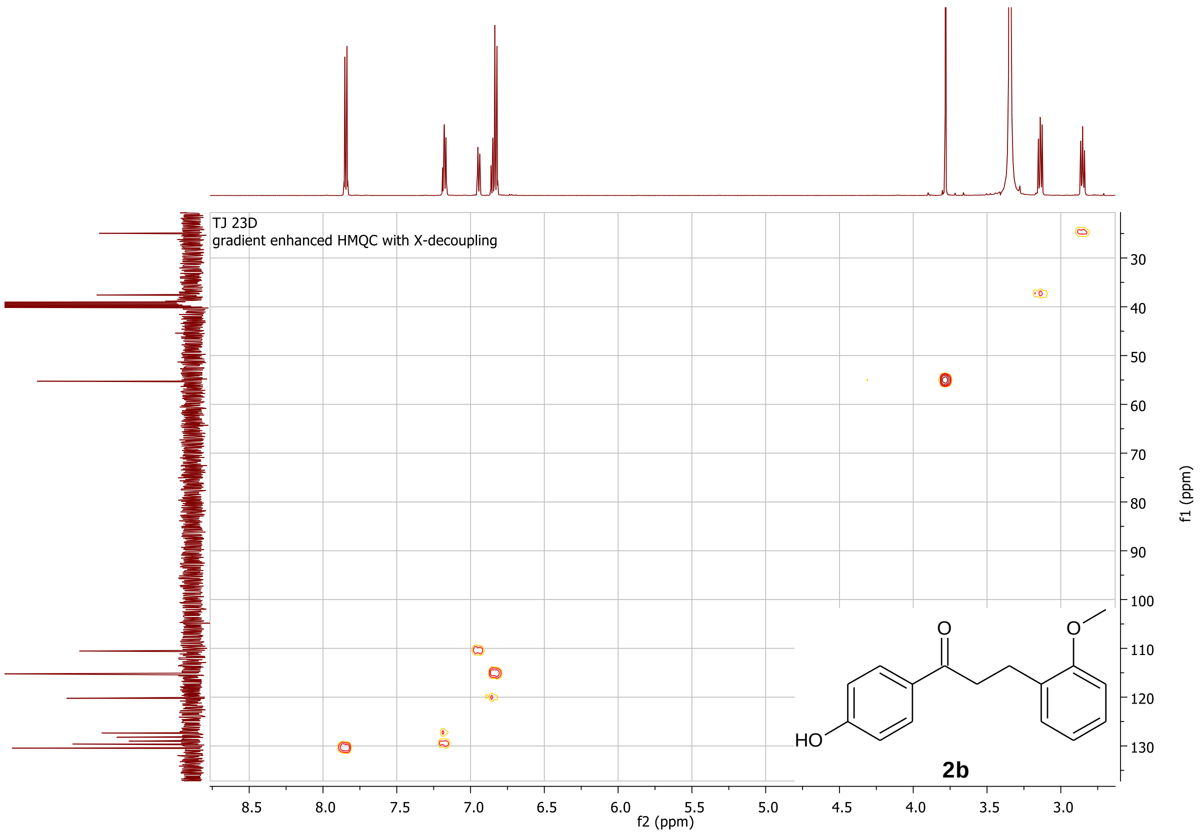

Figure S33. HMBC NMR spectrum of of 4′-hydroxy-2-methoxydihydrochalcone (**2b**) (DMSO-*d*6. 600 MHz)

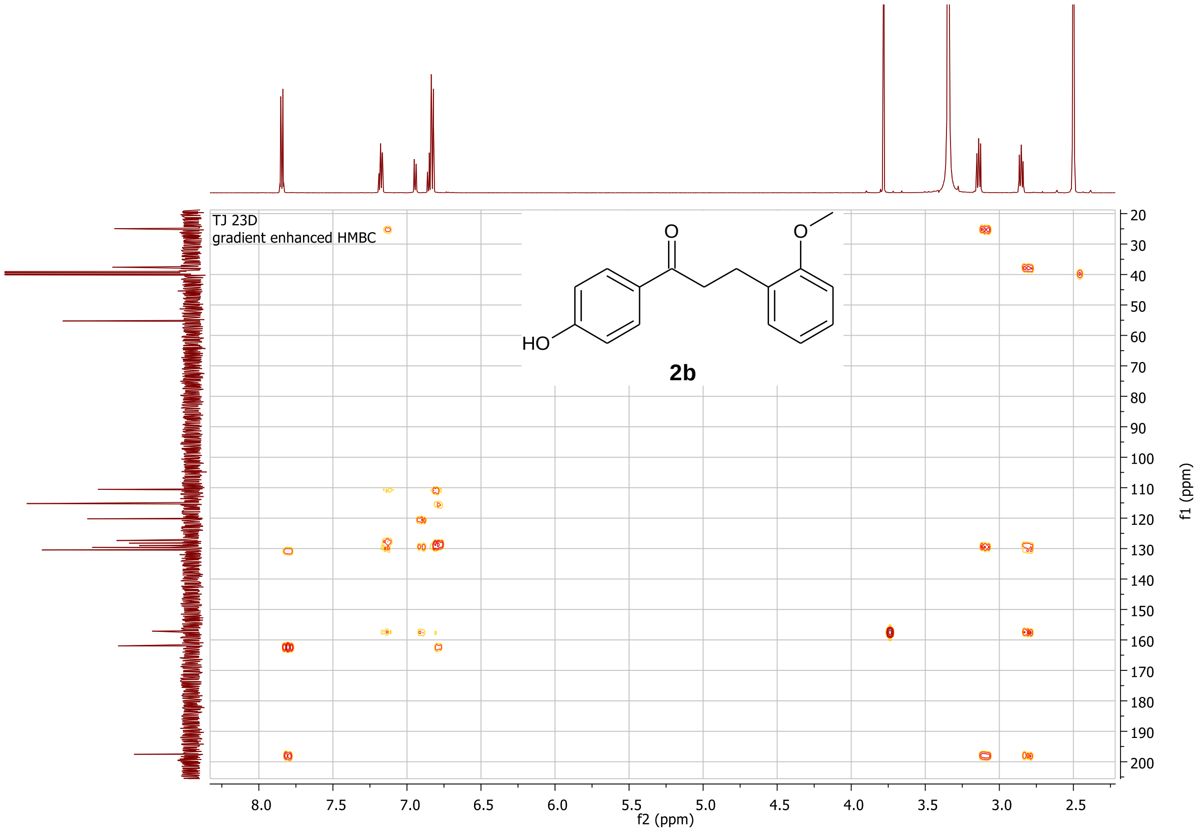

Figure S34. ^{1}H NMR spectrum of *trans*-4′-hydroxy-3-methoxychalcone (**3**) (DMSO-*d*6. 600 MHz)

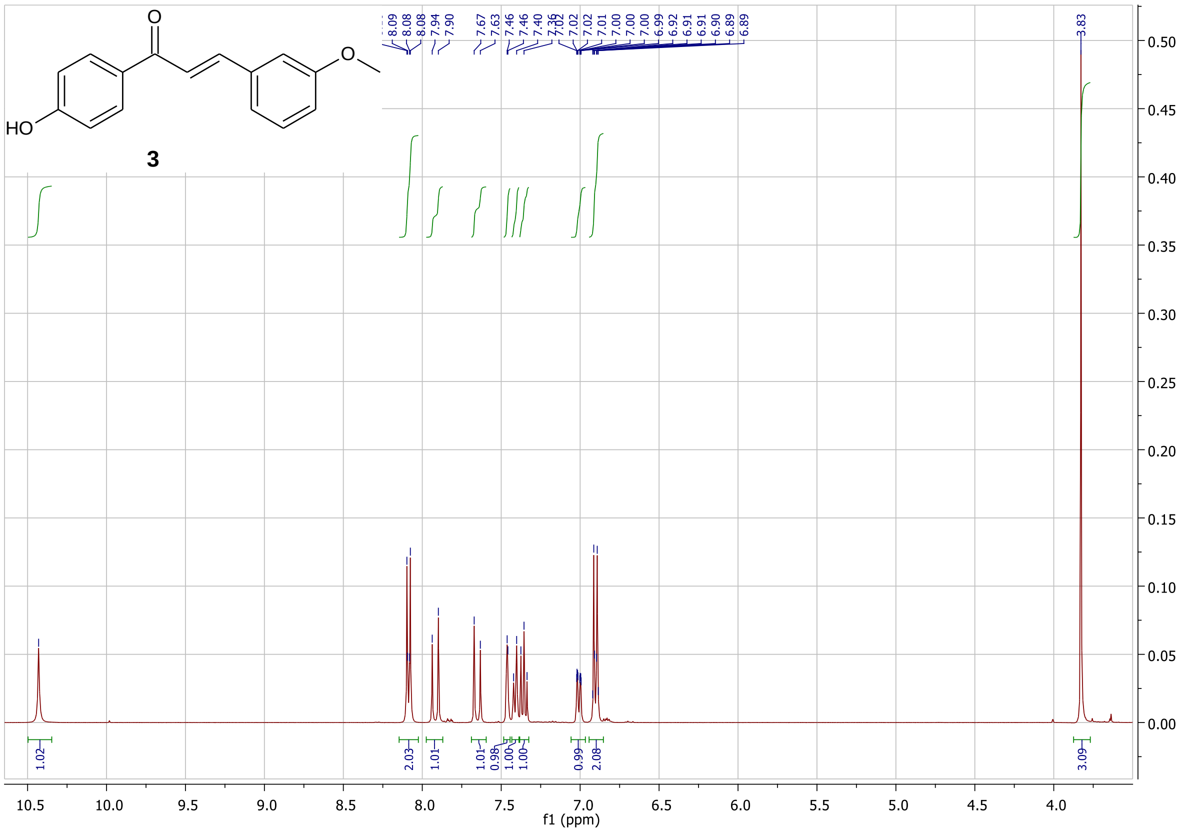

Figure S35. Part of the ^{1}H NMR spectrum of *trans*-4′-hydroxy-3-methoxychalcone (**3**) (DMSO-d_6. 600 MHz)

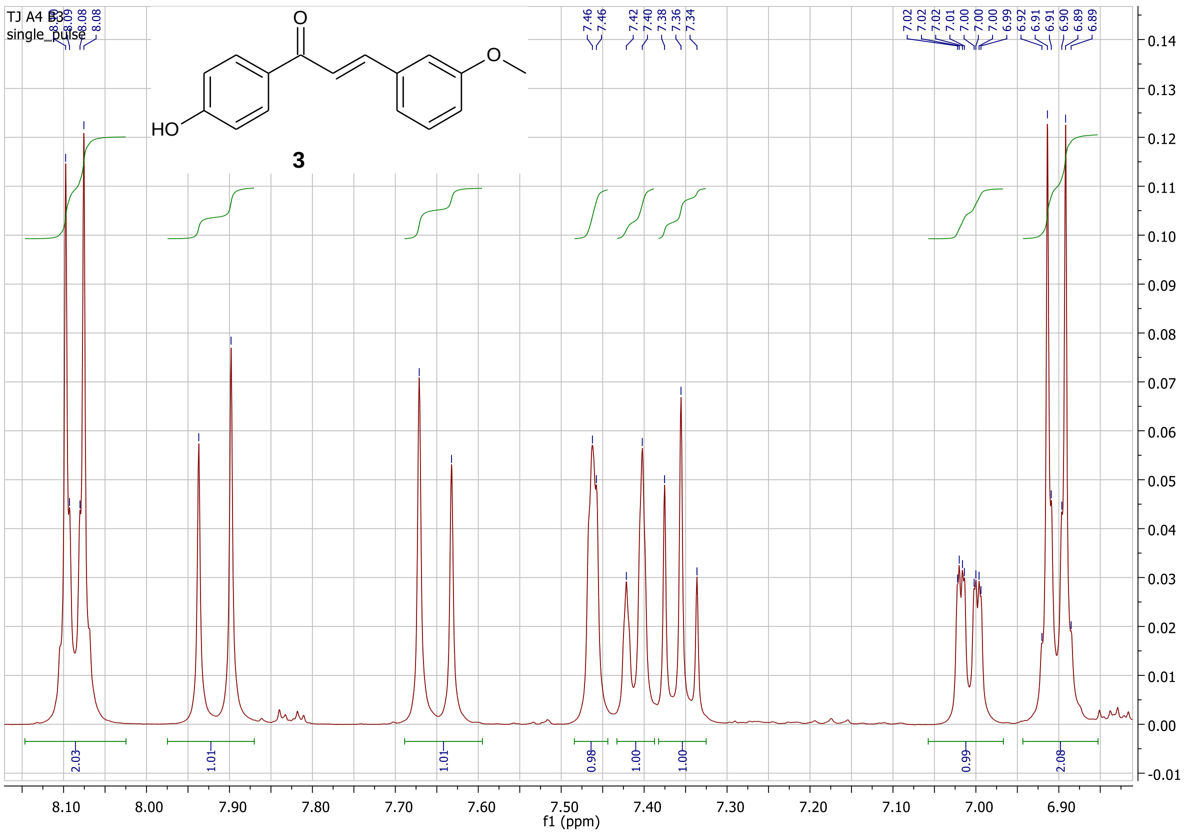

Figure S36. ^{13}C NMR spectrum of *trans*-4′-hydroxy-3-methoxychalcone (**3**) (DMSO-d_6. 151 MHz)

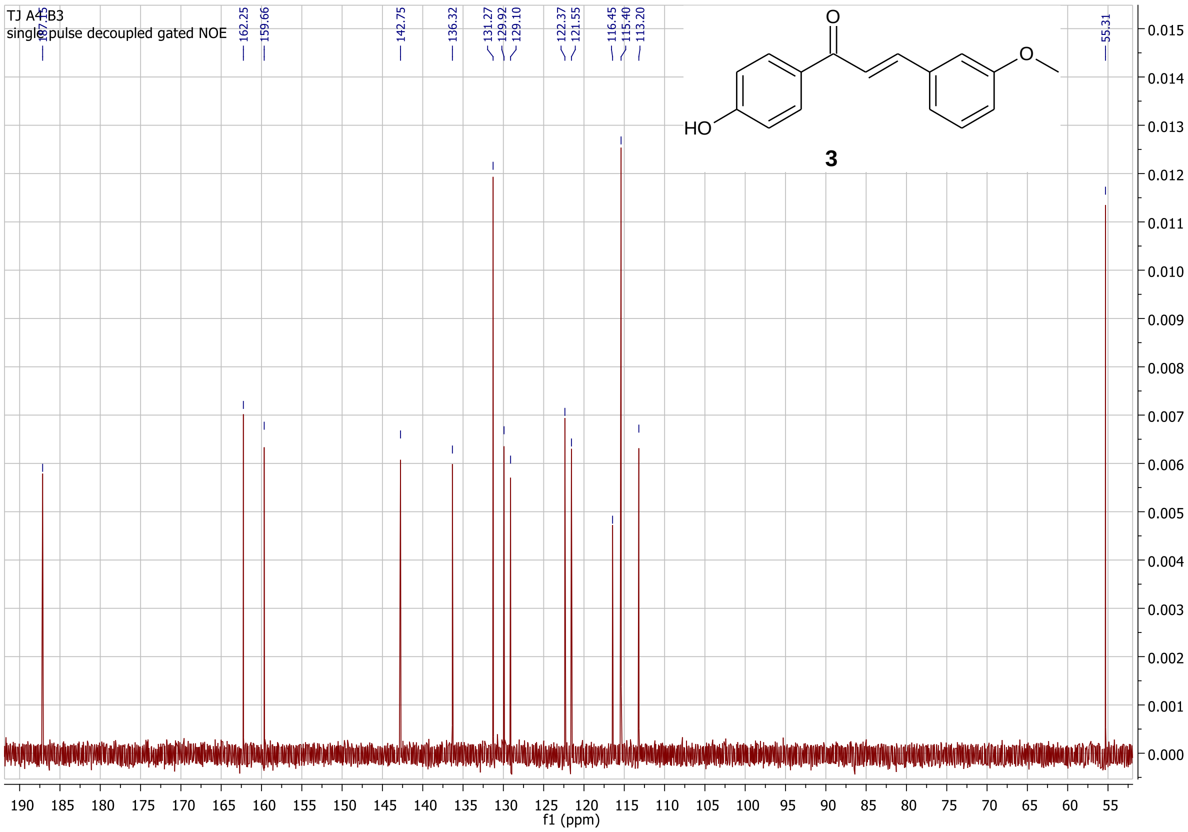

Figure S37. COSY NMR spectrum of of *trans*-4′-hydroxy-3-methoxychalcone (**3**) (DMSO-d_6. 600 MHz)

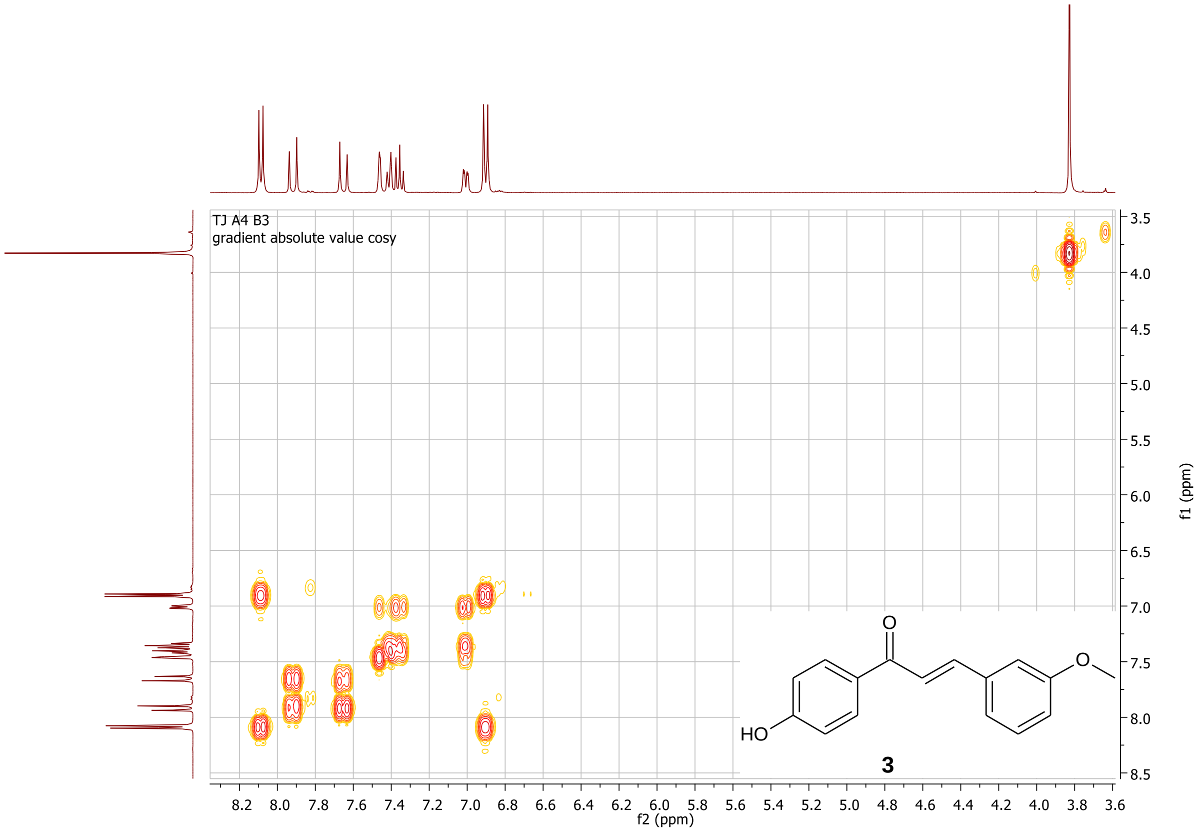

Figure S38. HMQC NMR spectrum of of *trans*-4′-hydroxy-3-methoxychalcone (**3**) (DMSO-d_6. 600 MHz)

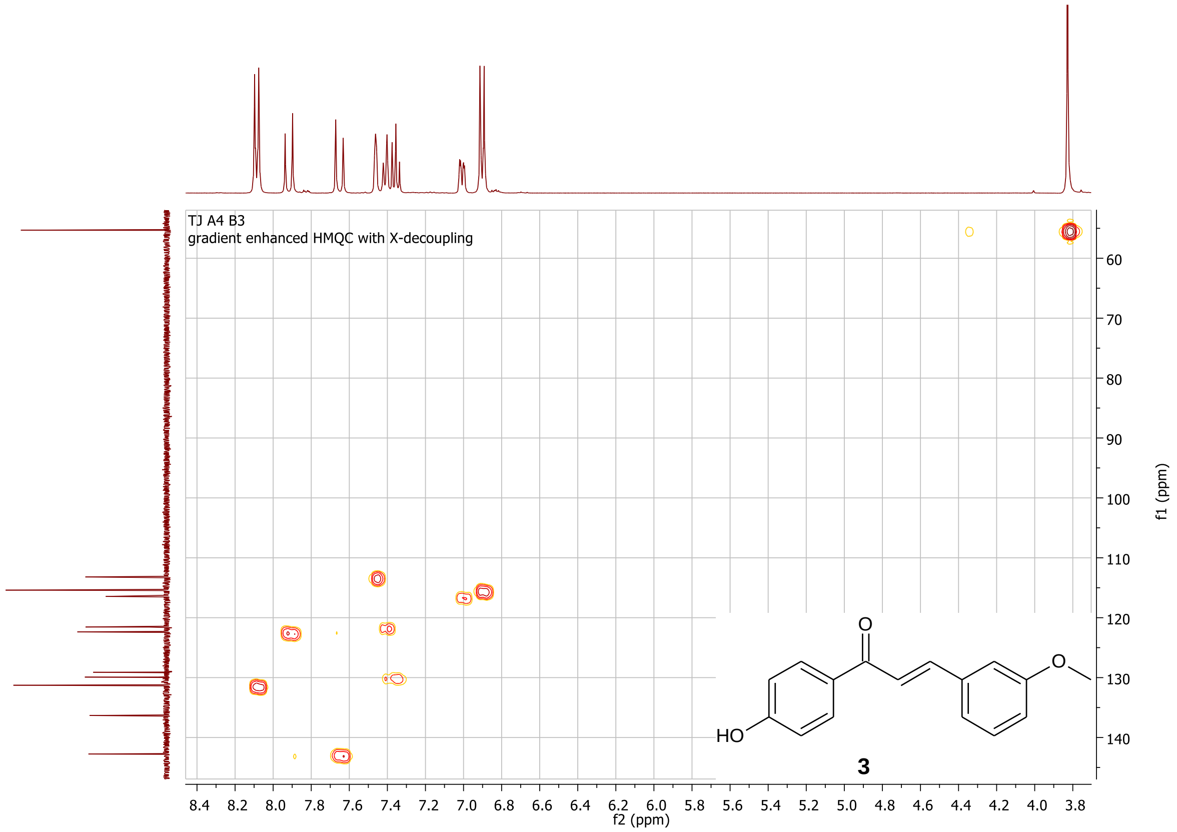

Figure S39. HMBC NMR spectrum of of *trans*-4′-hydroxy-3-methoxychalcone (**3**) (DMSO-d_6. 600 MHz)

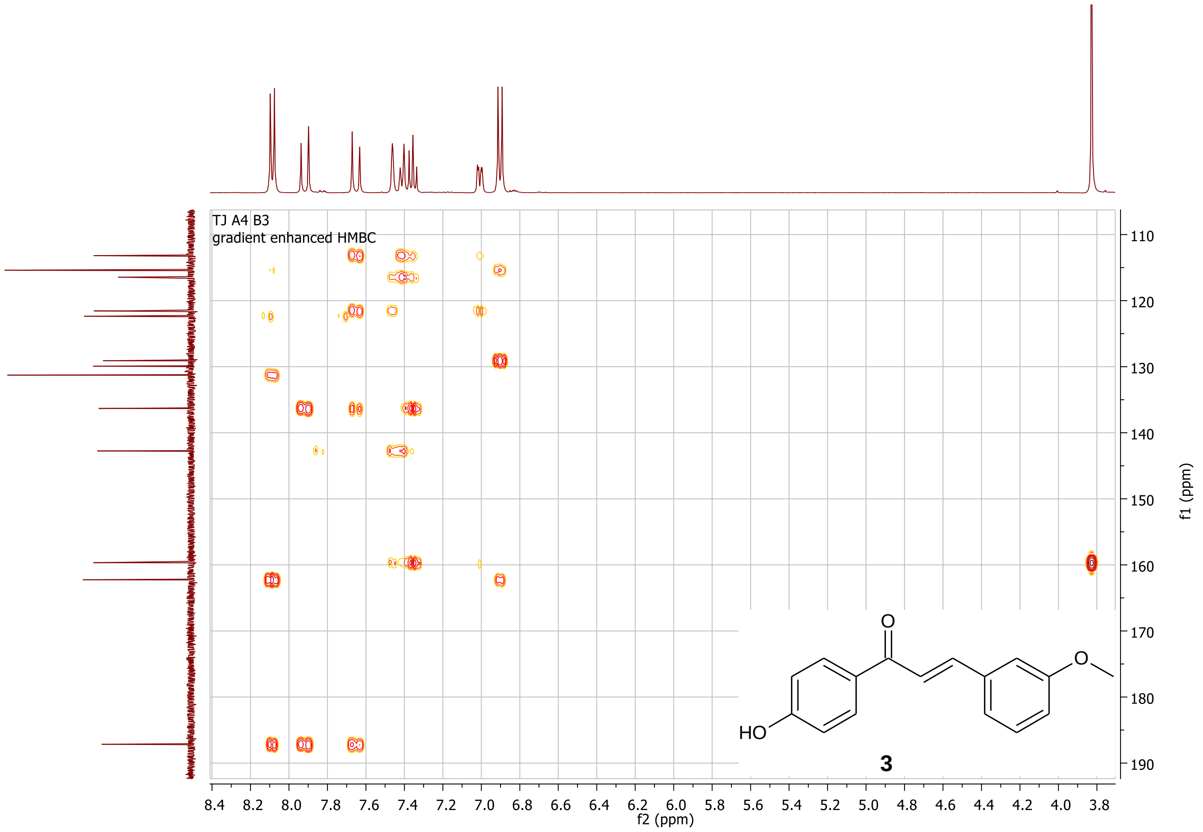

Figure S40. ^{1}H NMR spectrum of *cis*-4′-hydroxy-3-methoxychalcone (**3a**) (DMSO-d_6. 600 MHz)

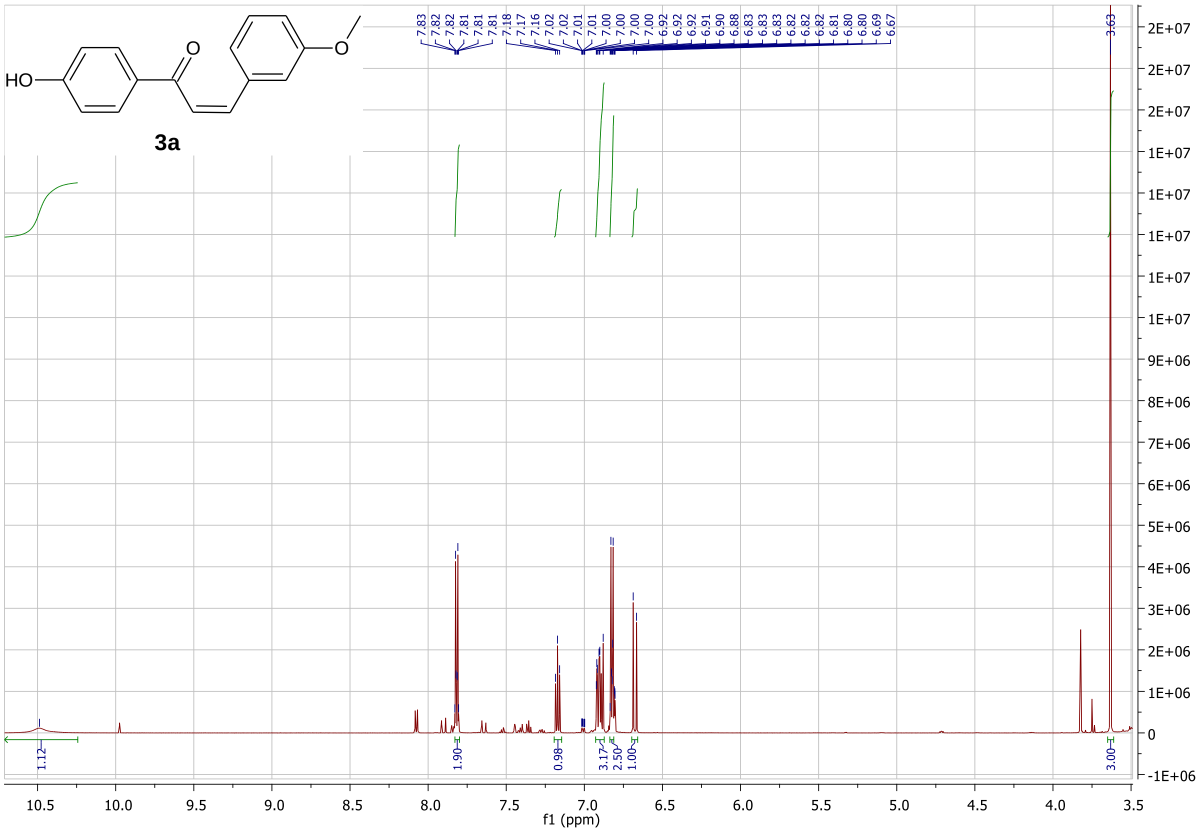

Figure S41. Part of the ^{1}H NMR spectrum of *cis*-4′-hydroxy-3-methoxychalcone (**3a**) (DMSO-d_6. 600 MHz)

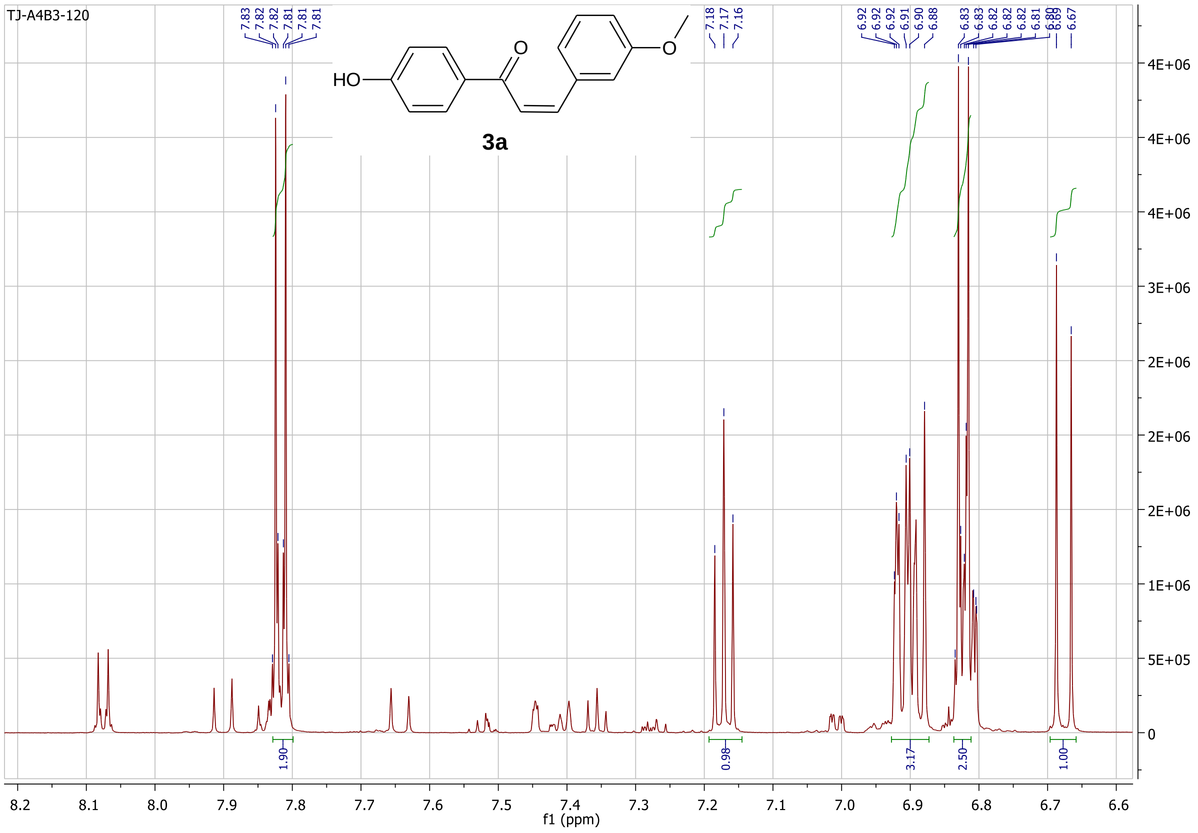

Figure S42. ^{13}C NMR spectrum of *cis*-4′-hydroxy-3-methoxychalcone (**3a**) (DMSO-d_6. 151 MHz)

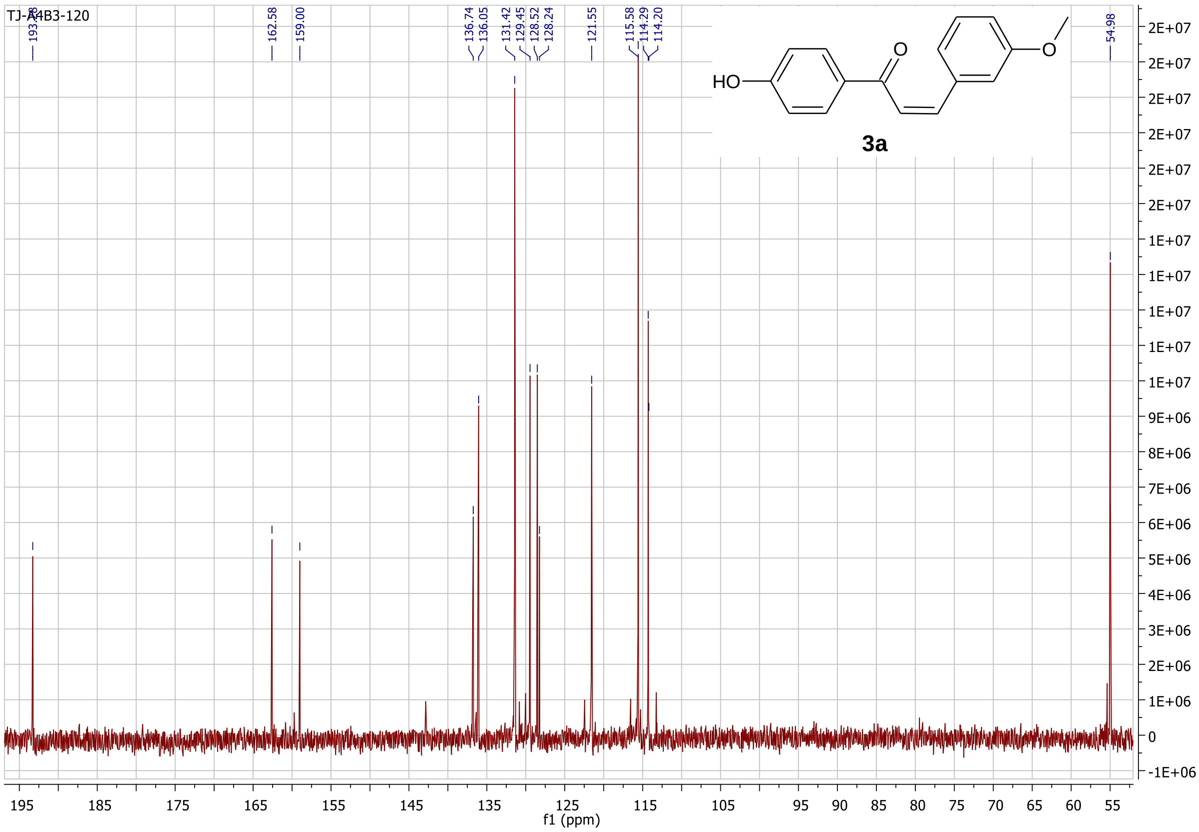

Figure S43. COSY NMR spectrum of of *cis*-4′-hydroxy-3-methoxychalcone (**3a**) (DMSO-d_6. 600 MHz)

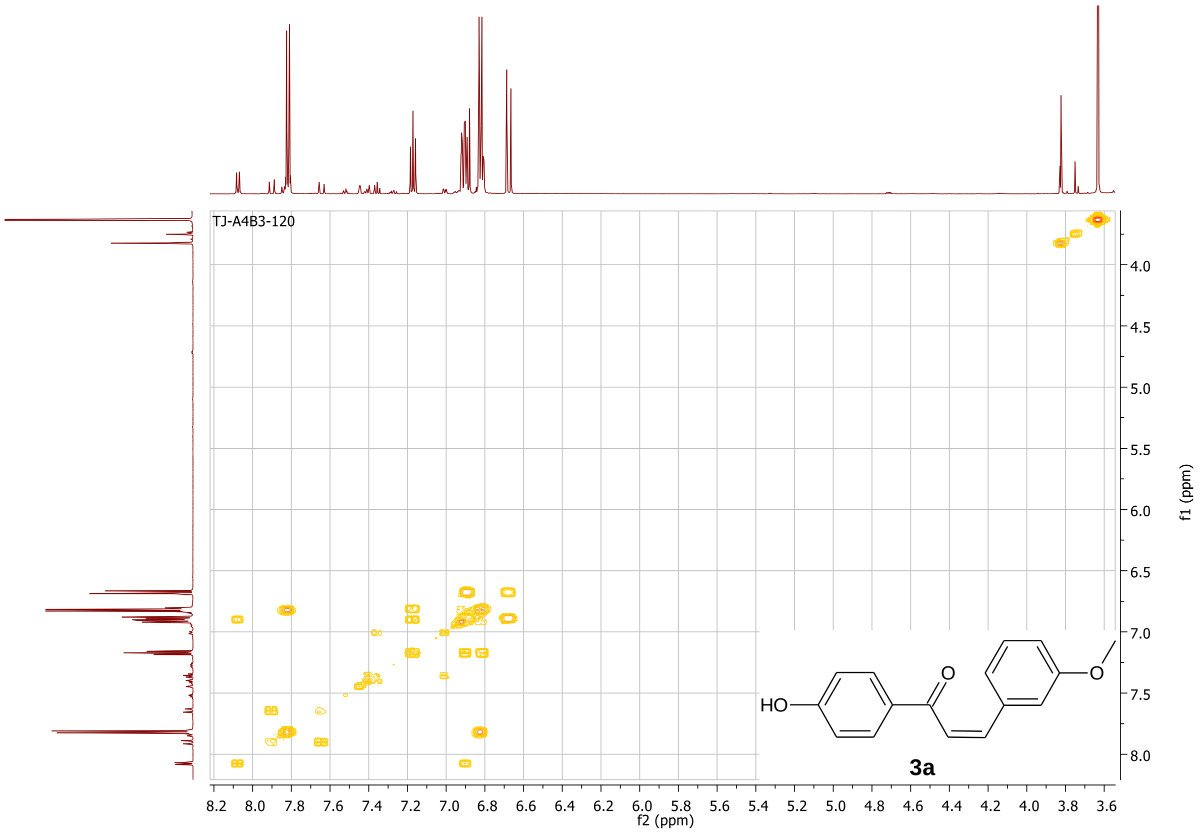

Figure S44. HMQC NMR spectrum of of *cis*-4′-hydroxy-3-methoxychalcone (**3a**) (DMSO-d_6. 600 MHz)

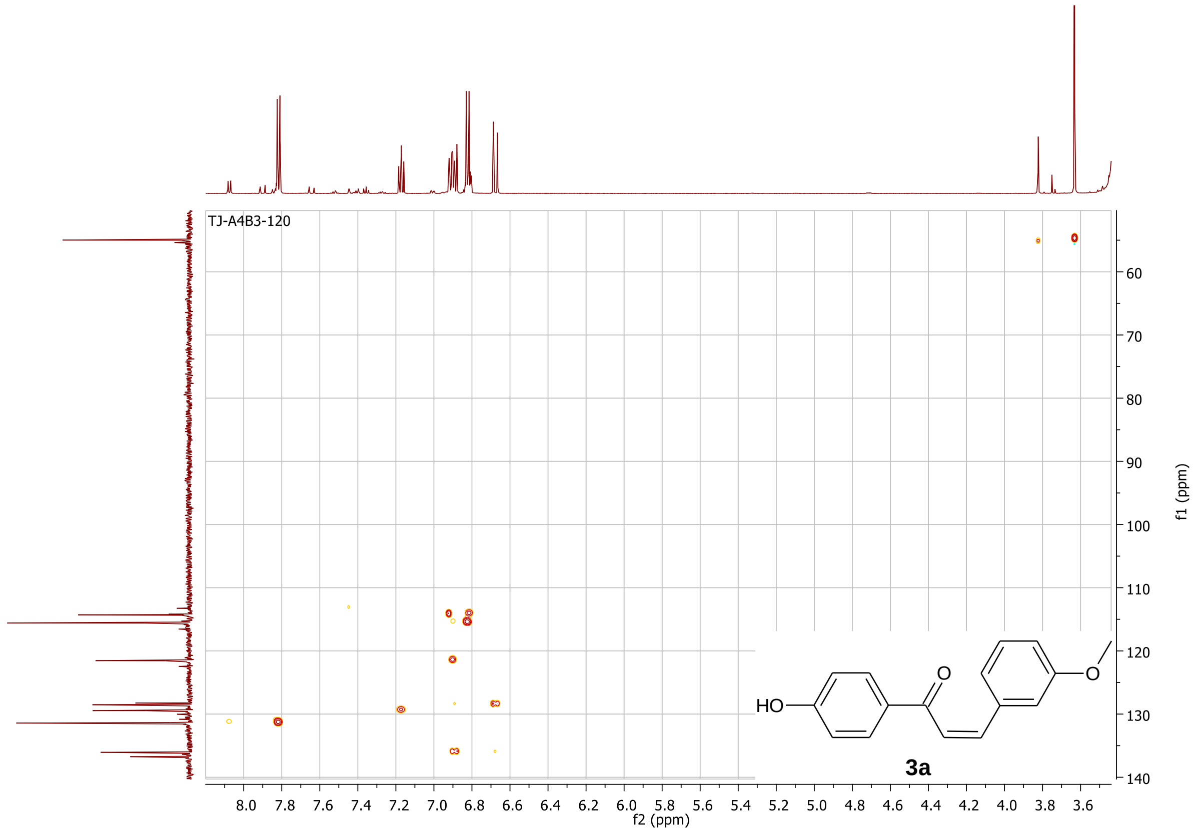

Figure S45. HMBC NMR spectrum of of *cis*-4′-hydroxy-3-methoxychalcone (**3a**) (DMSO-d_6. 600 MHz)

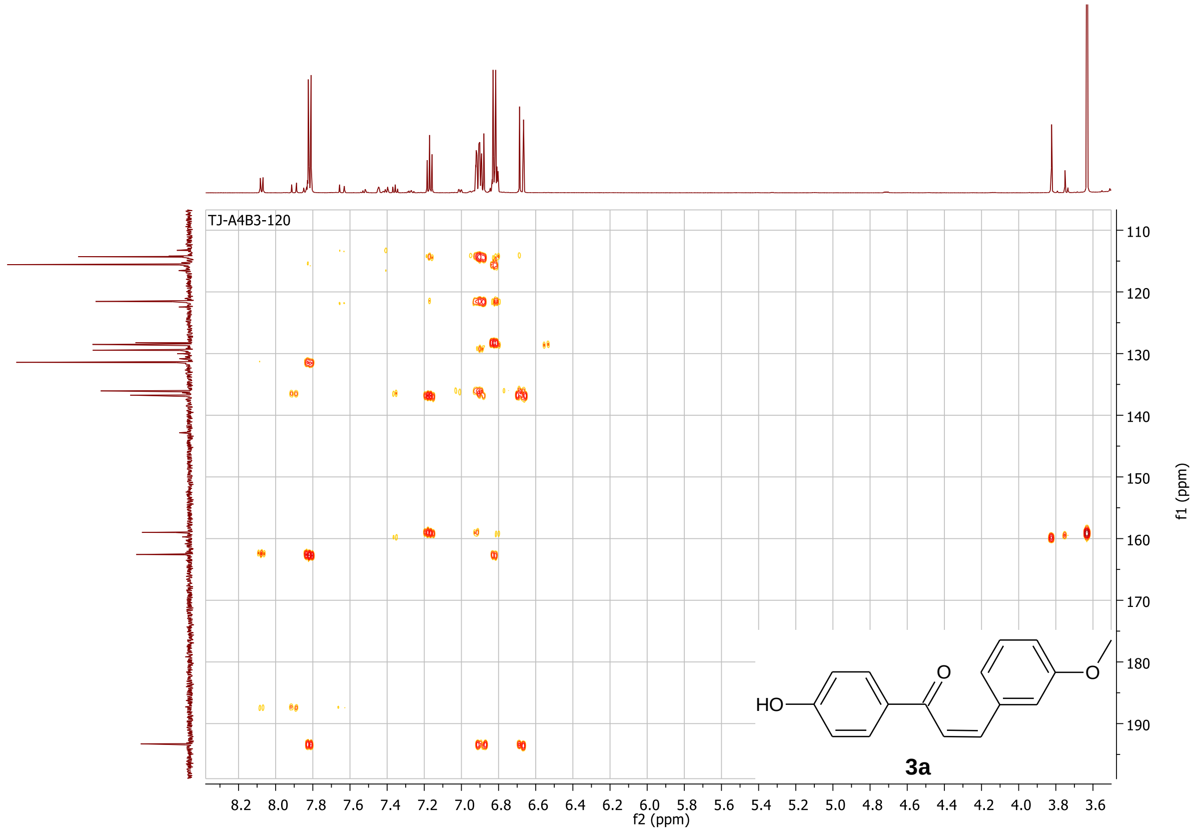

Figure S46. ^{1}H NMR spectrum of 4′-hydroxy-3-methoxydihydrochalcone (**3b**) (DMSO-d_6. 600 MHz)

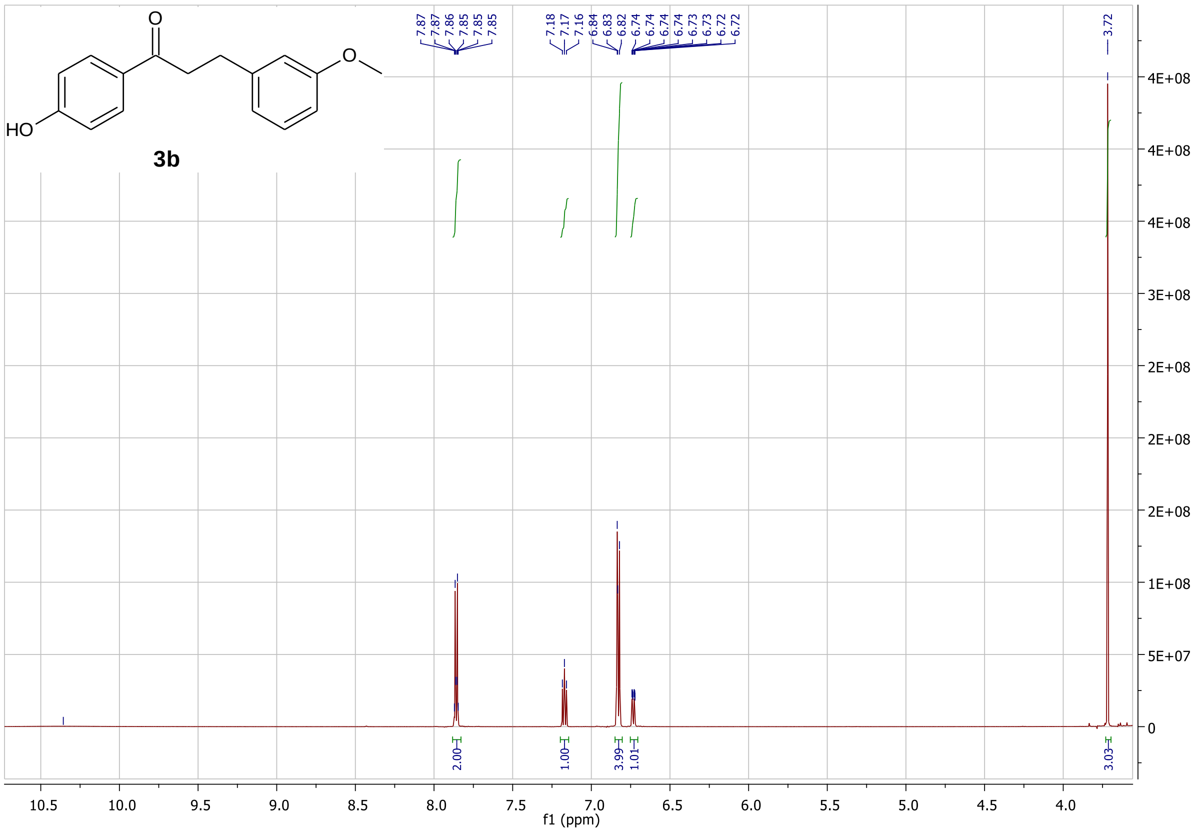

Figure S47. Part of the ¹H NMR spectrum of 4′-hydroxy-3-methoxydihydrochalcone (**3b**) (DMSO-d_6. 600 MHz)

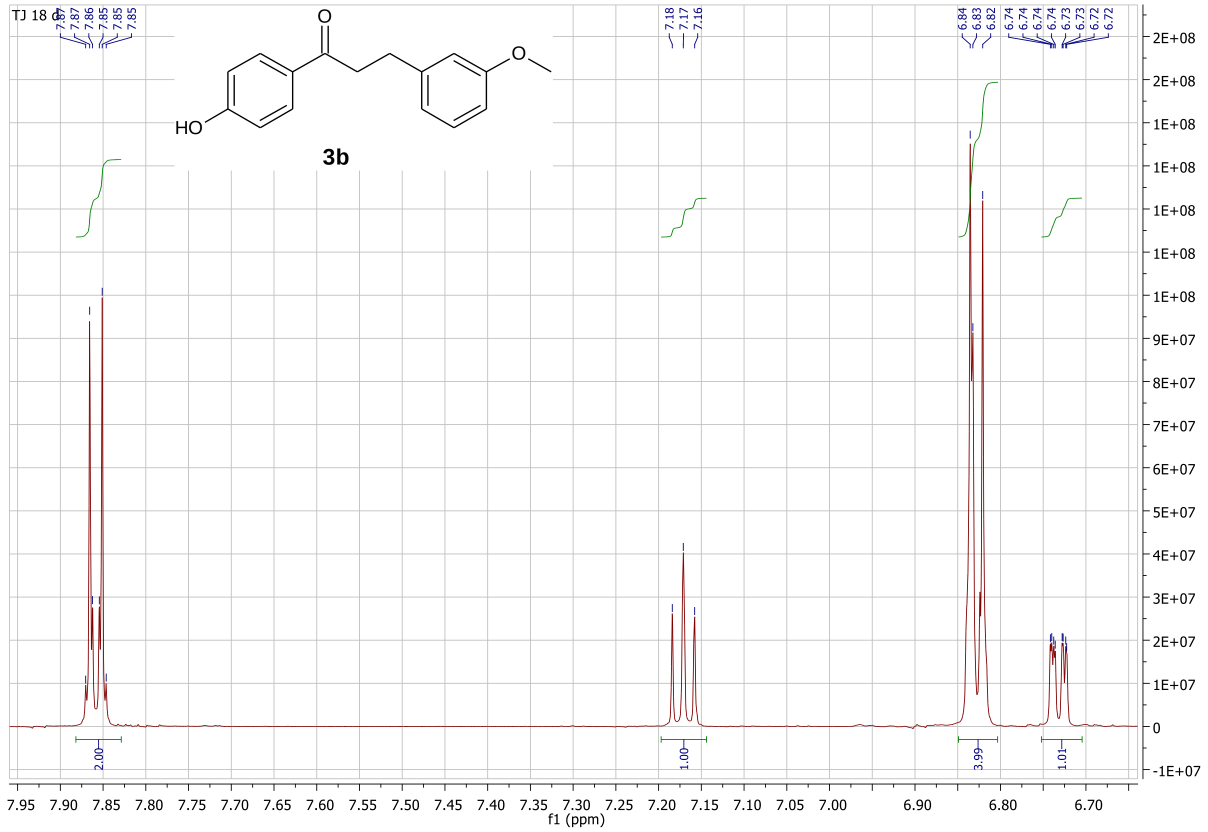

Figure S48. Part of the ¹H NMR spectrum of 4′-hydroxy-3-methoxydihydrochalcone (**3b**) (CDCl$_3$. 600 MHz)

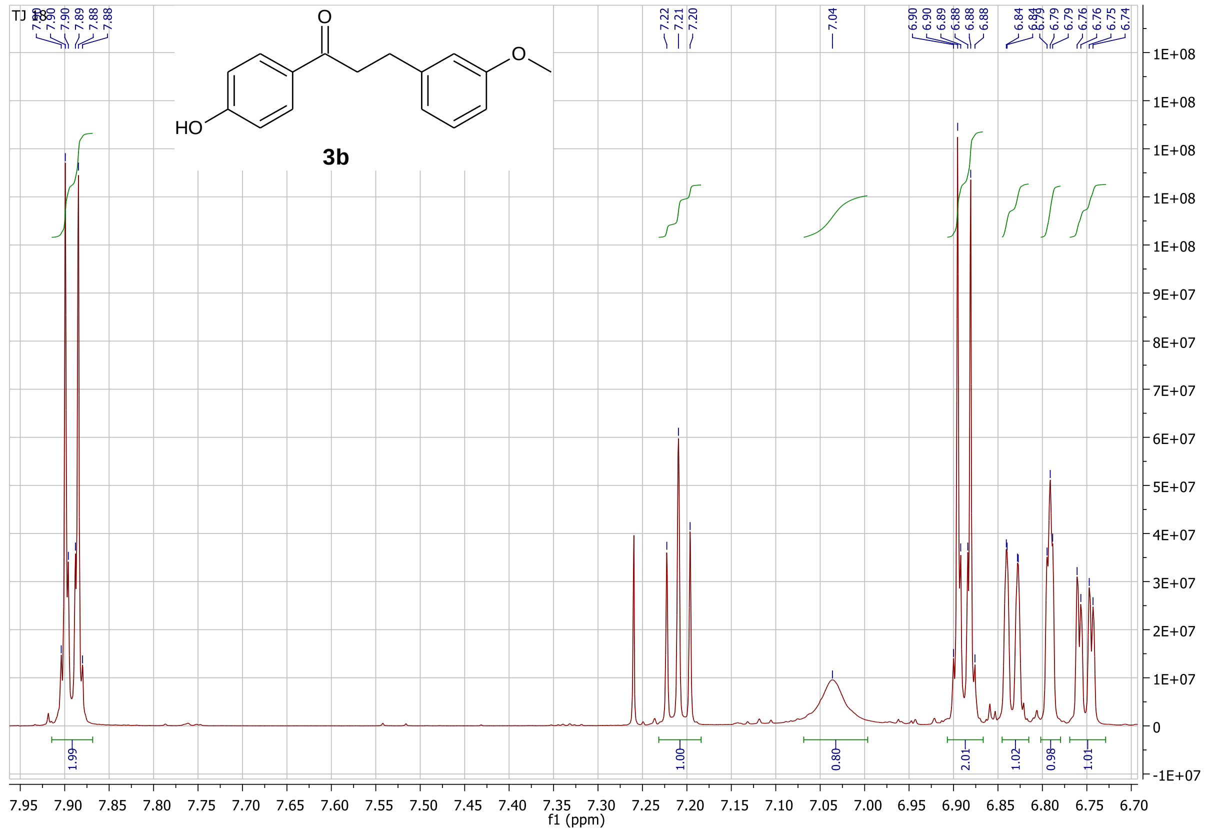

Figure S49. ^{13}C NMR spectrum of 4′-hydroxy-3-methoxydihydrochalcone (**3b**) (DMSO-d_6. 151 MHz)

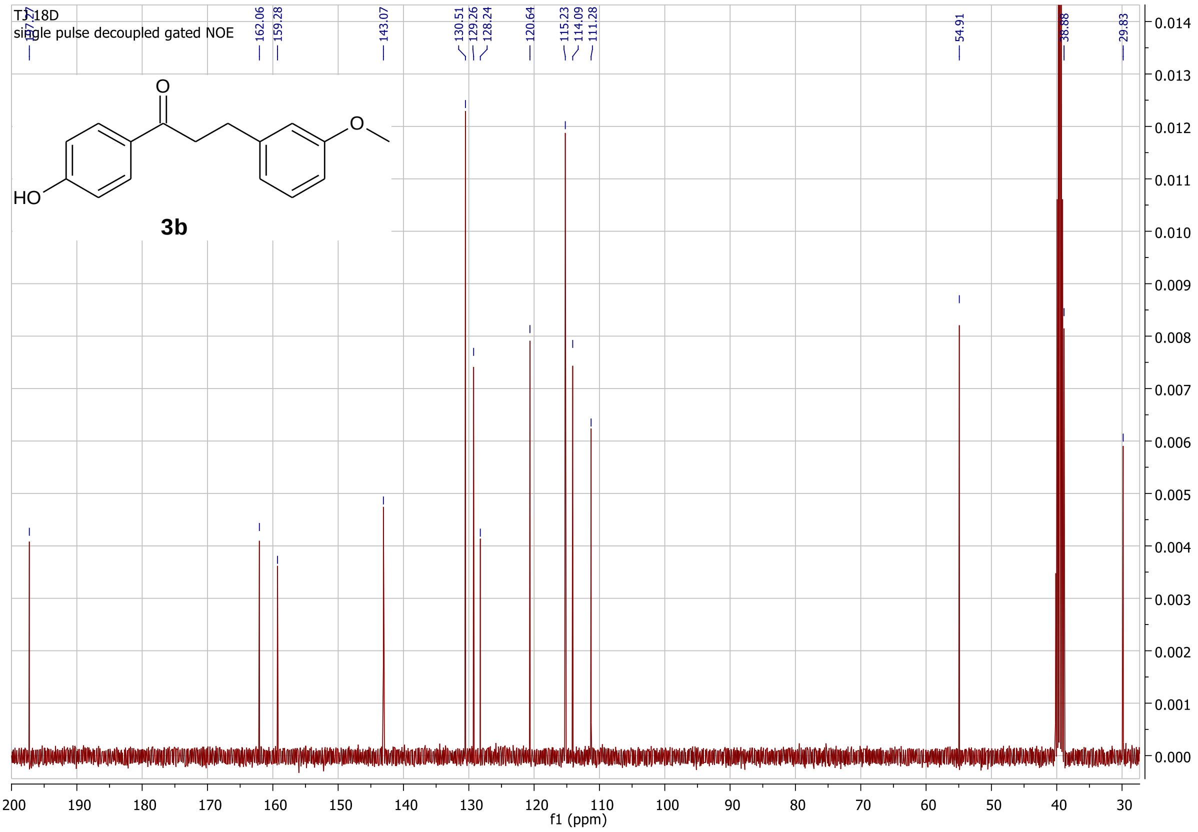

Figure S50. ^{13}C NMR spectrum of 4′-hydroxy-3-methoxydihydrochalcone (**3b**) (CDCl$_3$. 151 MHz)

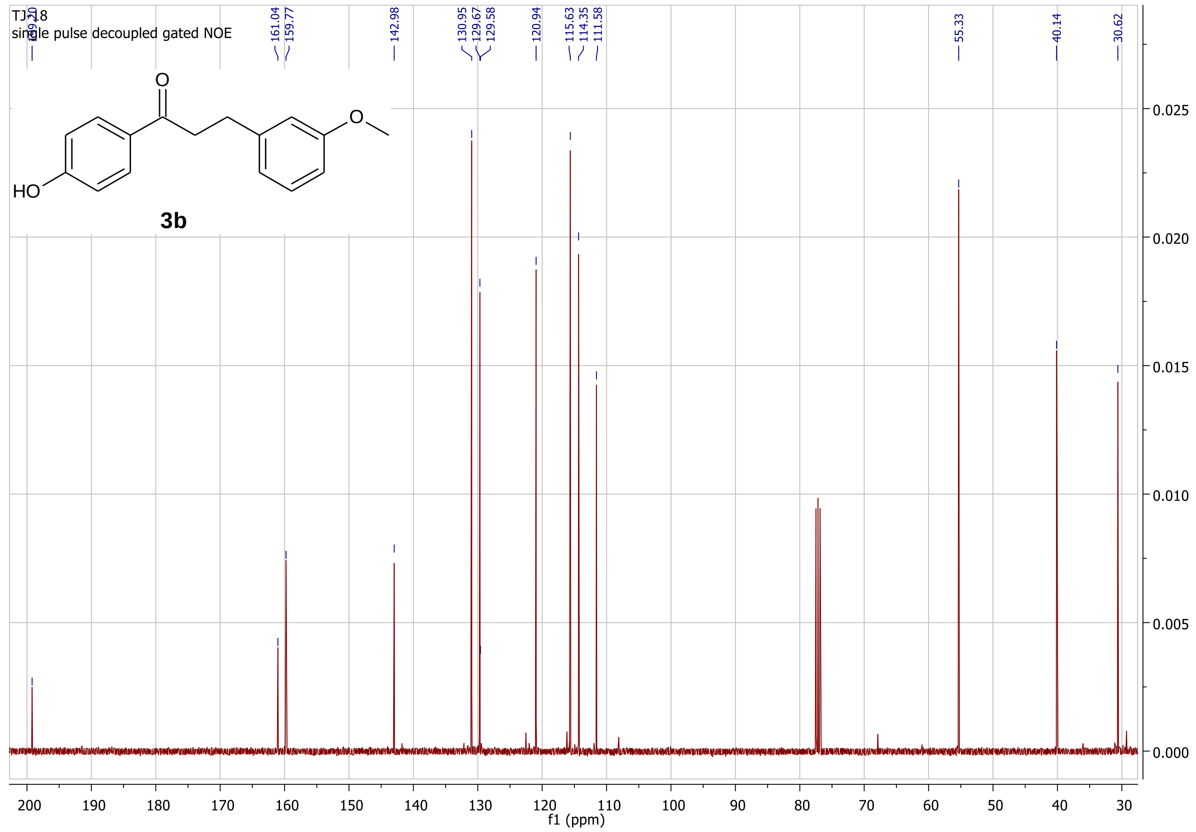

Figure S51. COSY NMR spectrum of of 4′-hydroxy-3-methoxydihydrochalcone (**3b**) (DMSO-d_6. 600 MHz)

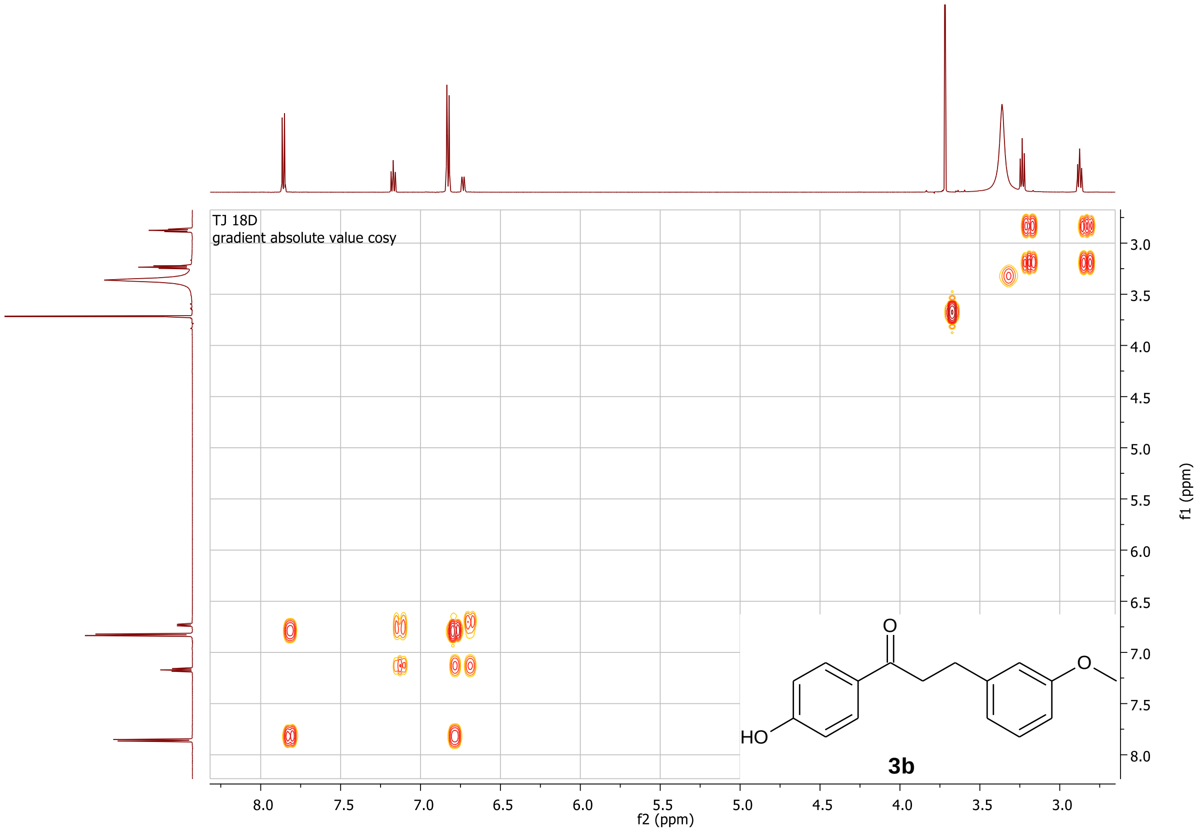

Figure S52. HMQC NMR spectrum of of 4′-hydroxy-3-methoxydihydrochalcone (**3b**) (DMSO-d_6. 600 MHz)

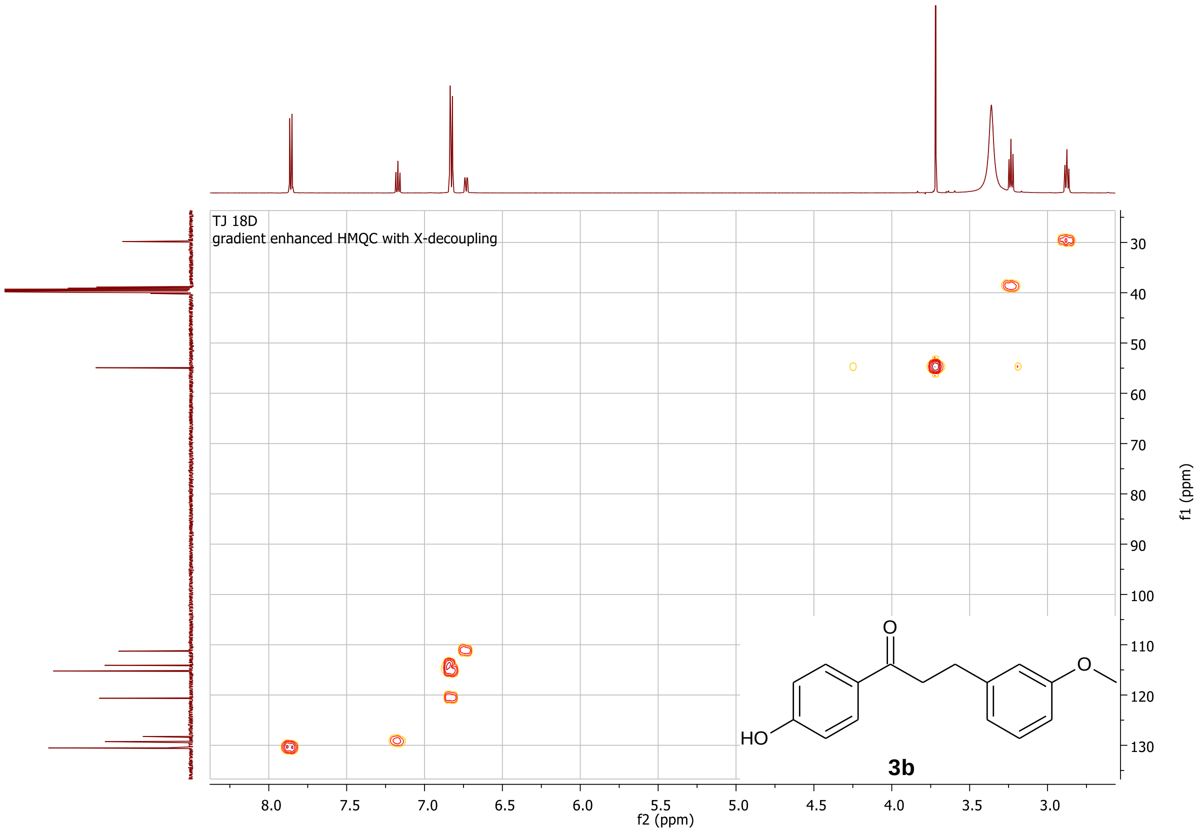

Figure S53. HMBC NMR spectrum of of 4′-hydroxy-3-methoxydihydrochalcone (**3b**) (DMSO-d_6. 600 MHz)

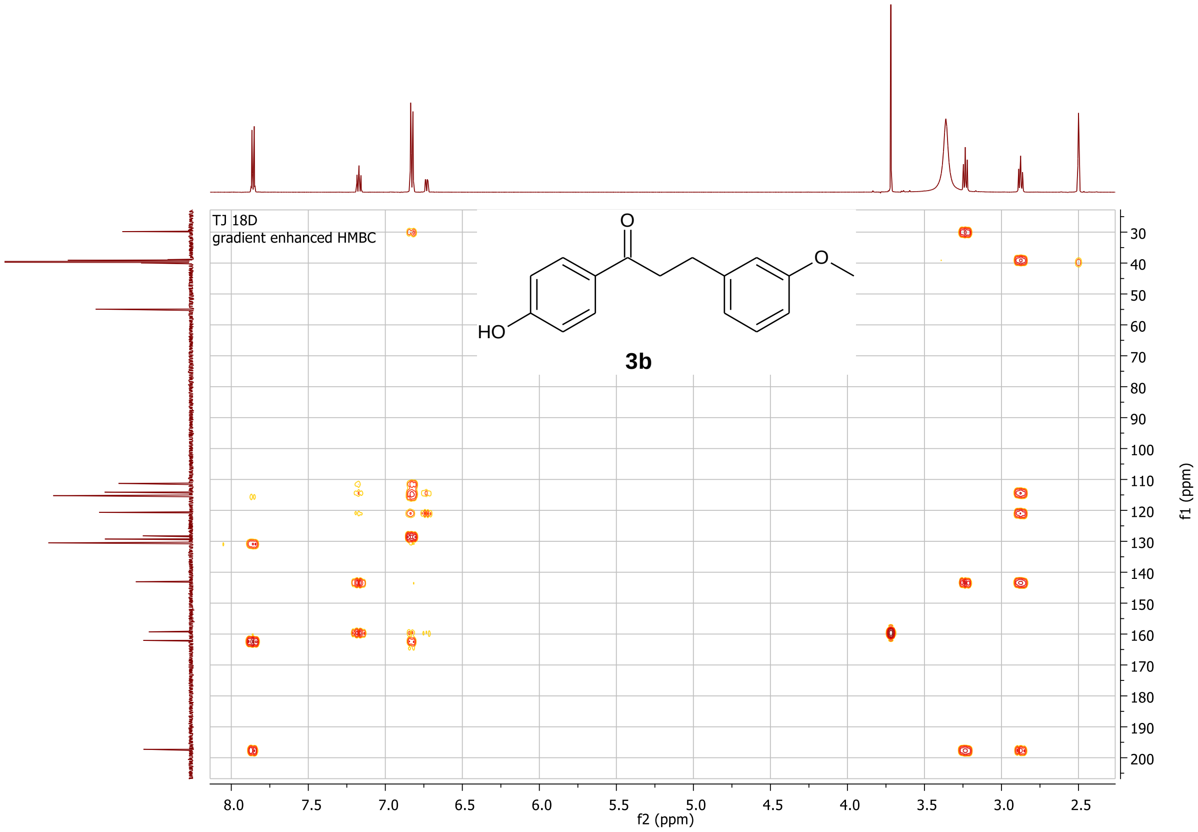

Figure S54. ^{1}H NMR spectrum of *trans*-4′-hydroxy-4-methoxychalcone (**4**) (DMSO-d_6. 600 MHz)

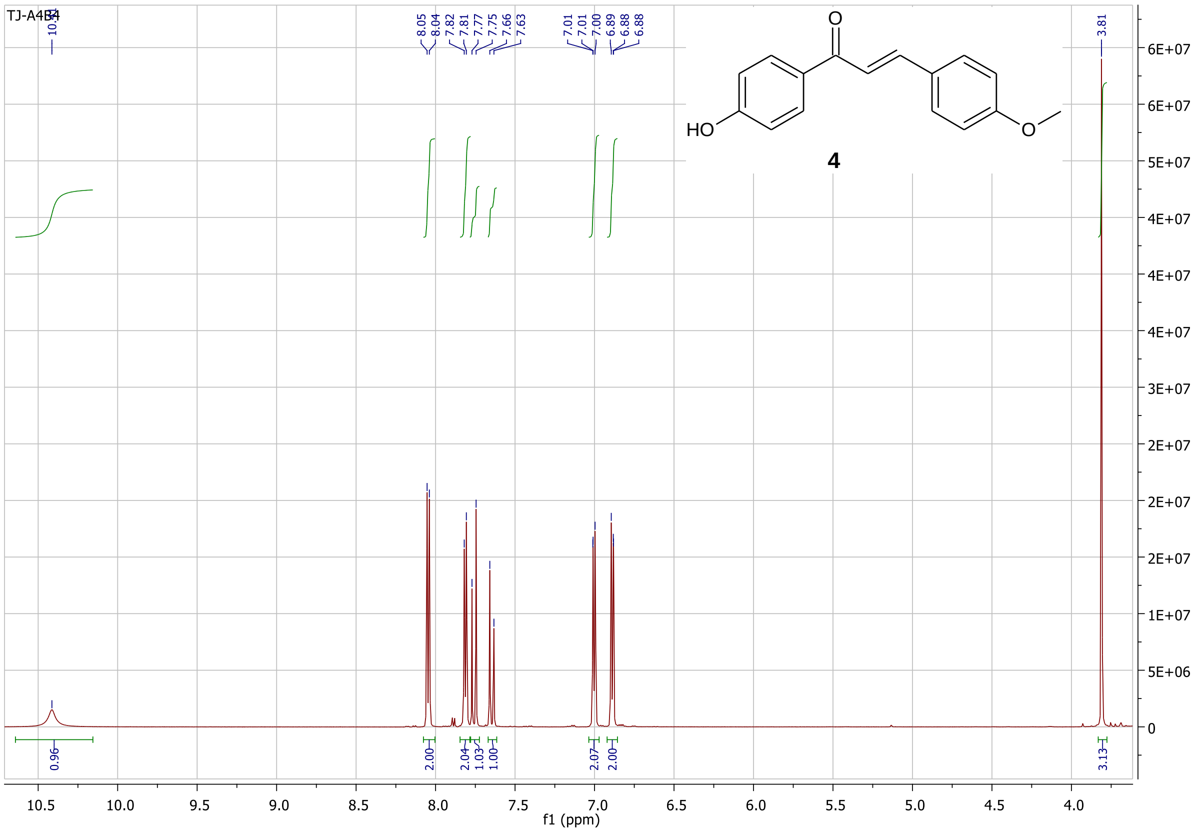

Figure S55. Part of the ^{1}H NMR spectrum of *trans*-4′-hydroxy-4-methoxychalcone (**4**) (DMSO-d_6. 600 MHz)

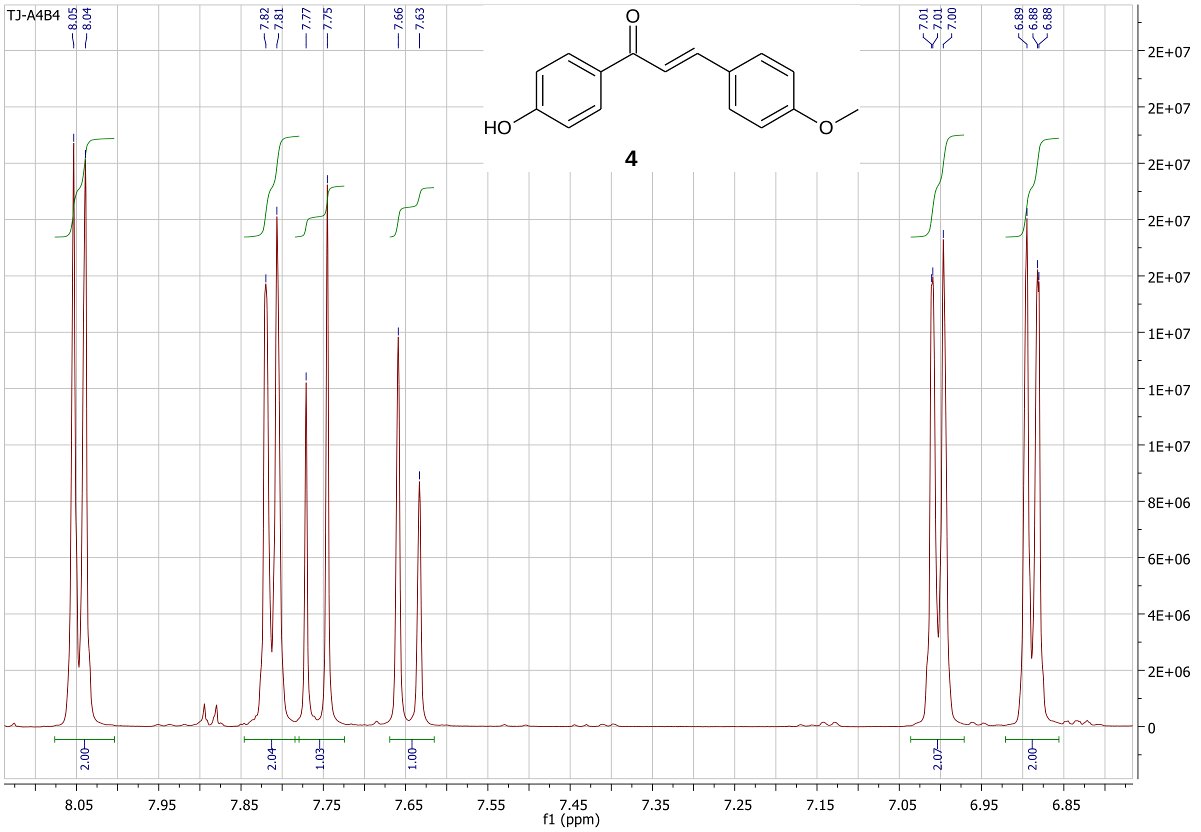

Figure S56. ^{13}C NMR spectrum of *trans*-4′-hydroxy-4-methoxychalcone (**4**) (DMSO-d_6. 151 MHz)

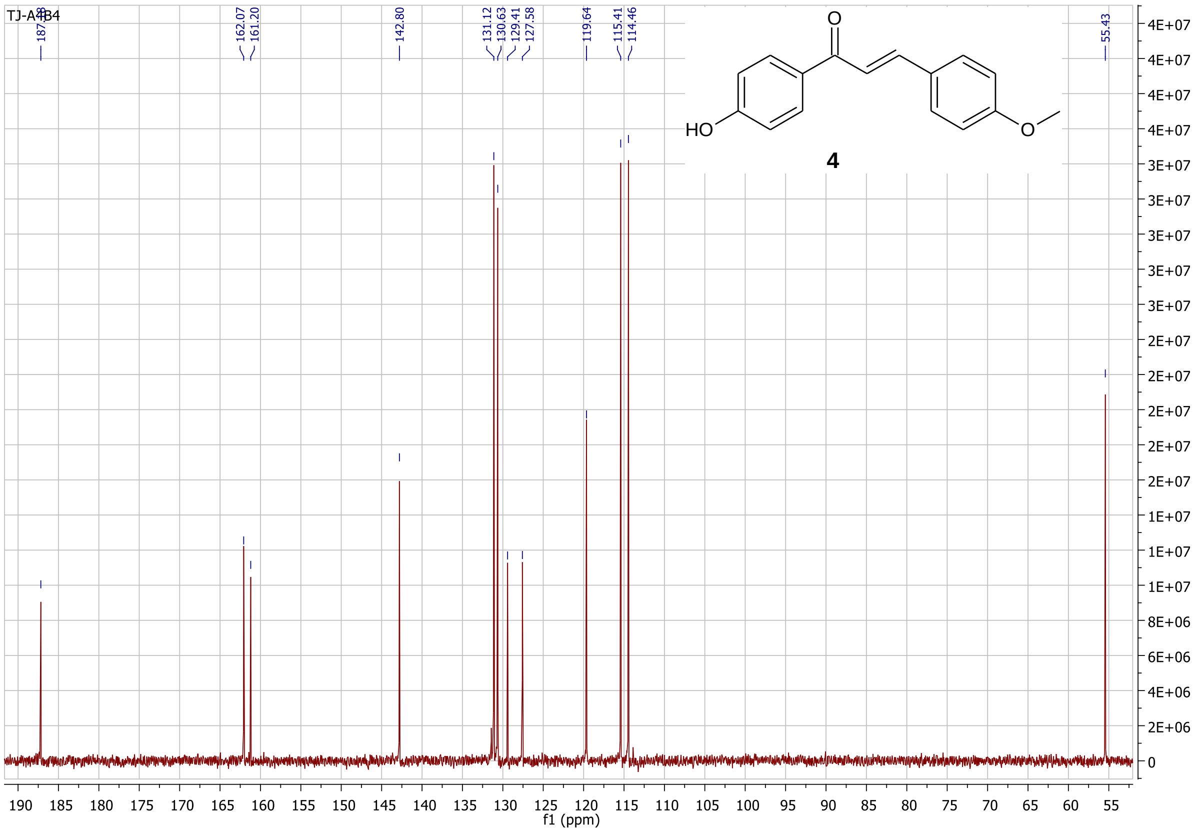

Figure S57. COSY NMR spectrum of of *trans*-4′-hydroxy-4-methoxychalcone (**4**) (DMSO-d_6. 600 MHz)

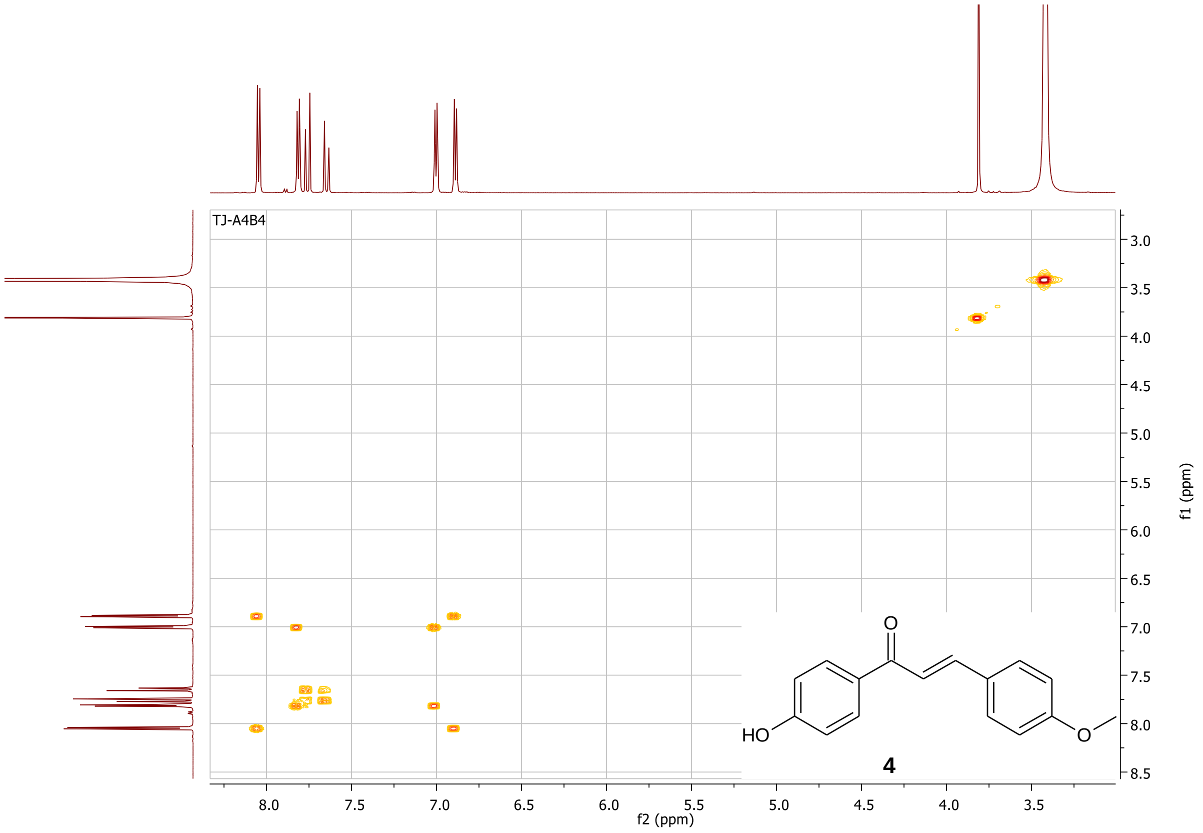

Figure S58. HMQC NMR spectrum of of *trans*-4′-hydroxy-4-methoxychalcone (**4**) (DMSO-d_6. 600 MHz)

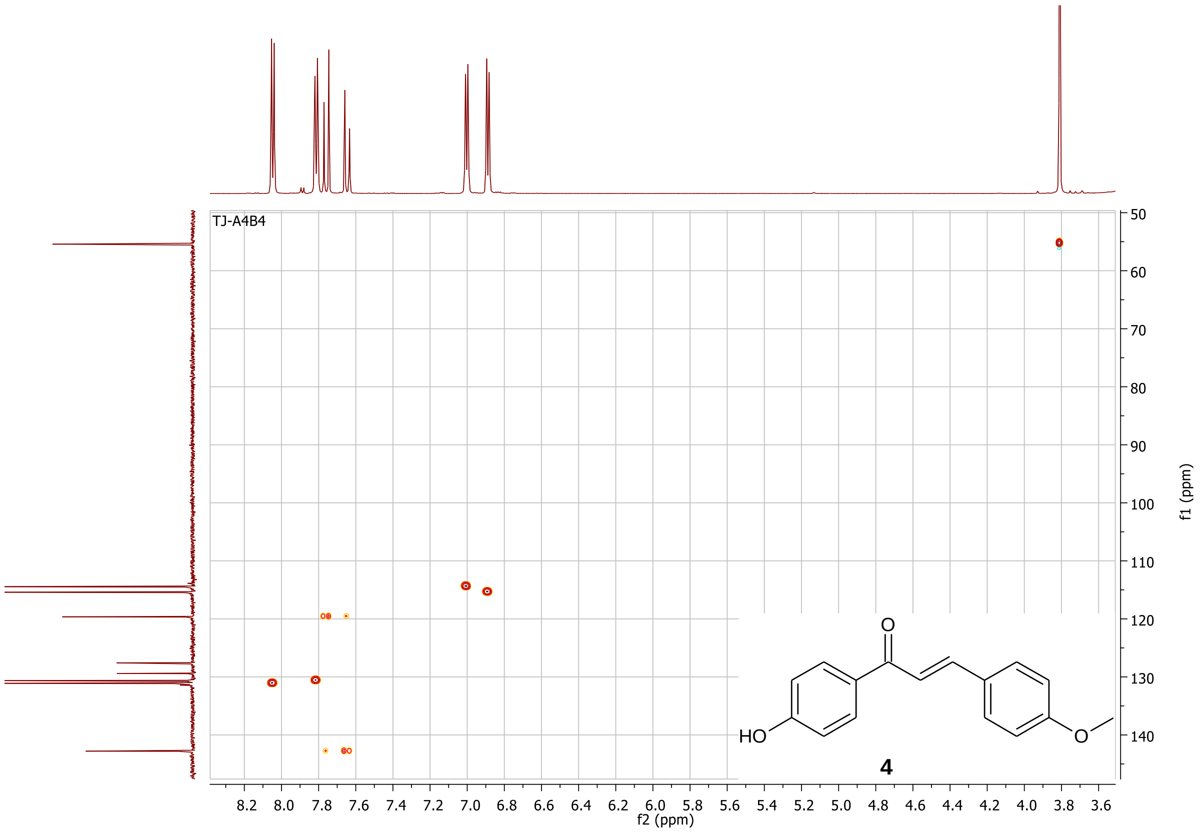

Figure S59. HMBC NMR spectrum of of *trans*-4′-hydroxy-4-methoxychalcone (**4**) (DMSO-d_6. 600 MHz)

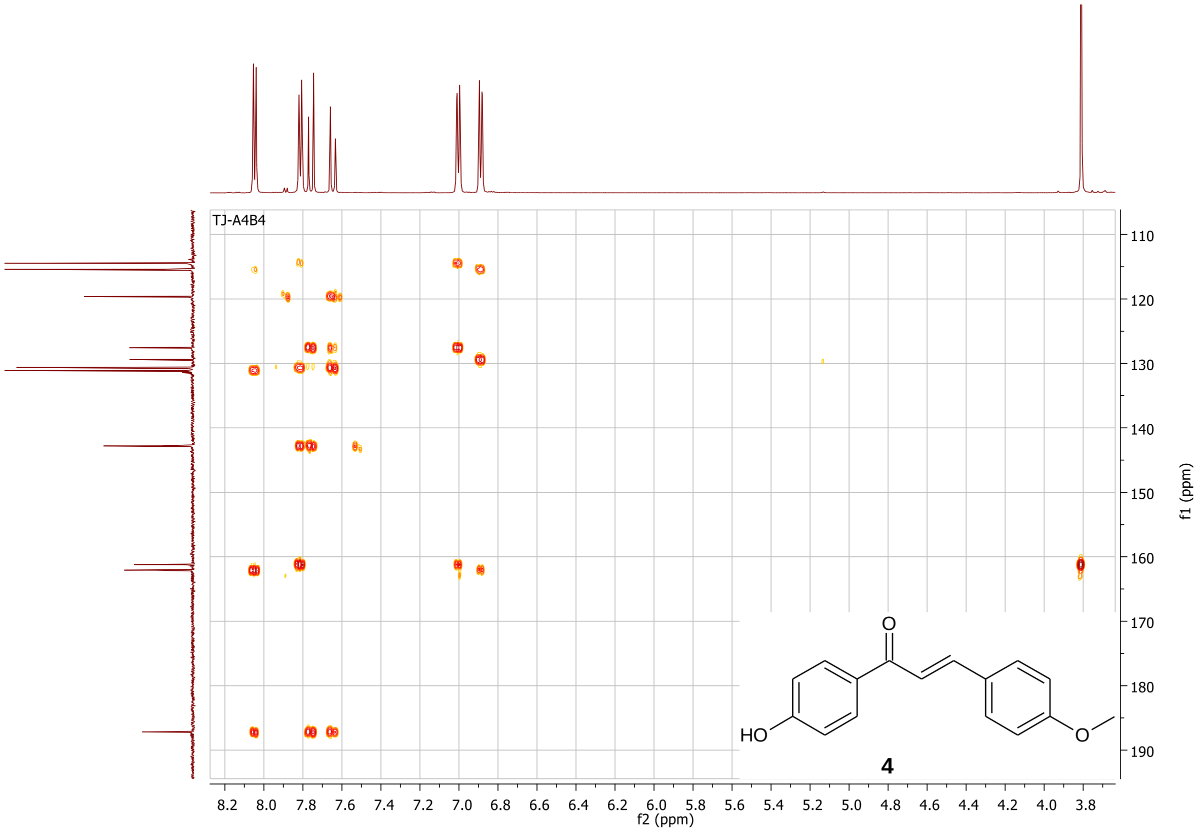

Figure S60. ^{1}H NMR spectrum of *cis*-4′-hydroxy-4-methoxychalcone (**4a**) (DMSO-d_6. 600 MHz)

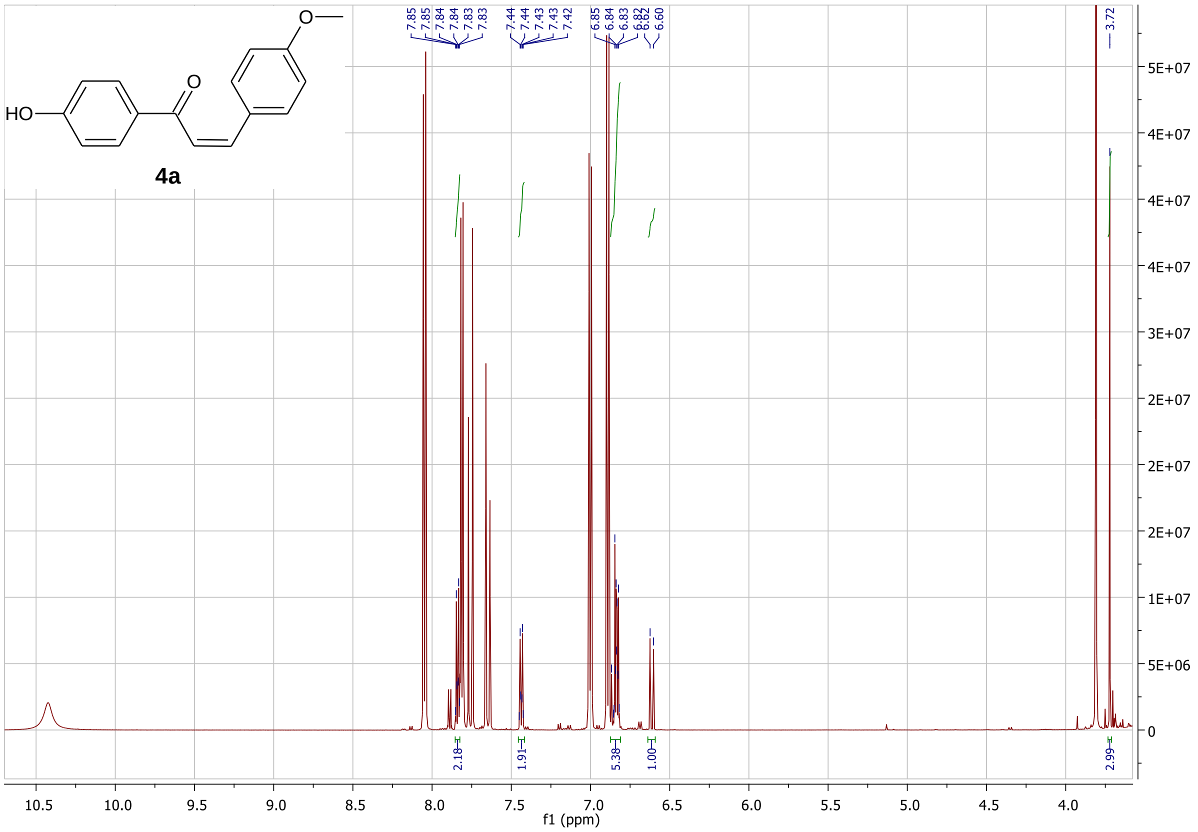

Figure S61. Part of the ^{1}H NMR spectrum of *cis*-4′-hydroxy-4-methoxychalcone (**4a**) (DMSO-d_6. 600 MHz)

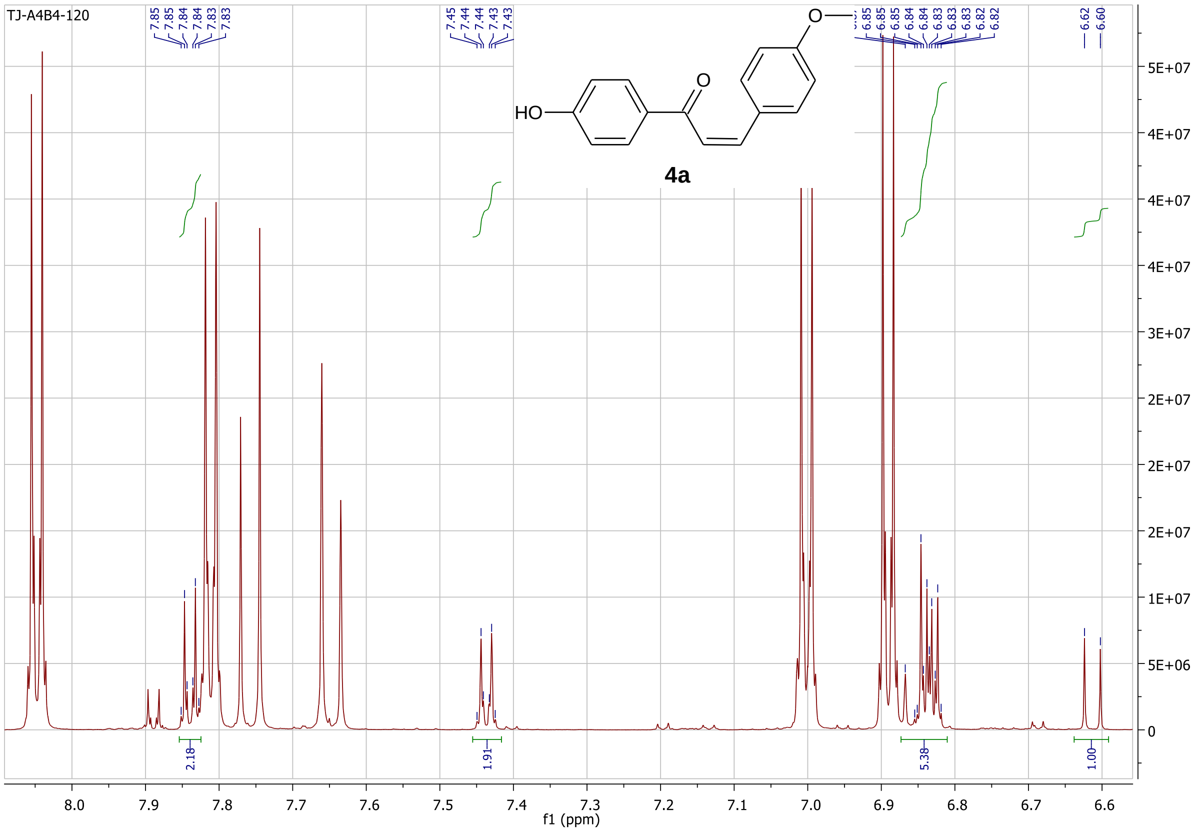

Figure S62. ^{13}C NMR spectrum of *cis*-4′-hydroxy-4-methoxychalcone (**4a**) (DMSO-d_6. 151 MHz)

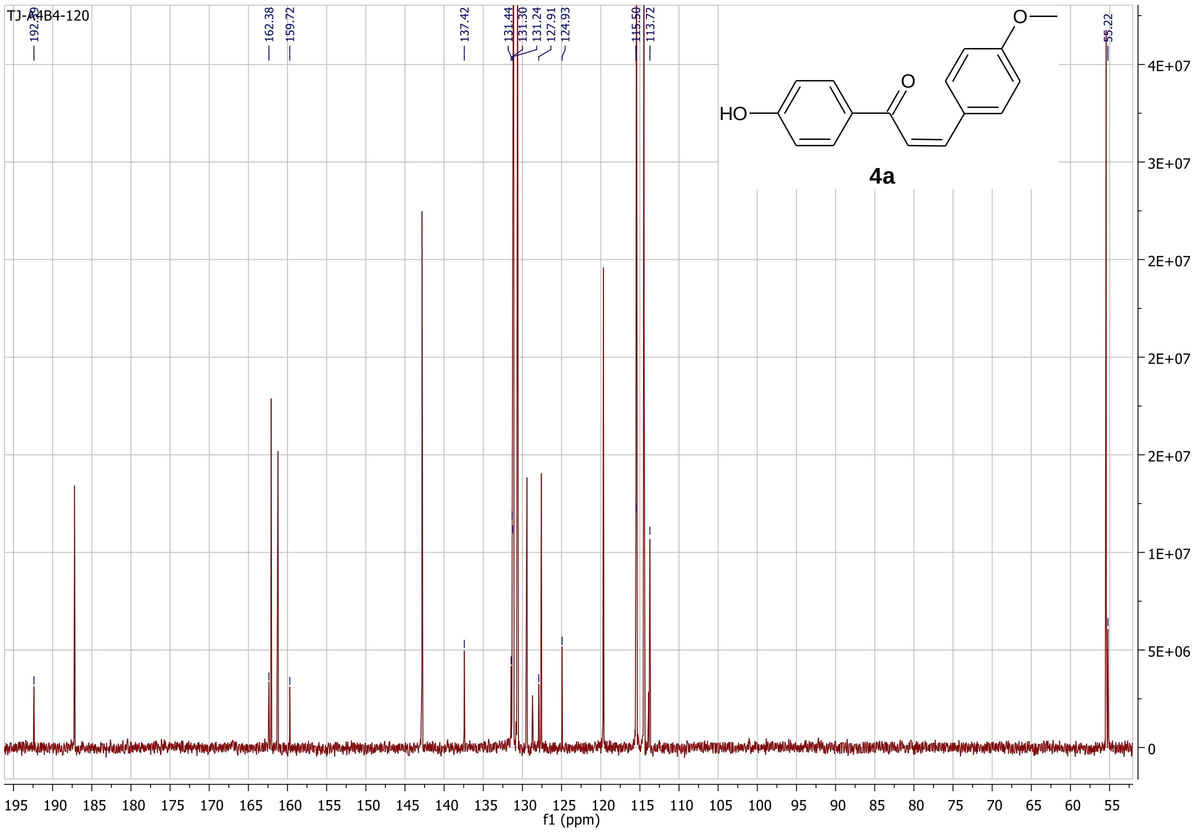

Figure S63. COSY NMR spectrum of of *cis*-4′-hydroxy-4-methoxychalcone (**4a**) (DMSO-d_6. 600 MHz)

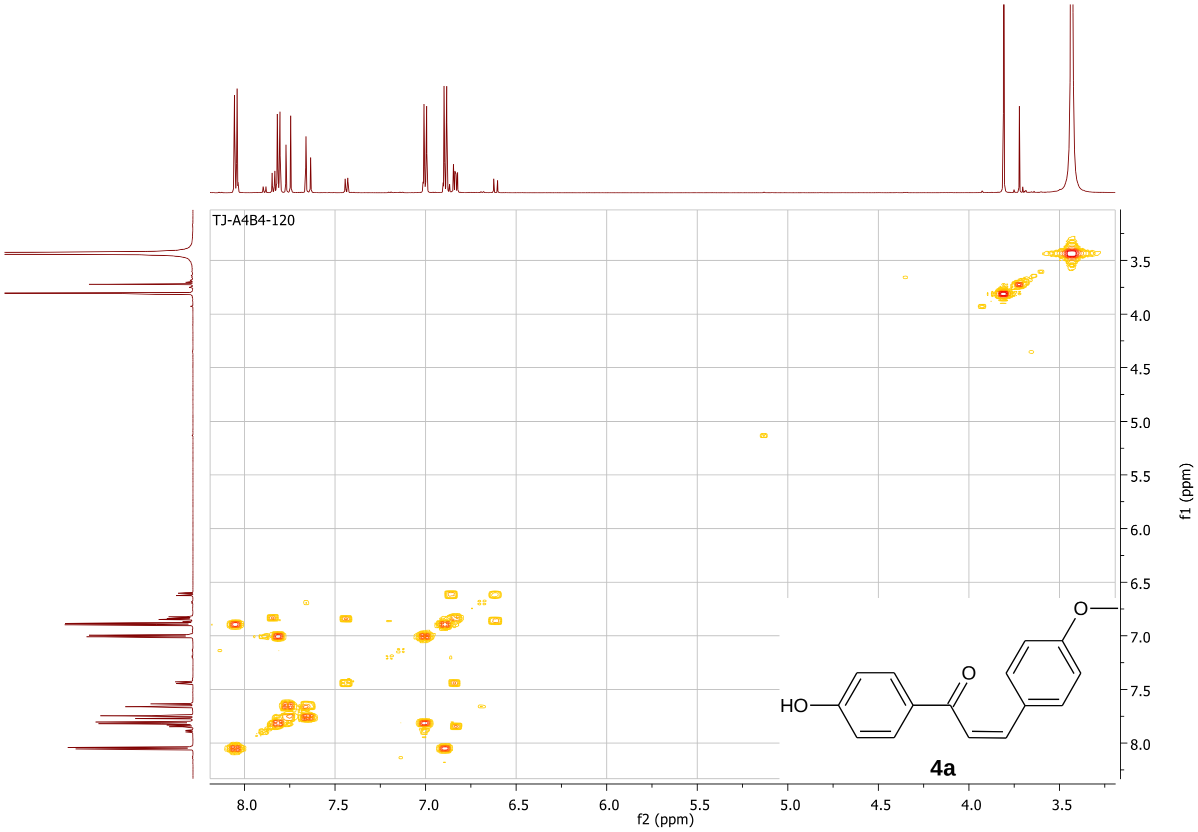

Figure S64. HMQC NMR spectrum of of *cis*-4′-hydroxy-4-methoxychalcone (**4a**) (DMSO-d_6. 600 MHz)

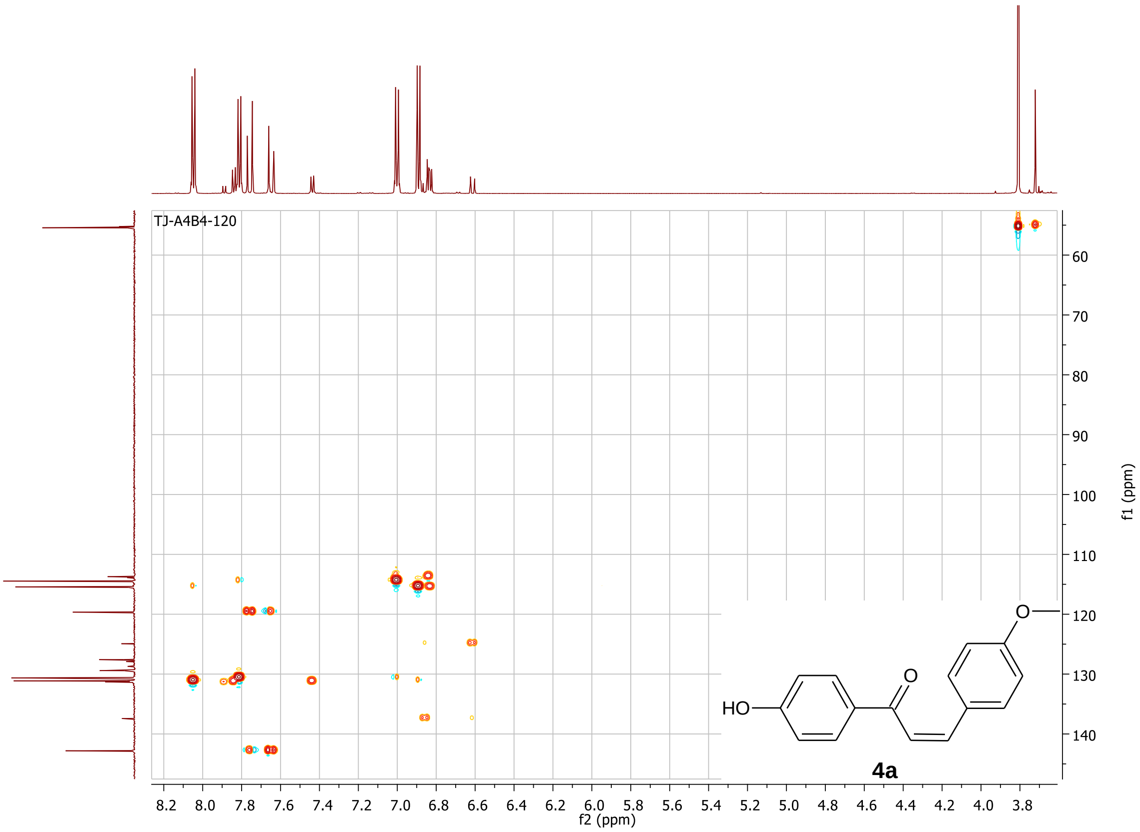

Figure S65. HMBC NMR spectrum of of *cis*-4′-hydroxy-4-methoxychalcone (**4a**) (DMSO-d_6. 600 MHz)

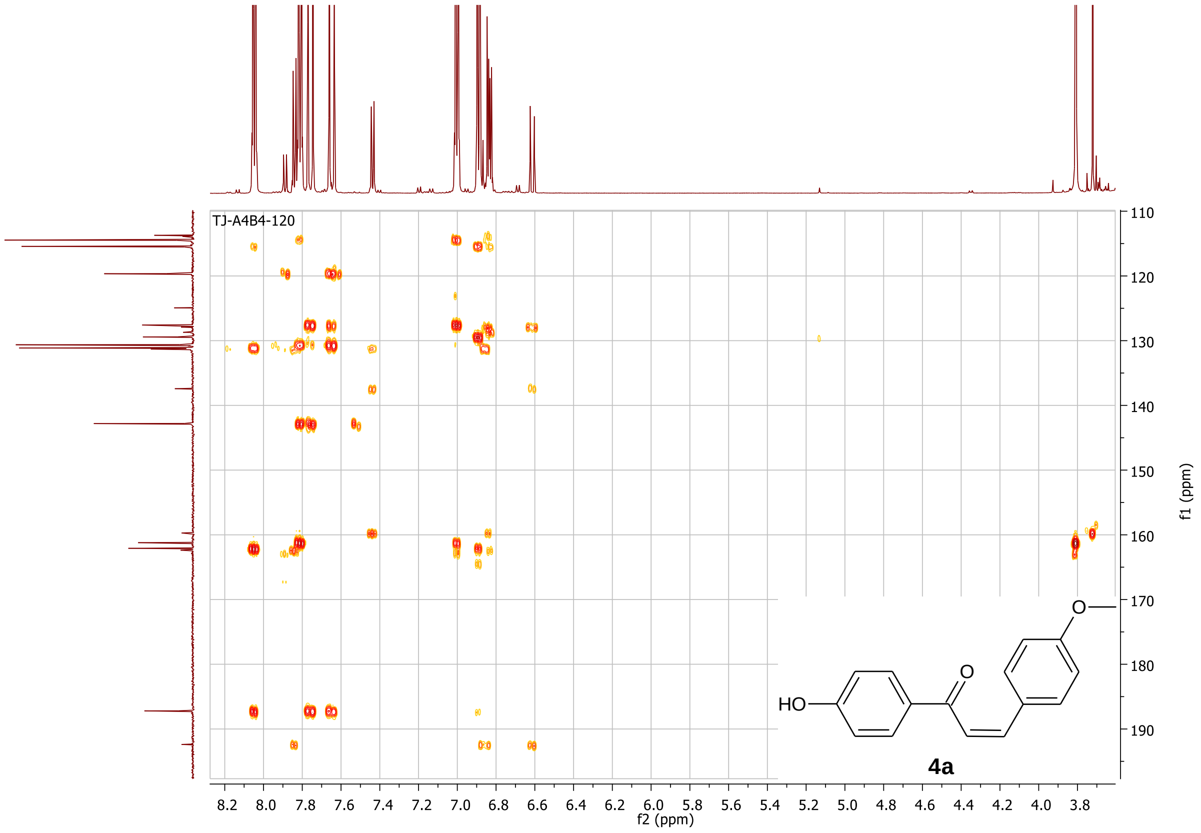

Figure S66. ^{1}H NMR spectrum of 4′-hydroxy-4-methoxydihydrochalcone (**4b**) (CDCl$_3$. 600 MHz)

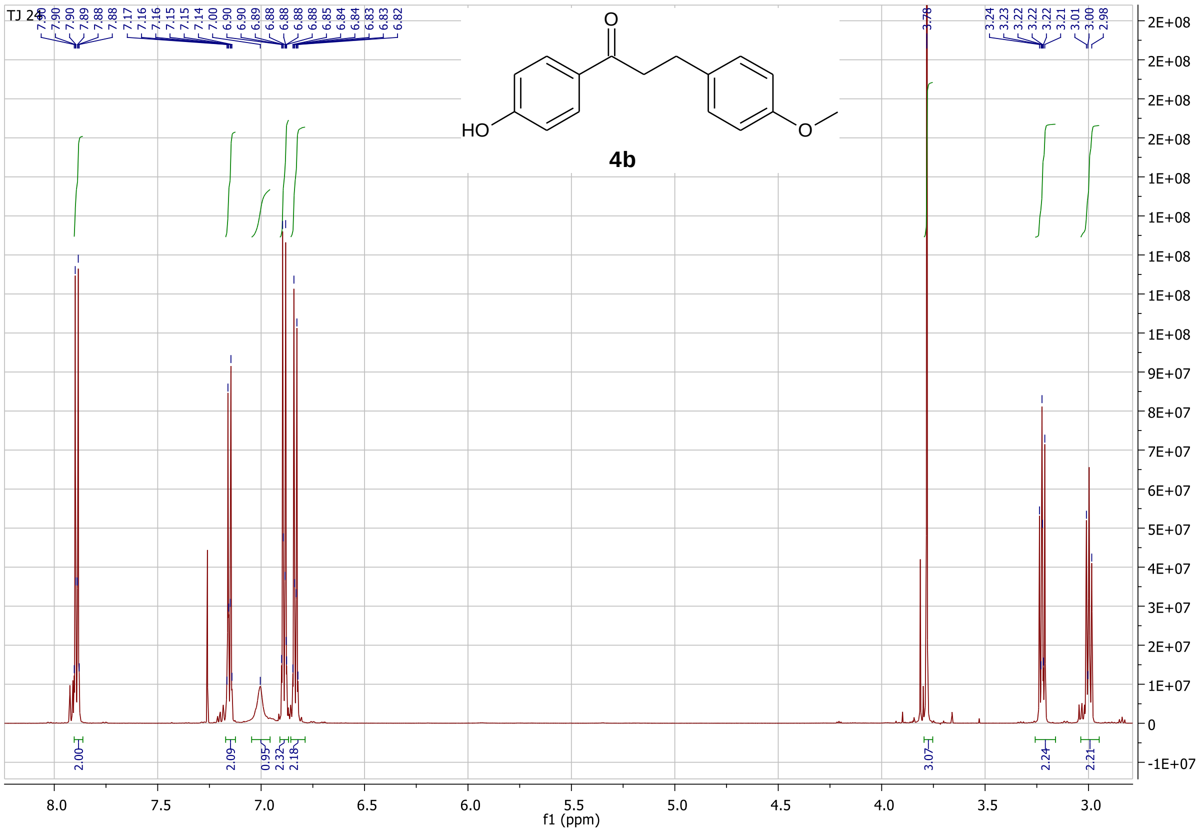

Figure S67. Part of the ^{1}H NMR spectrum of 4′-hydroxy-4-methoxydihydrochalcone (**4b**) ($CDCl_3$. 600 MHz)

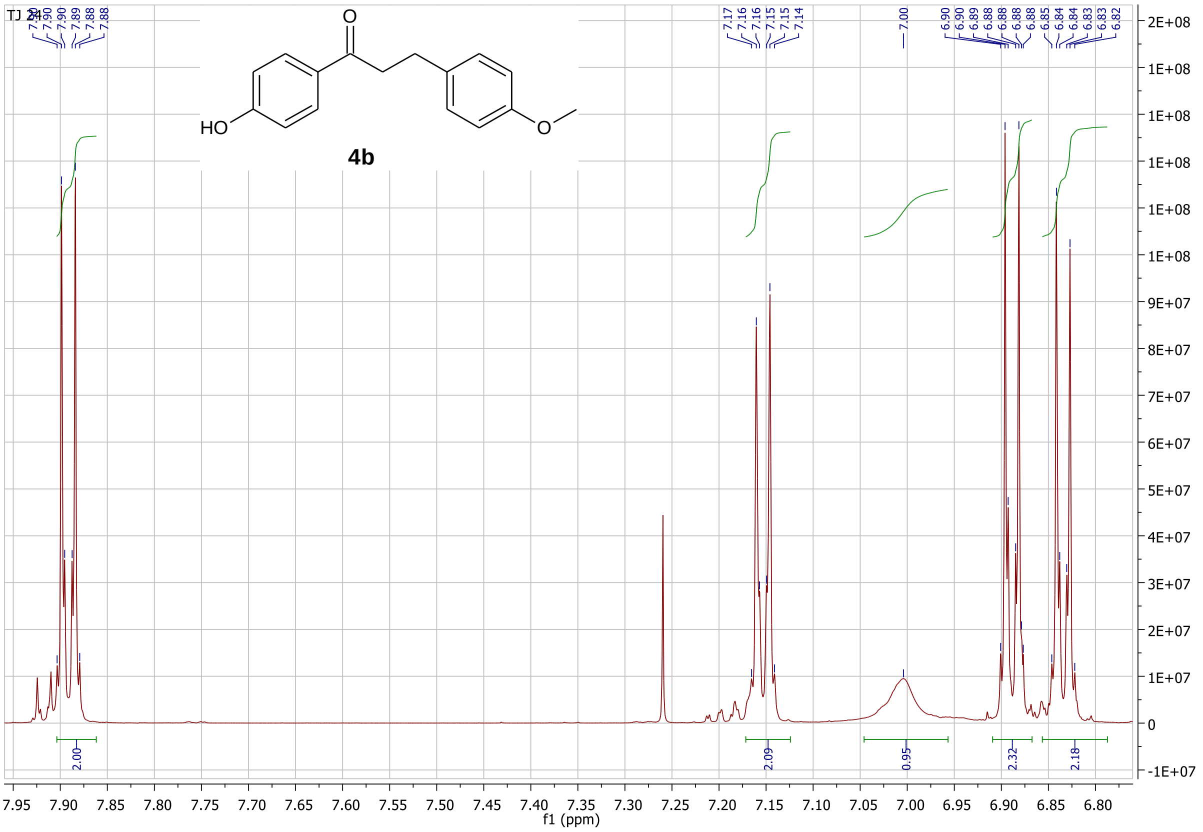

Figure S68. ^{13}C NMR spectrum of 4′-hydroxy-4-methoxydihydrochalcone (**4b**) ($CDCl_3$. 151 MHz)

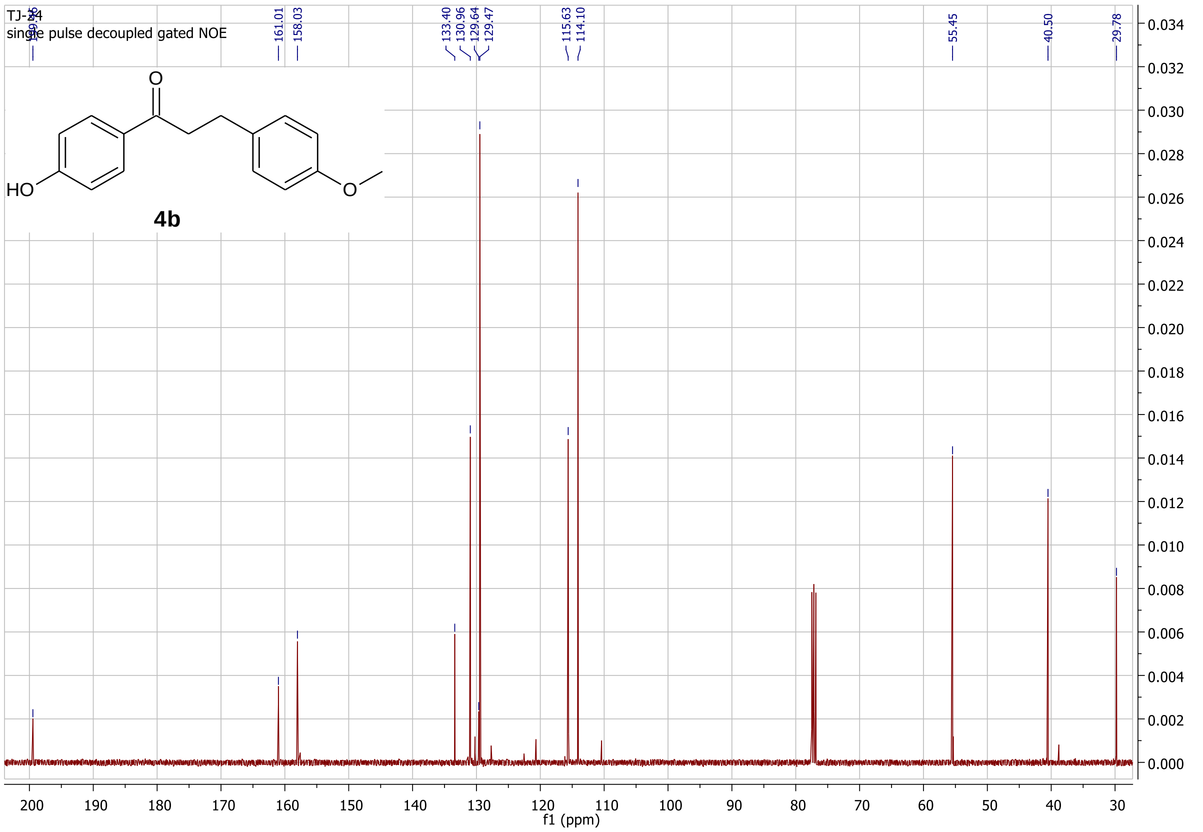

Figure S69. COSY NMR spectrum of of 4′-hydroxy-4-methoxydihydrochalcone (**4b**) (CDCl₃. 600 MHz)

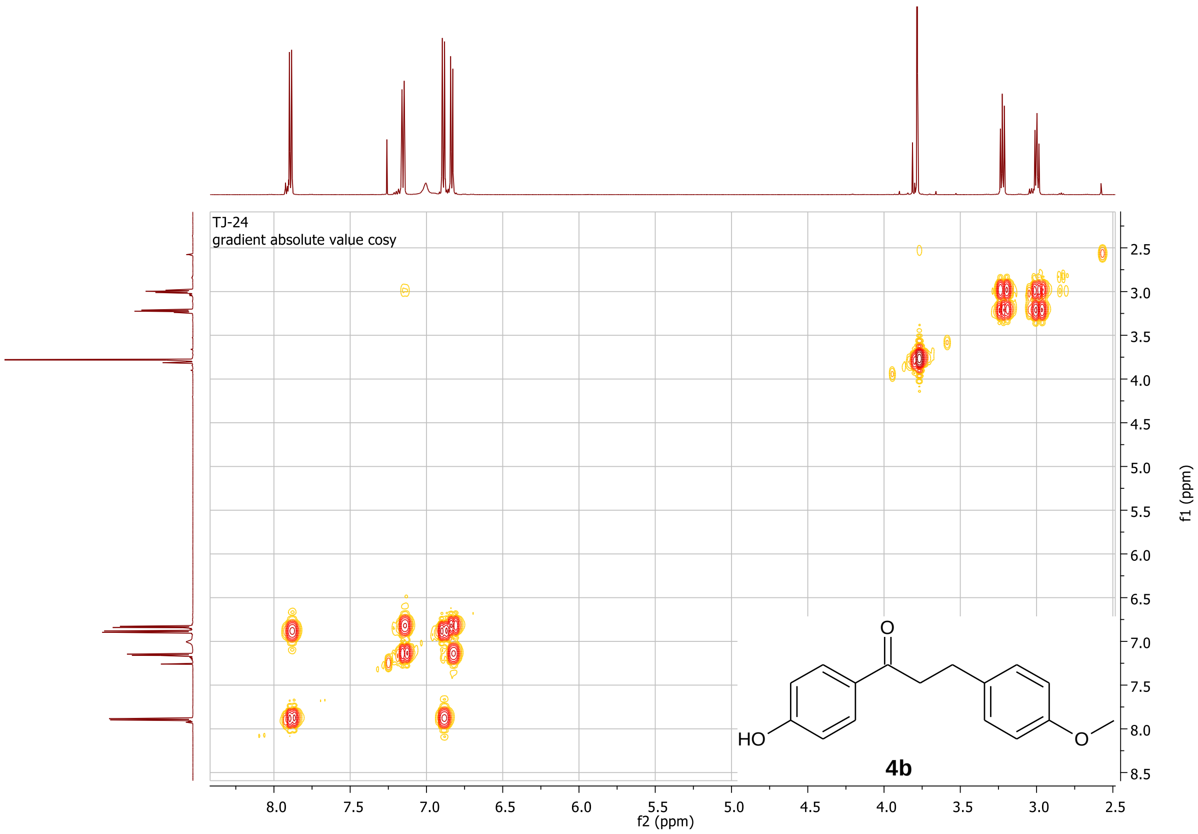

Figure S70. HMQC NMR spectrum of of 4′-hydroxy-4-methoxydihydrochalcone (**4b**) (CDCl₃. 600 MHz)

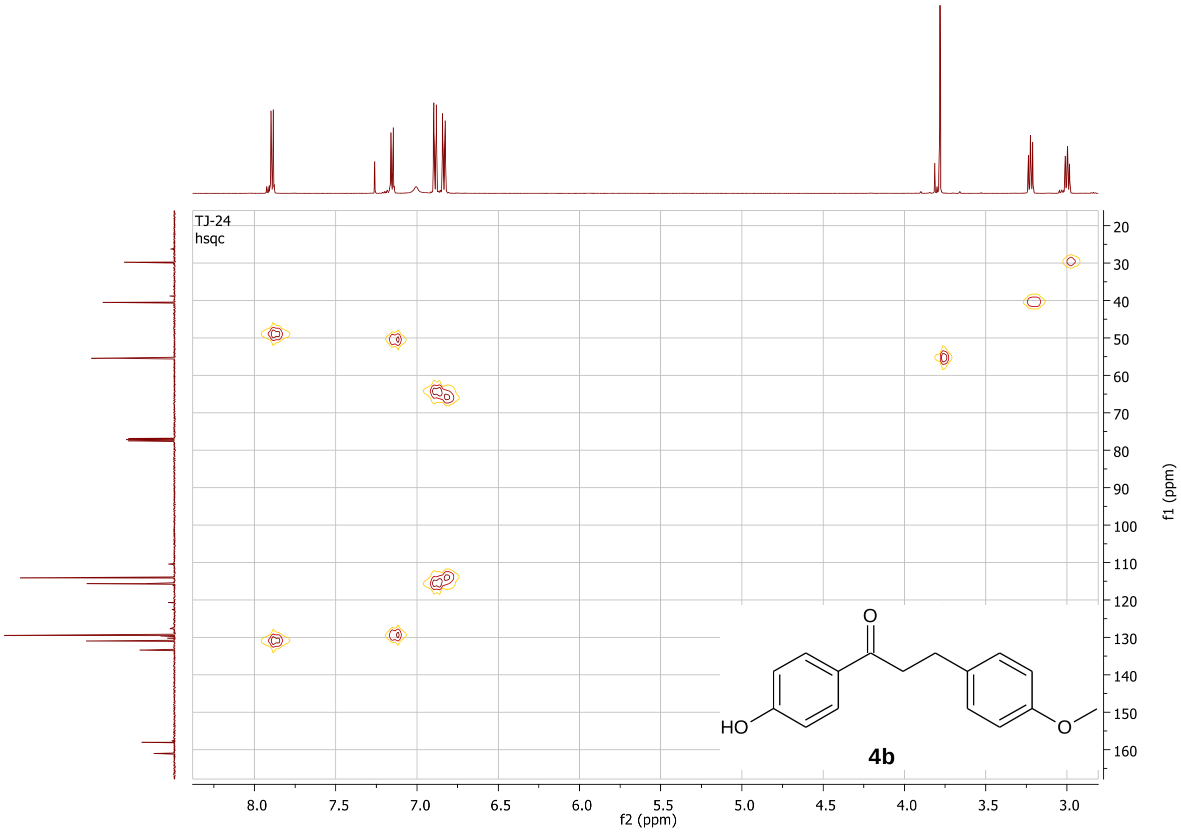

Figure S71. HMBC NMR spectrum of of 4′-hydroxy-4-methoxydihydrochalcone (**4b**) ($CDCl_3$. 600 MHz)

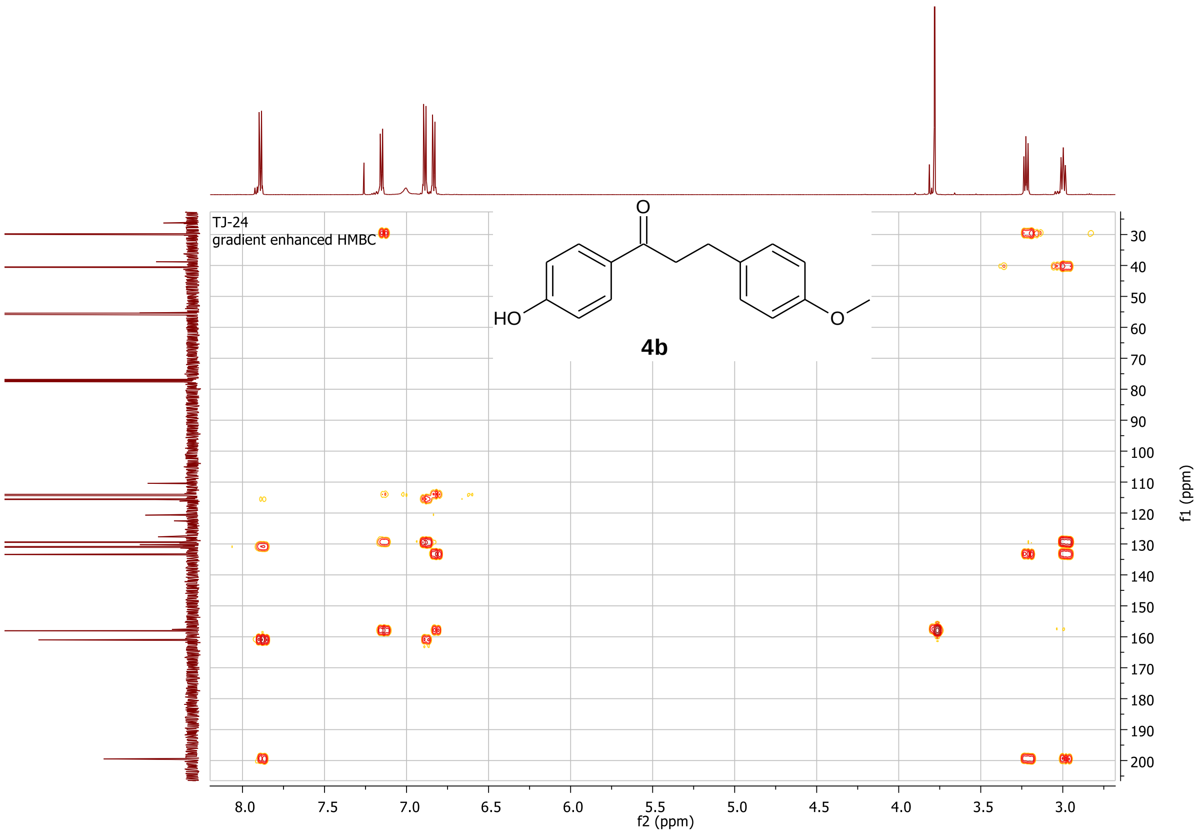

Supplement: Supplementary file 1 [file ijms-25-10735-s001.zip › ijms-3150492-supplementary.pdf]
